# Transcriptomics Analysis Indicates Trifarotene Reverses Acne-Related Gene Expression Changes

**DOI:** 10.3389/fmed.2021.745822

**Published:** 2021-10-22

**Authors:** Brigitte Dreno, Rajeev Chavda, Valerie Julia, Amir Khammari, Sandrine Blanchet-Réthoré, Jayendra Kumar Krishnaswamy

**Affiliations:** ^1^Dermatology Department, CHU Nantes, Nantes, France; ^2^Galderma R&D, La Tour-de-Peilz, Switzerland; ^3^Syneos Health, Translational Sciences, Sophia Antipolis, France

**Keywords:** acne (acne vulgaris), pathophysiology, bioinformatics, gene expression, cellular expression, trifarotene, transcriptomics

## Abstract

**Background and Objectives:** Trifarotene is a topical retinoid selective for retinoic acid receptor gamma that was recently approved for treatment of acne vulgaris. We performed a gene expression analysis to identify the molecular and cellular impact of trifarotene treatment on acne papules.

**Methods:** In this open-label prospective study, subjects with moderate inflammatory acne of the back were treated with trifarotene 0.005% or vehicle cream on dedicated areas for 27 days, and 4 biopsies were collected from each subject (1 from skin without a visible acne lesion and three at the site of an acne papule: one baseline, one after vehicle treatment, and one after trifarotene treatment). Large scale gene expression profiling of the biopsies was performed using Affymetrix technology, treatment-specific gene expression profiles were generated using statistical modeling, and pathway analysis was performed. Using single-cell RNAseq data, *in silico* deconvolution of transcriptomics data was performed to identify cellular signatures.

**Results:** We discovered a unique set of 67 genes modulated by trifarotene that are primarily involved in cellular migration, inflammation, and extracellular matrix reorganization. Changes in cellular expression were similar in both trifarotene-treated and spontaneously-resolving lesions. However, only trifarotene treatment impacted SPP1^+^ macrophages, a subset of highly proliferative macrophages recently identified in fibrotic tissue.

**Conclusions:** These results show that trifarotene has a novel action in acne treatment by affecting epidermal and immune components of acne pathogenesis.

## Introduction

Acne vulgaris is a chronic inflammatory skin condition that develops from a combination of four critical factors: (i) increased sebum production, (ii) abnormal keratinization and cornification of the sebaceous follicular duct, (iii) colonization of the hair follicles by microbes—putatively *Cutibacterium*, and (iv) complex inflammatory mechanisms that involve both innate and acquired immunity (1, 2). Trifarotene is the first topical retinoid molecule to be marketed in almost two decades, and it has demonstrated good efficacy in treating facial and truncal acne (3). Topical retinoids are known to increase epidermal turnover and normalize keratinization in acne. The American Academy of Dermatology acne management guidelines state that “retinoids are the core of topical therapy for acne because they are comedolytic, resolve the precursor microcomedone lesion, and are anti-inflammatory” (4). Topical retinoids have been shown to block several inflammatory pathways activated in acne including Toll-like receptor activation, leukocyte migration, and AP-1 signaling (5–8). However, the anti-inflammatory mechanisms of retinoids have been mainly demonstrated using *in vitro* and *in vivo* models of inflammation and in clinical studies (9–12). To date, there have been very few investigations into the gene expression changes and pathways modulated by retinoid treatment in acne. Further, the effects of retinoids on innate and adaptive immune responses are not well-characterized. Much that happens during the life cycle of acne lesions–whether spontaneously occurring or changes due to efficacious acne treatment–remains mysterious.

To enhance understanding of the mode of action of trifarotene in acne, skin biopsies were collected from lesional and non-lesional skin of patients with acne vulgaris treated with trifarotene or vehicle, and whole transcriptome gene expression profiling was performed. In addition, gene signatures of the major cell types present in skin biopsies were determined from a publicly available single cell RNAseq dataset (13), and cellular expression was compared in non-lesional, active acne lesion, and resolved lesions (either spontaneously or due to trifarotene treatment). The aim of this study was to determine which genes and molecular pathways, modulated in acne, are specifically affected by trifarotene treatment. To our knowledge, this is the first study to make this type of comparison.

## Methods

### Study Description

**Figure 1:**
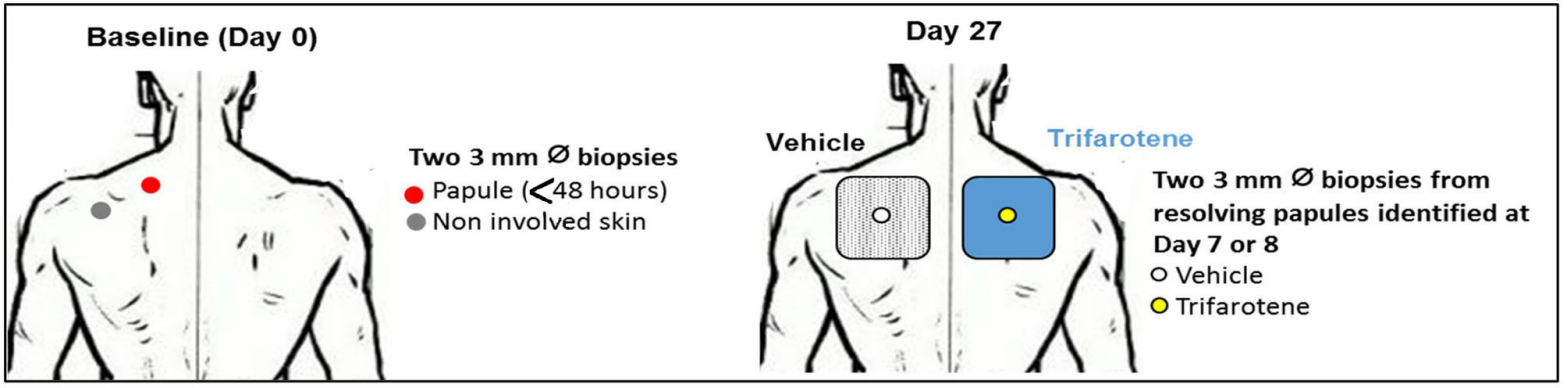
Location of biopsies at Day 0 and Day 27.

This was a *post-hoc* analysis of an additional subset of data collected as part of Aubert et al. (14) from 9 patients aged 18–35 with acne vulgaris (EUDRACT No. 2012-001943-36). In the 4-week open-label clinical study, subjects received once-daily applications of trifarotene 0.005% cream and vehicle cream on the back. To be eligible, subjects had moderate inflammatory acne on the back at screening, defined by Acne Lesion Scale score of 2–4 for the whole back, with at least one area scored at 2, and a maximum of three nodules. Subjects were excluded if they had active skin disease or inflammation other than acne, underlying known conditions which could interfere with study results, history of allergy to local anesthetics and/or topical antiseptics, history of bleeding disorder, or pregnancy/lactation (females). Predefined washout periods were mandated for acne treatments, immunosuppressants, immunomodulators, systemic corticosteroids, and isotretinoin. Each patient had four biopsies performed (Figure 1), one from skin without visible acne lesion (non-involved skin) and three at the site of acne papule, with the following timing and topical application: one prior to treatment (day 0), one after vehicle treatment (day 27), and one after trifarotene treatment (day 27). Biopsies were stored in RNAlater TissueProtect Tubes (Qiagen, Les Ulis, France). All subjects provided written informed consent prior to biopsies. The study was conducted in accordance with Declaration of Helsinki principles and ICH Guideline for Good Clinical Practice and received approval from the ethics committee of Brest, France (reference CPP Quest 6-755) (14).

### Bioinformatics Analysis

Data analysis was performed using Array Studio software (Omicsoft Corporation, Cary, NC, USA) and custom R scripts. Affymetrix U133 Plus 2.0 chips (GeneChip™, Santa Clara, CA, USA) were normalized using the multi-array average (RMA) method (15). Low expression probe-sets were filtered. Only Affymetrix probe-sets with expression ≥2^6^ in at least five samples in one condition were selected for further statistical analysis, resulting in data for 36,245 out of the 54,675 probe-sets present on the HG U133 Plus 2.0 array. Mean expression levels were obtained by calculating the geometric means of the RMA-normalized data. A linear model was fitted to the normalized data, and statistical testing of the comparisons of interest were performed using a moderated *t*-test. A paired-test was used when applicable. *P*-values were adjusted for multiple testing using the Benjamini-Hochberg false discovery rate (FDR) method (16). Candidate differentially expressed genes were selected using a combination of fold-change and FDR: genes with absolute fold change >2 and FDR <0.05 were considered as candidate differentially expressed genes.

Functional and network analyses were generated with the Ingenuity Pathway Analysis (IPA) software (QIAGEN Inc., https://www.qiagenbioinformatics.com/products/ingenuity-pathway-analysis) (17). IPA was used to identify canonical pathways, diseases and functions, and gene networks related to differentially expressed genes in acne lesions and post-lesional skin after treatment with trifarotene or vehicle. In addition, gene set enrichment analysis was performed with the R package FGSEA (version 1.12.0) using the hallmark and reactome gene sets (18).

### Single-Cell Analysis and Cell Marker Definition

We used single-cell RNAseq data from Hughes et al. to determine the major cell types present in skin biopsies and the associated cell markers (13). Acne and normal samples from GSE150672 were downloaded and analyzed using the Seurat package (19). Data were normalized, scaled, and log-transformed. The most variable genes were selected based on their expression and dispersion. To reduce dimensionality, principal component analysis (PCA) was applied. The first eight principal components were used to cluster the cells using the graph-based approach FindClusters function in Seurat, with a resolution parameter of 0.4. Graphical representation of cell clusters was achieved using UMAP. We then used a combination of known markers and differentially expressed genes to characterize cell clusters. Major cell type markers retained for scoring analysis included: B cells: MS4A1, CD79A; Fibroblasts: DCN, COL6A2, APOD, CFD, IGFBP5, COL1A2, COL1A1, COL3A1; Keratinocytes: KRT5, KRT1, KRT14, KRT15, S100A2, KRT6A, HOPX, KRT10, DSP; Langerhans cells: CD207; Mast cells: CPA3, IL1RL1, CTSG, TPSAB1, GATA2; Melanocytes: MLANA, MITF, PMEL, DCT; Myeloid cells: CD68, CTSS; Schwann cells: SCN7A; T cells: CD3D, TRBC2, IL7R, PTPRC, CXCR4; Venular cells: SELE, CD93, TM4SF1, A2M, RCAN1; Vascular smooth muscle cells (VSMC): TAGLN, RGS5, MYH11, ACTA2, MYL9.

### Macrophage Gene Signatures

Gene markers specific to each class of macrophage were derived from the literature. For M0, M1 and M2 macrophages, we used markers as described in Newman et al. (20). For SPP1 activated macrophages, we used the markers from Morse et al. (21). Final markers were as listed.

M0 macrophage: ACP5, BHLHE41, C5AR1, CCDC102B, CCL22, CCL7, COL8A2, CSF1, CXCL3, CXCL5, CYP27A1, DCSTAMP, GPC4, HK3, IGSF6, MARCO, MMP9, NCF2, PLA2G7, PPBP, QPCT, SLAMF8, SLC12A8, TNFSF14, VNN1. M1 macrophage: ACHE, APOBEC3A, APOL3, APOL6, ARRB1, CCL19, CCL5, CCR7, CD38, CD40, CHI3L1, CXCL10, CXCL11, CXCL13, CXCL9, CYP27B1, DHX58, HESX1, IDO1, IFI44L, IL2RA, KIAA0754, KYNU, LAG3, LAMP3, LILRA3, LILRB2, NOD2, PLA1A, PTGIR, RASSF4, RSAD2, SLAMF1, SLC2A6, SOCS1, TLR7, TNFAIP6, TNIP3, TRPM4. M2 macrophage: AIF1, ALOX15, CCL13, CCL14, CCL23, CD209, CD4, CFP, CLEC10A, CLEC4A, CRYBB1, FES, FRMD4A, FZD2, GSTT1, HRH1, HTR2B, MS4A6A, NME8, NPL, P2RY13, PDCD1LG2, RENBP, WNT5B. SPP1 macrophage: MERTK, CD14, SPP1, CD68, LYZ.

### Cell Type Scoring

Each cell type gene signature was used to define a cell type average gene expression in each of our clinical study samples, calculated as the average of the log2 expression values of these markers. Boxplots were used to visualize the distribution of cell type gene expression scores between clinical groups. Statistical significance was assessed using Wilcoxon rank sum test. Graphs were generated using the ggpubr data visualization library.

## Results

### Acne and Trifarotene Signatures

**Figure 2:**
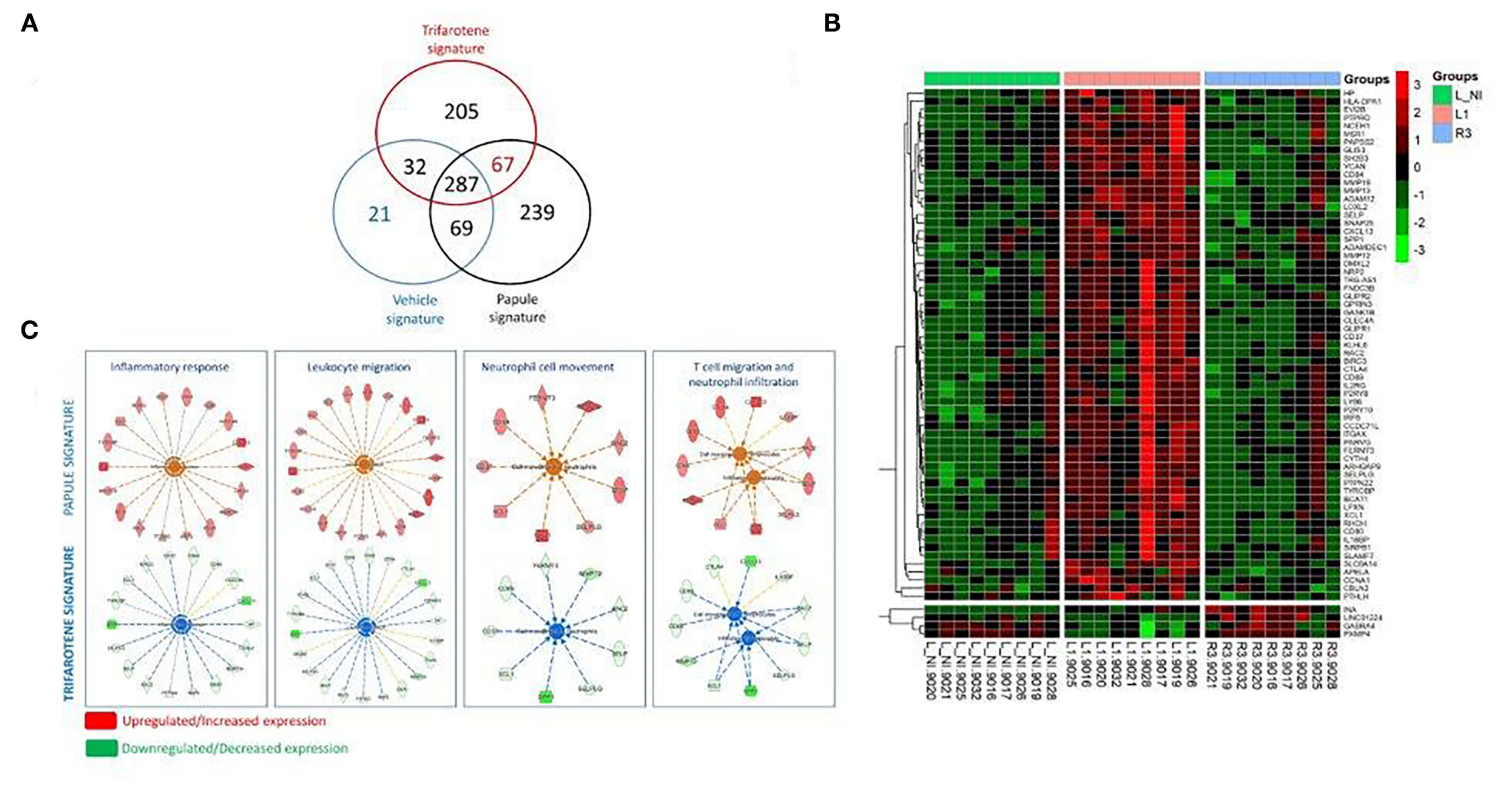
Comparison of gene expression in acne papules, spontaneously resolved papules, and trifarotene treated papules. **(A)** Venn diagram highlighting the number of candidate differentially expressed genes. *Vehicle signature*: biopsy of a papule treated with vehicle vs. papule at baseline; *papule signature*: biopsy of a papule at baseline compared to non-involved skin; *trifarotene signature*: biopsy of a papule after trifarotene treatment vs. papule at baseline. **(B)** Heatmap highlighting gene expression changes between non-involved skin, papule at baseline, and papule after trifarotene treatment, for the 67 genes specifically regulated by trifarotene. Genes were clustered using average linkage and correlation distance. Gene expression levels were scaled by gene (green = lower gene expression, red = higher gene expression). No clustering of the sample was performed. Color bars at the top displays the clinical group of each sample: L-NI, non-involved skin; L1, papule at baseline; R3, papule after treatment with trifarotene; **(C)** IPA of the 67 genes revealed upregulation of pathways associated with inflammatory response, leukocyte migration, neutrophil movement, and T cell migration in the papule signature that are concomitantly downregulated following trifarotene treatment.

To assess the mode of action of trifarotene, we first derived gene expression profiles of (1) acne lesion at baseline (papule signature), (2) previously lesional skin after spontaneous resolution of the papule (vehicle signature), and (3) previously lesional skin after resolution of a papule following trifarotene treatment (trifarotene signature). Papule signature was defined as genes differentially expressed when comparing the papule biopsy before treatment vs. non-involved skin biopsy, spontaneous resolution of a papule (vehicle signature) was defined as genes differentially expressed between vehicle treated area and a papule biopsy before treatment, and trifarotene signature as genes differentially expressed between trifarotene treated area and a papule biopsy before treatment. The number of unique annotated genes found significantly differentially expressed in each comparison is presented in the Venn diagram (Figure 2A).

Analysis of the acne gene expression signature showed that both cellular movement and immune cell trafficking were increased in acne lesions. IPA canonical pathway analyses showed strong activation of TREM1 signaling, crosstalk between dendritic and natural killer cells, leukocyte extravasation signaling, dendritic cell maturation, neuroinflammatory pathways, and pattern recognition receptors involved in recognition of bacteria and viruses (*z*-score > 2.5, *P* < 0.001 for all these pathways). IPA diseases and functions analyses of the acne gene signature also revealed that cell migration and activation were among the major processes upregulated in acne papules (Supplementary Tables 1, 2). Further, the acne signature also showed a decrease in liver X receptor/retinoid X receptor (LXR/RXR) signaling (*z*-score = −1.78, *P* < 0.001), suggesting inhibition of retinoid X receptor pathways (and, in turn, keratinocyte differentiation) in acne papules (22). Trifarotene appeared to reverse many of the pathways associated with acne lesions including LXR/RXR signaling (*z*-score = 1.46 for Trifarotene gene signature) and induced a concomitant reduction in neuroinflammation pathway, leukocyte extravasation signaling, cellular movement, and immune cell trafficking (Supplementary Tables 1, 2). In line with the IPA analysis, gene set enrichment analyses revealed that Trifarotene treatment indeed downregulates inflammatory modules (“*inflammatory response,” “TNFA signaling via NF-kB,” “neutrophil degranulation,” “chemokines and chemokine receptors”*) and tissue remodeling (“*collagen degradation,” “degradation of the extracellular matrix,” “extracellular matrix organization”*) (Supplementary Figure 1).

### Spontaneously Resolving Papules Signature vs. Acne and Trifarotene Signatures

When gene expression in acne lesions that spontaneously resolved (i.e., vehicle signature) was analyzed, the pattern included changes in pathways and processes similar to those seen with trifarotene treatment. A total of 287 genes that were upregulated in acne papules were downregulated in both the trifarotene and vehicle signatures (Figure 2A). These data suggested that the downregulation of previously described acne signature pathways was not necessarily specific to trifarotene treatment (Figure 2A).

### Trifarotene-Specific Effects

A total of 67 genes were uniquely affected by trifarotene–these genes did not appear in the spontaneously resolving acne lesion signature (Figure 2A, Supplementary Table 3). Figure 2B presents a heatmap of the expression profiles of the 67 genes in non-involved skin, acne lesion, and trifarotene-treated skin. A majority of these genes were upregulated in acne lesions (as compared to non-involved skin) and were concomitantly downregulated by trifarotene treatment only (Figure 2B). The most significantly down-regulated genes included the chemokines CXCL13 (Fold-Change FC = −23.5, FDR = 0.0032) and XCL1 (FC = −2.02, FDR = 0.013), the phosphoglycoprotein osteopontin (SPP1, FC = −28.2, FDR = 0.0022) and the matrix metalloproteinases MMP12 and MMP13 (FC = −11.13, FDR = 0.061; FC = −4.3, FDR = 0.029, respectively). These genes influence inflammatory cell infiltration (including CXCL13, XCL1, and SPP1/osteopontin) and extracellular matrix reorganization (MMP12 and MMP13) (Supplementary Table 3). IPA analysis of the 67 genes (Figure 2C) revealed that an upregulation of pathways associated with inflammatory response, leukocyte migration, neutrophil movement, and T cell migration seen in the papule was reversed following trifarotene treatment (Figure 2C, lower panel, Supplementary Table 4 for statistical results and list of molecules involved in each pathway).

Of the 67 genes regulated by trifarotene, there were only four genes upregulated: long intergenic non-protein coding RNA 1224 (LINC01224), alpha-internexin (INA), gamma-aminobutyric acid receptor subunit alpha-4 (GABRA4) and peroxisomal membrane protein 4 (PXMP4) (Supplementary Table 3). There is limited information on the role of these genes on inflammation and skin homeostasis. LINC01224 has been recently described to promote cell proliferation and survival (23). GABRA4 and INA play a role in the nervous system with GABA4 being a major inhibitory neurotransmitter and INA is a neuronal specific intermediate filament playing a role in neuronal development. The impact of upregulation of these genes following trifrotene treatment is currently unknown but will be of interest in future studies.

To identify if these pathways were specific to trifarotene treatment, we analyzed the 69 genes uniquely regulated in spontaneously resolved lesions. The major downregulated genes in spontaneously resolved papules included inflammation related genes like chemokine CXCL10 and beta defensin 103 (DEFB103A/DEFB103B), transcobalamin-1 (TCN1), and epithelial-related genes including S100A7A, S100A9, SPRR3, SERPINB3, and SERPINB4 (Supplementary Table 5). Pathway analysis of these genes revealed that while spontaneous resolution of lesions is associated with decreasing inflammation (Supplementary Table 6), there was no impact on extracellular matrix organization in contrast to what was observed with trifarotene. The relative roles of CXCL10 and CXCL13 on recruitment of specific T cell subsets in the context of acne pathogenesis will be of interest for future investigations.

### Cell-Type-Specific Changes in Gene Expression

**Figure 3:**
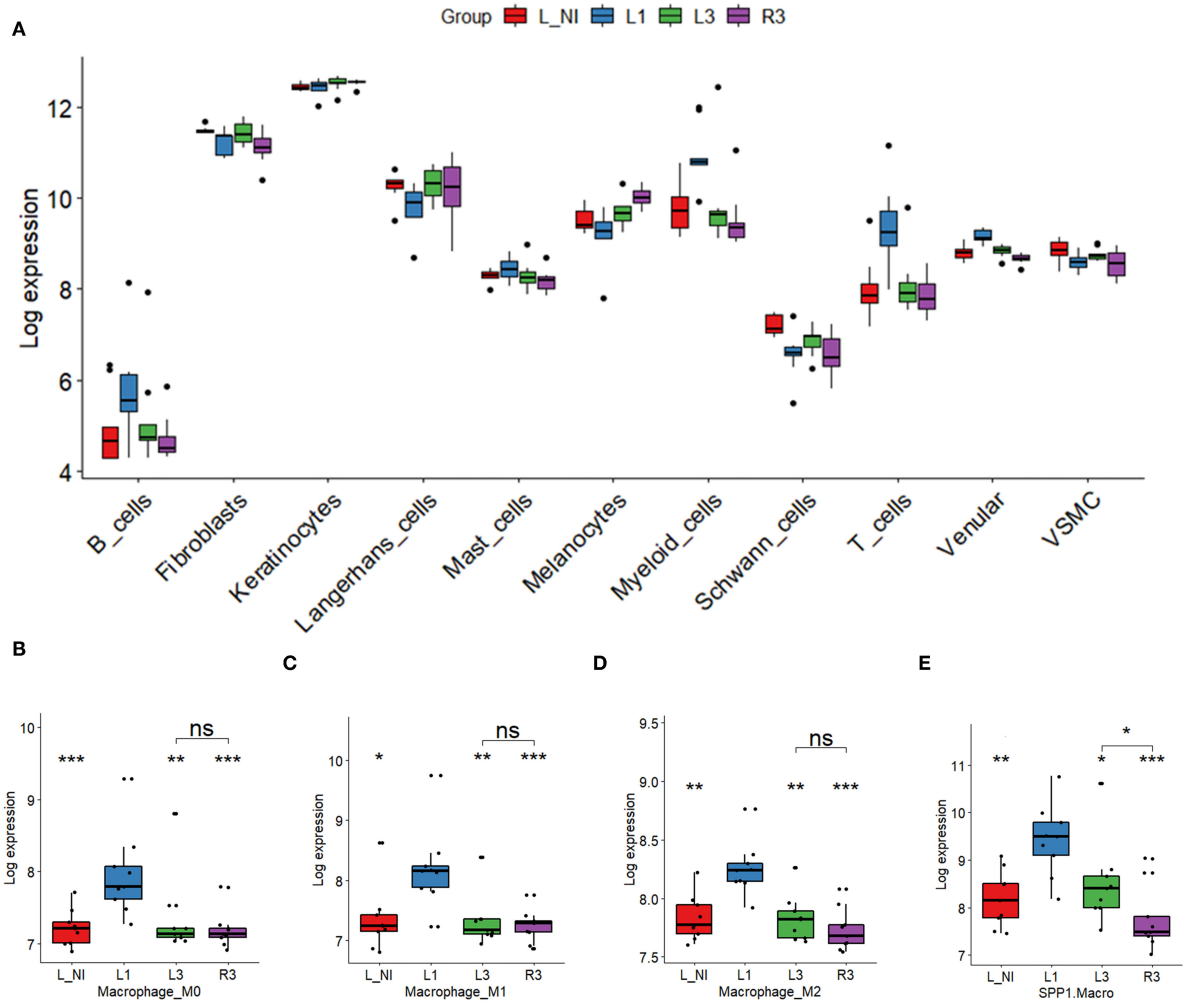
Cell-type-specific gene signatures distribution. **(A)** Overview of changes in expression for major cell types present in skin biopsies. **(B–E)** Changes in expression levels of marker genes associated with different classes of macrophages. *Represent the statistical significance of the difference in gene expression when comparing non-involved skin (L_NI), with acne papule (L1), spontaneously resolved papule (L3), or Trifarotene treated skin (R3). While expression of each class of macrophages is reduced when compared to papule signature, only SPP1 activated macrophages show a significant difference when compared to spontaneous resolution of acne papule. For each sample, an expression score was derived from selected marker genes for each cell type. Changes in expression in each clinical group were represented using boxplots. ns, non-significant; *p < 0.05; **p < 0.01; ***p < 0.0001.

To further investigate the effects of trifarotene on the various cell types present in skin biopsies, we obtained specific gene signatures through the re-analysis of publicly available single cell RNAseq data from healthy and acne skin biopsies [GSE150672, Hughes et al. (13)]. We identified 10 major cell types, as presented in the UMAP visualization (Supplementary Figure 2). The repartition of normal and acne cells was balanced in each group. From this analysis, we obtained marker genes for each cell type, and then generated a cell-type specific gene expression score in each of our study samples. The distribution of these scores is represented in the boxplots in Figure 3. There was a significant increase in immune cells, such as B cells, T cells, and myeloids/granulocytes in acne (L1) compared to non-involved skin. Upon resolution of the acne papule over time, with or without treatment with trifarotene, this over-expression of immune cells decreases. We also notice a significant reduction in expression of venular cell genes, and a significant increase in melanocytes, keratinocytes, and myeloid cells (Figure 3, Supplementary Figure 2).

### Changes in Macrophage Subsets

Cell type scoring analysis results showed that all subsets of macrophages, including the relatively newly identified SPP1/MERTK activated (SPP1^+^) macrophages, were increased in lesional skin and tended back toward normal levels with spontaneous lesion resolution or trifarotene-induced lesion resolution. As shown in Figure 3, M0, M1 and M2 macrophages decreased with papule resolution but were not significantly different in spontaneously resolved or trifarotene treated biopsies. However, trifarotene treatment significantly reduced frequencies of SPP1^+^ macrophages vs. spontaneously resolved acne papules (*P* = 0.040).

## Discussion

In a previously published article [Aubert et al. (14)], we showed that trifarotene-treated skin exhibited an expression profile expected from a retinoid, validating the bioactivity of trifarotene in the skin. Pathway analysis reported in that publication indicated that trifarotene treatment promoted epidermal differentiation and keratinization processes as expected for a retinoid (14). However, that analysis only compared the gene expression profiles *after* papules had resolved either spontaneously or after trifarotene treatment (14). The aim of the current transcriptomic analysis was to identify the gene expression signatures in acne papules as well as elucidate the pathways that are modulated *during* papule resolution. Specifically, we aimed to assess any trifarotene-specific effect on acne papule resolution. While a majority of the inflammation resolution patterns were similar in spontaneously resolved papules and trifarotene-treated lesions, trifarotene modulated a unique set of 67 genes with MMP12 and MMP13, CXCL13, XCL1, and SPP1/Osteopontin, being the most dominantly regulated. Pathways impacted by trifarotene included pro-inflammatory responses, neutrophil chemotaxis, and T cell migration.

While dysregulation of both the innate and adaptive immune responses stimulated by *C. acnes* is thought to have an essential role in acne pathogenesis, much remains to be learned about exact changes in cellular expression. Several groups have reported Th1/Th17 as driving adaptive immune responses in acne, with associated increases in B cells, macrophages, and cytokines (24–27). Retinoids and Vitamin A inhibit the Th17 pathway (24, 26). Recently, Yang et al. (28) used bioinformatic analysis on several datasets that included partial data (baseline only) from our dataset, to confirm the Th1/Th17 elevation in acne lesions compared with non-lesional skin of acne patients.

To determine whether expression changes of the 67 unique trifarotene-affected genes was associated with a change in cellular signature, we looked at markers associated with specific cell types. We found increased B cell and macrophage frequencies in acne lesions. Macrophages have both protective and pathogenic functions in skin and have classically been divided into subsets (M1 = pro-inflammatory and antimicrobial; M2 = anti-inflammatory and wound healing, and M0 = naïve macrophages) (29). While wound healing is driven by M2 macrophages, the newly described SPP1^+^ subset have been implicated in driving fibrosis, at least in the lungs (30). The increased frequencies of this latter population in acne papules suggest that these cells potentially drive fibrosis and production of MMPs, growth factors, and cytokines in acne lesions (29, 31). Further investigations could provide detailed insights into the role of SPP1^+^ macrophages in acne pathogenesis including scar formation.

Soluble osteopontin (OPN) is a multi-functional cytokine found elevated in psoriasis and other inflammatory diseases. It binds to integrins and CD44 on immune cells and plays a major role in cell adhesion, migration, and modulation of both Th1 and Th17 responses (32). OPN may have a role in acne via these effects (33).

The chemokine CXCL13 and its receptor, CXCR5, play a central role in driving humoral immunity during infection and vaccine responses (34, 35). Originally identified as a B-cell chemoattractant, CXCL13 exerts important functions in lymphoid neogenesis (23), and has been widely implicated in the pathogenesis of several autoimmune diseases and inflammatory conditions, as well as in lymphoproliferative disorders. In line with the CXCL13 expression data, our cellular analysis not only showed increased B cells in acne papules, but also that trifarotene treatment had a greater impact in reducing B cell frequencies vs. spontaneous resolution. B cells have been implicated in hidradenitis suppurativa, where they are thought to amplify inflammatory response (36). Similarly, in acne, increased numbers of B cells have been correlated with higher severity of disease (37). Gene expression and immunohistochemistry analyses showed a very similar immune response in 48-h-old papules in patients prone or not prone to scarring, characterized by elevated numbers of T cells, neutrophils and macrophages (38). However, the immune response only persisted in patients prone to scarring in 3-week-old papules, and was characterized by an important B-cell infiltrate (38). The exact role of B cells in acne pathogenesis remains unknown and the current analysis did not compare scarring vs. non-scarring patients, because our baseline evaluations did not reveal a clear signature for these groups. Future studies into skin B cells and CXCL13 expression may reveal new facets of acne pathogenesis.

MMPs are involved in tissue destruction and have a major role in scar formation and can mediate innate immune responses. In healthy skin, MMPs play an essential role in regulating the skin matrix. *C. acnes* induces production of various MMPs (39), and extracellular matrix remodeling regulated by MMPs is thought to be a part of the pathogenesis of acne (25, 40). The transcription factor activator protein-1 (AP-1), which regulates the expression of several MMPs has been shown to be upregulated in acne lesions (5). Targeting MMPs could be an interesting therapy in acne as this may be a potential way to minimize scar development and abnormal skin remodeling (39).

An increase in melanocytes occurred in both spontaneous and treated resolved acne lesions, and was greater in the trifarotene-treated samples. It is hard to determine whether this has any clinical correlate in melanin production given that retinoids have been shown to decrease pigmentation (14, 41, 42); the impact of trifarotene on acne scarring and hyperpigmentation are being evaluated in ongoing studies. In addition, there is a trend toward a decrease of venular cells following trifarotene treatment, which may indicate a decrease in cells of the vasculature or inflammatory cell recruitment. Much remains to be elucidated about the impact of trifarotene on acne lesions, tissue remodeling, and acne sequelae.

To our knowledge, this is the first clinical study comparing gene expression in normal skin, spontaneously resolving acne lesions, and topical-retinoid-treated acne lesions. Our results indicate that trifarotene, a retinoic acid receptor gamma (RARγ) selective agonist, regulates several unique genes and pathways but whether these are driven by RARγ vs. other RARs remains an intriguing question. While there is limited information on the role of the other retinoids in driving anti-inflammatory modules in acne, a cursory look suggests that the data is indeed controversial. Jalian et al. (43) reported that all-trans retinoic acid (ATRA) decreased expression of MMP9 in monocytes while isoretinoin treatment slightly increases OPN expression in acne patients (44). Future studies focusing on the mechanism of action of retinoids with non-selective and less selective actions on RAR pathways during acne treatment will help determine the impact of RAR isoforms on specific inflammatory responses.

## Data Availability Statement

The dataset presented in this study can be found in Gene Expression Omnibus under accession number GSE107232.

## Ethics Statement

The studies involving human participants were reviewed and approved by CHU Nantes. The patients/participants provided their written informed consent to participate in this study.

## Author Contributions

BD, SB-R, AK, and JKK were involved in study design and analysis. SB-R designed the computational framework and analyzed and interpreted the results. RC, VJ, and JKK aided in interpreting results and contributed to the writing of the manuscript. All authors discussed the results, read, and approved the final manuscript.

## Funding

Financial support for this study was provided by Galderma.

## Conflict of Interest

RC, VJ, and JKK are employees of Galderma; SB-R is an employee of Syneos Health and consultant for Galderma; BD and AK are investigators and consultants for Galderma.

## Publisher's Note

All claims expressed in this article are solely those of the authors and do not necessarily represent those of their affiliated organizations, or those of the publisher, the editors and the reviewers. Any product that may be evaluated in this article, or claim that may be made by its manufacturer, is not guaranteed or endorsed by the publisher.

## Supplementary Material

Supplementary Figure 1 Comparison of gene set enrichment analysis results obtained for the papule signature (L1 vs. LNS) and Trifarotene treatment signature (R3 vs. L1). **(A)** Heatmap of normalized enrichment score (NES) of Hallmark pathways significantly enriched in papule signature (FDR <0.05), and corresponding NES in the Trifarotene signature. **(B)** Heatmap of normalized enrichment score (NES) for a selection of Reactome pathways, with significant enrichment in both papule and trifarotene signature (FDR <0.05). Red: enrichment of up-regulated genes. Blue: enrichment of down-regulated genes.

Supplementary Figure 2 **(A)** Single cell data analysis of normal and acne skin biospies (GSE150672). UMAP representation of the dataset showing the main cell types detected. **(B)** Changes in expression levels of marker genes associated with different cell populations including Fibroblasts, melanocytes, venular cells, B cells, mast cells and myeloid cells. ns, non-significant; ^*^*p* < 0.05; ^**^*p* < 0.01; ^***^*p* < 0.0001.

Supplementary Table 1 Canonical pathway analysis of trifarotene treated papules. The table represent the canonical pathways with the highest enrichment *z*-score obtained from the papule signature and from trifarotene signature. All pathways show a reverse enrichment score in the trifarotene signature analysis.

Supplementary Table 2 Disease or Functions activation in trifarotene treated papules. This table represent a selection of disease or functions with the highest activation z-scores, obtained from the papule signature and from the trifarotene signature. All of them show a reverse activation z-score in the Trifarotene of signature analysis.

Supplementary Table 3 Summary of statistical results for the 67 genes specifically modulated by trifarotene. L-NI, non-involved skin; L1, papule at baseline; R3, papule after treatment with trifarotene.

Supplementary Table 4 Disease and functions found significantly modulated in an Ingenuity Pathways analysis of the 67 genes of interest. Activation *z*-scores provided in the table correspond to trifarotene gene expression profiles. Molecules listed correspond to genes from the 67 genes of interest mapping to the pathway or function of interest.

Supplementary Table 5 Summary of statistical results for the 69 genes specifically modulated in spontaneously resolved acne papule. L-NI, non-involved skin; L1, papule at baseline; L3, papule after treatment with vehicle.

Supplementary Table 6 Disease and functions analysis of the 69 genes specifically modulated in spontaneously resolved acne papules. This table represents a selection of disease or functions with the highest activation z-scores comparing papule signature (L1 vs L-NI) and vehicle signature (L3 vs L1) (FDR <0.05, *z*-score > 1.5). We can observe a reverse activation *z*-score between the expression levels in the papule (L1 vs. LNI) and after spontaneous resolution (L3 vs. L1).
