# Supplementary figures and images for: Transcriptomics Analysis Indicates Trifarotene Reverses Acne-Related Gene Expression Changes
Source: Front Med (Lausanne). 2021 Oct 22;8:745822. doi: 10.3389/fmed.2021.745822 (PMC8569320; doi:10.3389/fmed.2021.745822)

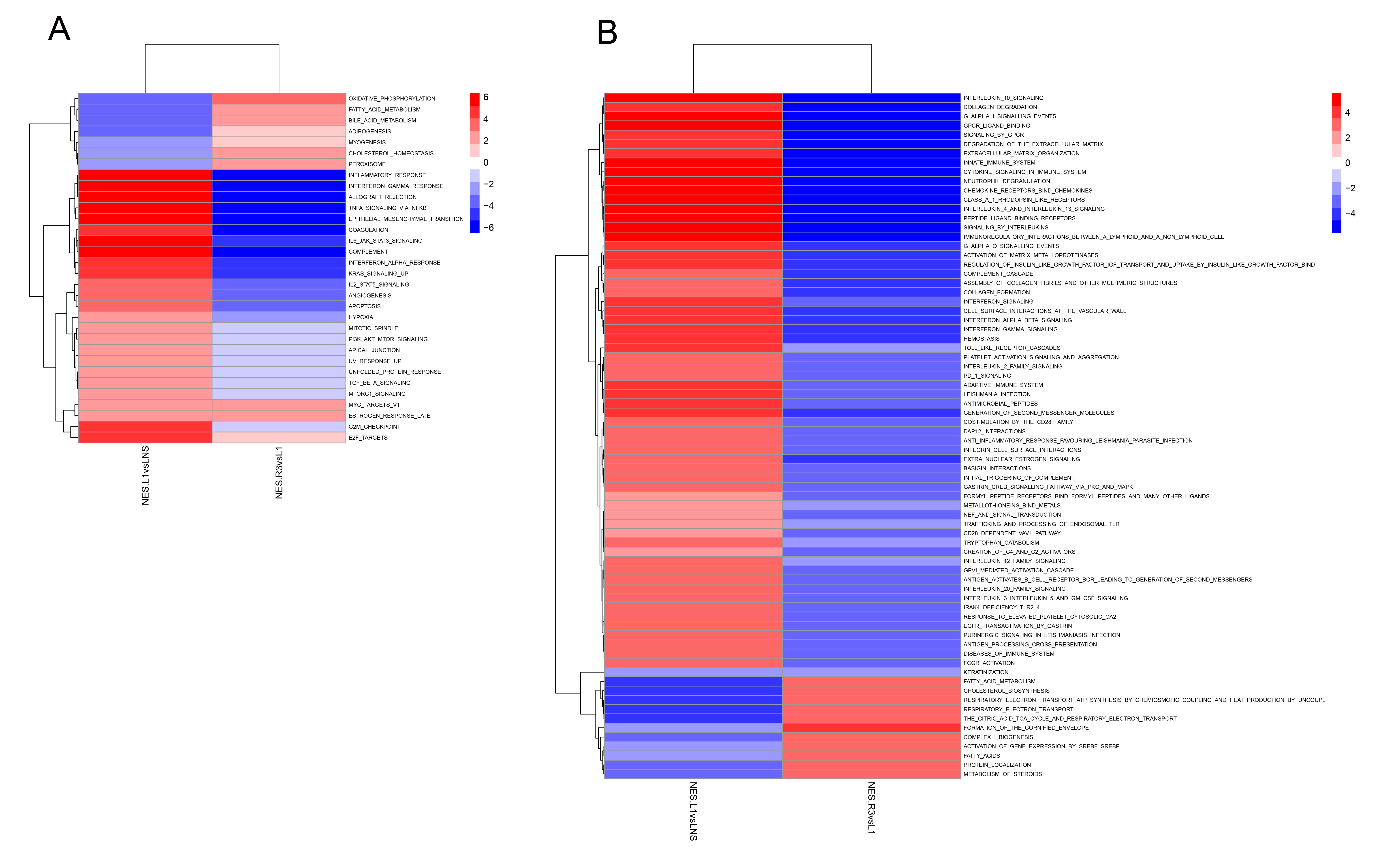

Supplement: Supplementary Figure 1 — Comparison of gene set enrichment analysis results obtained for the papule signature (L1 vs. LNS) and Trifarotene treatment signature (R3 vs. L1). (A) Heatmap of normalized enrichment score (NES) of Hallmark pathways significantly enriched in papule signature (FDR <0.05), and corresponding NES in the Trifarotene signature. (B) Heatmap of normalized enrichment score (NES) for a selection of Reactome pathways, with significant enrichment in both papule and trifarotene signature (FDR <0.05). Red: enrichment of up-regulated genes. Blue: enrichment of down-regulated genes. [file Image_1.JPEG]

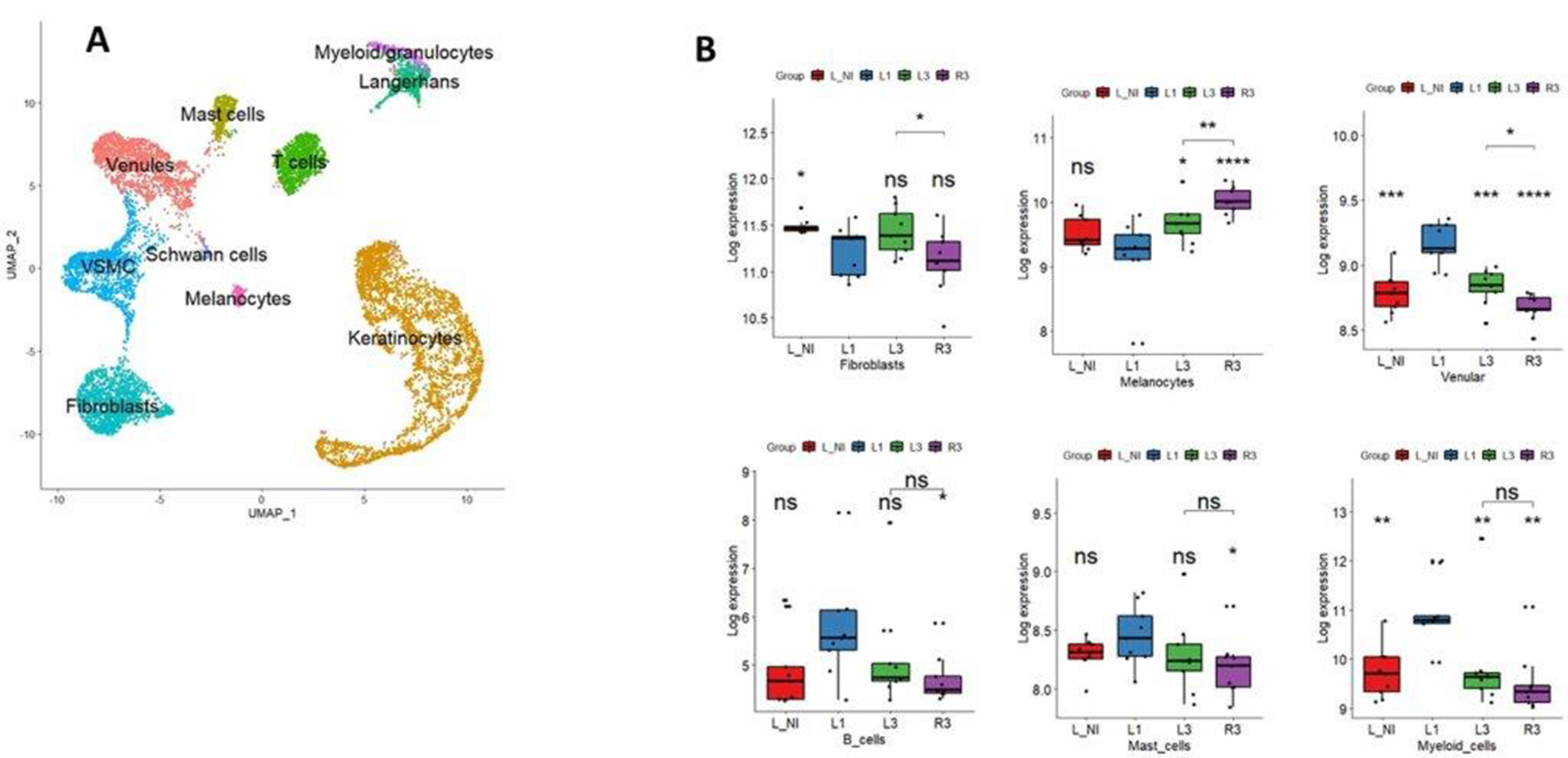

Supplement: Supplementary Figure 2 — (A) Single cell data analysis of normal and acne skin biospies (GSE150672). UMAP representation of the dataset showing the main cell types detected. (B) Changes in expression levels of marker genes associated with different cell populations including Fibroblasts, melanocytes, venular cells, B cells, mast cells and myeloid cells. ns, non-significant; *p < 0.05; **p < 0.01; ***p < 0.0001. [file Image_2.JPEG]
