# Supplementary material for: Transcriptomics Analysis Indicates Trifarotene Reverses Acne-Related Gene Expression Changes
Source: Front Med (Lausanne). 2021 Oct 22;8:745822. doi: 10.3389/fmed.2021.745822 (PMC8569320; doi:10.3389/fmed.2021.745822)
Supplement: Supplementary Table 1 — Canonical pathway analysis of trifarotene treated papules. The table represent the canonical pathways with the highest enrichment z-score obtained from the papule signature and from trifarotene signature. All pathways show a reverse enrichment score in the trifarotene signature analysis. [file Table_1.DOCX]

| **Ingenuity Canonical Pathways** | **z score Papule Profile** | **z score Trifarotene Profile** |
| --- | --- | --- |
| TREM1 Signaling | 4.123 | -4.796 |
| Crosstalk between Dendritic Cells and Natural Killer Cells | 4.123 | -4.123 |
| Neuroinflammation Signaling Pathway | 3.674 | -4.536 |
| Dendritic Cell Maturation | 3.441 | -4.899 |
| Role of NFAT in Regulation of the Immune Response | 3.357 | -3.771 |
| Osteoarthritis Pathway | 3.3 | -3.273 |
| Leukocyte Extravasation Signaling | 3.273 | -3.838 |
| Production of Nitric Oxide and Reactive Oxygen Species in Macrophages | 3.207 | -3.9 |
| Role of IL-17F in Allergic Inflammatory Airway Diseases | 3 | -2.53 |
| Natural Killer Cell Signaling | 2.985 | -3.545 |
| HOTAIR Regulatory Pathway | 2.673 | -2.324 |
| Role of Pattern Recognition Receptors in Recognition of Bacteria and Viruses | 2.53 | -3.873 |
| Acute Phase Response Signaling | 2.496 | -3.638 |
| HMGB1 Signaling | 2.309 | -3.606 |
| Fcγ Receptor-mediated Phagocytosis in Macrophages and Monocytes | 2.309 | -3.464 |
| Inflammasome pathway | 2.236 | -2.236 |
| T Cell Exhaustion Signaling Pathway | 2.138 | -2.324 |
| IL-17A Signaling in Airway Cells | 2.121 | -2.646 |
| Hepatic Fibrosis Signaling Pathway | 2.041 | -3.889 |
| Tryptophan Degradation to 2-amino-3-carboxymuconate Semialdehyde | 2 | -2 |
| NAD biosynthesis II (from tryptophan) | 2 | -2 |
| Th2 Pathway | 1.941 | -1.807 |
| Th1 Pathway | 1.897 | -2.84 |
| LPS/IL-1 Mediated Inhibition of RXR Function | 1.897 | -1.667 |
| IL-6 Signaling | 1.732 | -3.051 |
| Systemic Lupus Erythematosus In B Cell Signaling Pathway | 0.894 | -2.294 |
| Complement System | 0.816 | -1.897 |
| LXR/RXR Activation | -1.789 | 1.46 |
| Inhibition of Matrix Metalloproteases | -2.53 | 1.897 |
