# Supplementary material for: Transcriptomics Analysis Indicates Trifarotene Reverses Acne-Related Gene Expression Changes
Source: Front Med (Lausanne). 2021 Oct 22;8:745822. doi: 10.3389/fmed.2021.745822 (PMC8569320; doi:10.3389/fmed.2021.745822)
Supplement: Supplementary Table 2 — Disease or Functions activation in trifarotene treated papules. This table represent a selection of disease or functions with the highest activation z-scores, obtained from the papule signature and from the trifarotene signature. All of them show a reverse activation z-score in the Trifarotene of signature analysis. [file Table_2.DOCX]

| Diseases or Functions Annotation | Activation z.score Papule signature | Activation z.score Trifarotene signature |
| --- | --- | --- |
| Cell movement of leukocytes | 6.973 | -6.976 |
| Cell movement of blood cells | 7.051 | -7.244 |
| Leukocyte migration | 7.202 | -7.285 |
| Activation of cells | 5.611 | -5.508 |
| Cell movement of phagocytes | 6.695 | -7.151 |
| Quantity of leukocytes | 3.968 | -2.579 |
| Cell movement of myeloid cells | 6.714 | -6.93 |
| Activation of leukocytes | 6.041 | -5.289 |
| Proliferation of blood cells | 3.443 | -3.032 |
| Activation of blood cells | 6.281 | -5.514 |
| Quantity of blood cells | 4.309 | -3.022 |
| Proliferation of immune cells | 3.68 | -3.292 |
| Proliferation of mononuclear leukocytes | 3.626 | -3.177 |
| Proliferation of lymphatic system cells | 3.517 | -3.175 |
| Inflammatory response | 7.277 | -7.094 |
| Proliferation of lymphocytes | 3.759 | -3.195 |
| Inflammation of joint | 2.365 | -2.543 |
| Migration of cells | 8.059 | -8.277 |
| Homing of blood cells | 6.948 | -6.815 |
| Chemotaxis of blood cells | 6.991 | -6.617 |
| Homing of leukocytes | 6.948 | -6.814 |
| Binding of blood cells | 6.152 | -6.803 |
| Chemotaxis of leukocytes | 6.991 | -6.617 |
| Cell movement of mononuclear leukocytes | 6.32 | -5.911 |
| Homing of cells | 6.998 | -7.118 |
| Adhesion of blood cells | 6.16 | -6.776 |
| Binding of leukocytes | 6.318 | -6.96 |
| Adhesion of immune cells | 6.364 | -6.945 |
| Cell movement | 8.081 | -8.21 |
| Immune response of leukocytes | 4.414 | -5.255 |
| Chemotaxis | 6.962 | -6.846 |
| Cell movement of granulocytes | 6.538 | -6.539 |
| Chemotaxis of phagocytes | 7.045 | -6.633 |
| Activation of mononuclear leukocytes | 4.986 | -4.237 |
| Cell proliferation of T lymphocytes | 3.29 | -2.957 |
| Activation of lymphatic system cells | 4.83 | -3.911 |
| Chemotaxis of myeloid cells | 6.855 | -6.636 |
| Activation of lymphoid cells | 4.965 | -4.164 |
| Immune response of cells | 5.903 | -6.441 |
| Migration of phagocytes | 5.958 | -5.991 |
| Activation of lymphocytes | 4.894 | -4.094 |
| Degranulation of cells | 3.97 | -4.445 |
| Leukopoiesis | 5.624 | -6.492 |
| Cell movement of neutrophils | 5.985 | -6.35 |
| Migration of mononuclear leukocytes | 5.833 | -5.881 |
| Quantity of lymphatic system cells | 4.256 | -3.034 |
| Cell death of immune cells | 3.177 | -3.66 |
| Cellular infiltration | 4.336 | -3.988 |
| Cell death of blood cells | 2.99 | -3.613 |
| Quantity of mononuclear leukocytes | 3.579 | -2.303 |
| Cell movement of lymphocytes | 6.089 | -5.782 |
| Quantity of lymphoid cells | 3.96 | -2.667 |
| Cellular infiltration by blood cells | 3.962 | -3.72 |
| Lymphocyte migration | 5.942 | -5.932 |
| Cellular infiltration by leukocytes | 4.097 | -3.651 |
| Quantity of lymphocytes | 3.889 | -2.575 |
| Quantity of cells | 4.111 | -3.42 |
| Response of mononuclear leukocytes | 3.661 | -3.434 |
| Cell movement of antigen presenting cells | 5.242 | -5.43 |
| Accumulation of leukocytes | 3.95 | -2.502 |
| Accumulation of blood cells | 4.062 | -2.636 |
| Recruitment of leukocytes | 5.587 | -5.614 |
| Chemotaxis of granulocytes | 6.064 | -5.77 |
| Cellular homeostasis | 6.19 | -6.146 |
| Accumulation of cells | 4.004 | -2.921 |
| Degranulation of phagocytes | 3.067 | -3.602 |
| Chemotaxis of neutrophils | 5.803 | -5.582 |
| Recruitment of cells | 6.026 | -6.218 |
| Activation of phagocytes | 4.865 | -5.069 |
| Recruitment of granulocytes | 4.806 | -5.383 |
| Differentiation of mononuclear leukocytes | 5.484 | -6.486 |
| Hematopoiesis of mononuclear leukocytes | 5.407 | -6.419 |
| Recruitment of myeloid cells | 5.289 | -5.531 |
| Recruitment of neutrophils | 4.536 | -5.008 |
| Response of myeloid cells | 4.106 | -5.29 |
| Hypersensitive reaction | 3.178 | -4.225 |
| Mobilization of Ca2+ | 6.087 | -5.201 |
| Activation of T lymphocytes | 4.83 | -3.731 |
| T cell migration | 5.544 | -4.784 |
| Recruitment of phagocytes | 5.152 | -5.413 |
| Response of phagocytes | 4.614 | -5.241 |
| Cell movement of T lymphocytes | 5.641 | -4.703 |
| Binding of phagocytes | 4.261 | -5.097 |
| Quantity of T lymphocytes | 5.22 | -3.737 |
| Quantity of antigen presenting cells | 3.019 | -2.794 |
| Interaction of mononuclear leukocytes | 5.96 | -6.297 |
| Homeostasis of leukocytes | 5.965 | -6.507 |
| Binding of lymphatic system cells | 5.612 | -5.555 |
| Degranulation of granulocytes | 2.891 | -3.712 |
| Cellular infiltration by myeloid cells | 4.363 | -4.698 |
| Binding of mononuclear leukocytes | 5.811 | -6.006 |
| Interaction of lymphocytes | 5.616 | -5.723 |
| Cellular infiltration by granulocytes | 4.169 | -4.042 |
| Binding of professional phagocytic cells | 4.179 | -5.026 |
| Binding of lymphocytes | 5.536 | -5.477 |
| Lymphopoiesis | 5.553 | -6.509 |
| Lymphocyte homeostasis | 5.881 | -6.304 |
| Homing of mononuclear leukocytes | 5.902 | -5.38 |
| Response of lymphatic system cells | 3.435 | -3.081 |
| Binding of myeloid cells | 3.954 | -4.839 |
| Migration of antigen presenting cells | 4.783 | -4.761 |
| Binding of tumor cell lines | 3.35 | -3.726 |
| Degranulation of neutrophils | 2.433 | -2.804 |
| Advanced malignant tumor | 3.475 | -3.446 |
| Flux of Ca2+ | 3.814 | -2.838 |
| T cell homeostasis | 5.912 | -6.426 |
| Activation of myeloid cells | 4.969 | -5.181 |
| Immune response of phagocytes | 3.831 | -4.658 |
| Migration of myeloid cells | 4.858 | -5.173 |
| Cell movement of dendritic cells | 4.797 | -4.74 |
| T cell development | 5.908 | -6.426 |
| Ion homeostasis of cells | 4.023 | -3.092 |
| Interaction of T lymphocytes | 5.521 | -5.446 |
| Response of lymphocytes | 3.497 | -3.224 |
| Binding of T lymphocytes | 5.459 | -5.203 |
| Adhesion of lymphocytes | 5.47 | -5.487 |
| Cellular infiltration by phagocytes | 4.08 | -4.709 |
| Adhesion of mononuclear leukocytes | 5.738 | -5.99 |
| Chemotaxis of mononuclear leukocytes | 5.623 | -4.929 |
| Cell movement of macrophages | 4.34 | -4.583 |
| Transmigration of cells | 3.504 | -3.498 |
| Stimulation of cells | 5.298 | -5.199 |
| Infiltration by neutrophils | 3.464 | -3.811 |
| Cell-mediated response | 2.328 | -2.605 |
| Cell movement of monocytes | 4.459 | -4.254 |
| Synthesis of reactive oxygen species | 4.329 | -5.497 |
| Binding of granulocytes | 3.272 | -3.934 |
| Adhesion of phagocytes | 3.175 | -3.816 |
| Metabolism of reactive oxygen species | 4.447 | -5.605 |
| Response of granulocytes | 2.003 | -3.203 |
| Adhesion of T lymphocytes | 5.275 | -5.198 |
| Adhesion of granulocytes | 2.841 | -3.499 |
