# Supplementary material for: Transcriptomics Analysis Indicates Trifarotene Reverses Acne-Related Gene Expression Changes
Source: Front Med (Lausanne). 2021 Oct 22;8:745822. doi: 10.3389/fmed.2021.745822 (PMC8569320; doi:10.3389/fmed.2021.745822)
Supplement: Supplementary Table 3 — Summary of statistical results for the 67 genes specifically modulated by trifarotene. L-NI, non-involved skin; L1, papule at baseline; R3, papule after treatment with trifarotene. [file Table_3.DOCX]

| Symbol | Entrez Gene Name | Affymetrix | Fold Change L1 vs L-NI | Fold Change R3 vs L1 |
| --- | --- | --- | --- | --- |
| ADAM12 | ADAM metallopeptidase domain 12 | 226777_at | 3.50 | -3.84 |
| ADAMDEC1 | ADAM like decysin 1 | 206134_at | 6.34 | -6.29 |
| APELA | apelin receptor early endogenous ligand | 1559280_a_at | 2.03 | -2.70 |
| ARHGAP9 | Rho GTPase activating protein 9 | 224451_x_at | 2.10 | -2.14 |
| BCAT1 | branched chain amino acid transaminase 1 | 214452_at | 3.05 | -3.66 |
| BIRC3 | baculoviral IAP repeat containing 3 | 210538_s_at | 2.11 | -2.23 |
| CBLN2 | cerebellin 2 precursor | 242301_at | 2.83 | -2.61 |
| CCDC71L | coiled-coil domain containing 71 like | 229521_at | 2.01 | -2.26 |
| CCNA1 | cyclin A1 | 205899_at | 4.39 | -3.55 |
| CD37 | CD37 molecule | 204192_at | 2.13 | -2.13 |
| CD69 | CD69 molecule | 209795_at | 2.07 | -2.10 |
| CD80 | CD80 molecule | 1554519_at | 2.69 | -4.03 |
| CD84 | CD84 molecule | 230391_at | 2.12 | -2.57 |
| CLEC4A | C-type lectin domain family 4 member A | 221724_s_at | 2.08 | -2.32 |
| CTLA4 | cytotoxic T-lymphocyte associated protein 4 | 236341_at | 2.74 | -2.54 |
| CXCL13 | C-X-C motif chemokine ligand 13 | 205242_at | 20.49 | -23.54 |
| CYTH4 | cytohesin 4 | 219183_s_at | 2.21 | -2.36 |
| DMXL2 | Dmx like 2 | 215761_at | 2.32 | -2.35 |
| EVI2B | ecotropic viral integration site 2B | 211742_s_at | 2.26 | -2.35 |
| FERMT3 | fermitin family member 3 | 223303_at | 2.23 | -2.12 |
| FNDC3B | fibronectin type III domain containing 3B | 229865_at | 2.12 | -2.35 |
| GABRA4 | gamma-aminobutyric acid type A receptor subunit alpha4 | 208463_at | -2.58 | 2.26 |
| GASK1B | golgi associated kinase 1B | 219872_at | 2.05 | -2.30 |
| GLIPR1 | GLI pathogenesis related 1 | 204222_s_at | 2.11 | -2.48 |
| GLIPR2 | GLI pathogenesis related 2 | 225604_s_at | 2.29 | -2.24 |
| GLIS3 | GLIS family zinc finger 3 | 229435_at | 2.34 | -3.07 |
| GPRIN3 | GPRIN family member 3 | 1556698_a_at | 2.15 | -2.27 |
| HLA-DPA1 | major histocompatibility complex, class II, DP alpha 1 | 213537_at | 2.19 | -2.14 |
| HP | haptoglobin | 206697_s_at | 3.00 | -2.99 |
| IL18BP | interleukin 18 binding protein | 222868_s_at | 2.09 | -2.37 |
| IL2RG | interleukin 2 receptor subunit gamma | 204116_at | 2.17 | -2.28 |
| INA | internexin neuronal intermediate filament protein alpha | 204465_s_at | 2.12 | 2.34 |
| IRF8 | interferon regulatory factor 8 | 204057_at | 2.22 | -2.02 |
| ITGAX | integrin subunit alpha X | 210184_at | 3.22 | -2.85 |
| KLHL6 | kelch like family member 6 | 1555275_a_at | 2.70 | -3.05 |
| LINC01224 | -- | 233142_at | -2.05 | 3.51 |
| LOXL2 | lysyl oxidase like 2 | 202998_s_at | 2.16 | -2.18 |
| LPXN | leupaxin | 216250_s_at | 2.03 | -2.25 |
| LY86 | lymphocyte antigen 86 | 205859_at | 2.10 | -2.18 |
| MMP12 | matrix metallopeptidase 12 | 204580_at | 19.41 | -11.13 |
| MMP13 | matrix metallopeptidase 13 | 205959_at | 4.86 | -4.30 |
| MMP19 | matrix metallopeptidase 19 | 204575_s_at | 2.24 | -2.71 |
| MSR1 | macrophage scavenger receptor 1 | 214770_at | 2.18 | -2.45 |
| NCEH1 | neutral cholesterol ester hydrolase 1 | 225847_at | 2.41 | -2.70 |
| NRP2 | neuropilin 2 | 223510_at | 2.25 | -2.46 |
| P2RY8 | P2Y receptor family member 8 | 229686_at | 2.20 | -2.23 |
| P2RY10 | P2Y receptor family member 10 | 236280_at | 2.16 | -2.05 |
| PAPSS2 | 3'-phosphoadenosine 5'-phosphosulfate synthase 2 | 203060_s_at | 2.21 | -2.60 |
| PARVG | parvin gamma | 223562_at | 2.16 | -2.23 |
| PTHLH | parathyroid hormone like hormone | 211756_at | 2.13 | -2.55 |
| PTPN22 | protein tyrosine phosphatase non-receptor type 22 | 236539_at | 2.01 | -2.27 |
| PTPRO | protein tyrosine phosphatase receptor type O | 208121_s_at | 2.12 | -2.26 |
| PXMP4 | peroxisomal membrane protein 4 | 238746_at | -2.19 | 2.14 |
| RAC2 | Rac family small GTPase 2 | 207419_s_at | 2.13 | -2.03 |
| RHOH | ras homolog family member H | 204951_at | 2.04 | -2.57 |
| SELP | selectin P | 206049_at | 2.42 | -2.10 |
| SELPLG | selectin P ligand | 209879_at | 2.01 | -2.06 |
| SH2B3 | SH2B adaptor protein 3 | 203320_at | 2.07 | -2.26 |
| SIRPB1 | signal regulatory protein beta 1 | 206934_at | 2.51 | -2.45 |
| SLAMF7 | SLAM family member 7 | 219159_s_at | 2.64 | -2.49 |
| SLC6A14 | solute carrier family 6 member 14 | 219795_at | 2.10 | -2.04 |
| SNAP25 | synaptosome associated protein 25 | 202508_s_at | 2.23 | -2.40 |
| SPP1 | secreted phosphoprotein 1 | 1568574_x_at | 12.63 | -28.23 |
| TRG-AS1 | T cell receptor gamma locus antisense RNA 1 | 239237_at | 2.05 | -2.33 |
| TYROBP | transmembrane immune signaling adaptor TYROBP | 204122_at | 2.06 | -2.39 |
| VCAN | versican | 215646_s_at | 2.02 | -2.43 |
| XCL1 | X-C motif chemokine ligand 1 | 206366_x_at | 2.10 | -2.02 |
