# Supplementary material for: Transcriptomics Analysis Indicates Trifarotene Reverses Acne-Related Gene Expression Changes
Source: Front Med (Lausanne). 2021 Oct 22;8:745822. doi: 10.3389/fmed.2021.745822 (PMC8569320; doi:10.3389/fmed.2021.745822)
Supplement: Supplementary Table 4 — Disease and functions found significantly modulated in an Ingenuity Pathways analysis of the 67 genes of interest. Activation z-scores provided in the table correspond to trifarotene gene expression profiles. Molecules listed correspond to genes from the 67 genes of interest mapping to the pathway or function of interest. [file Table_4.DOCX]

| Categories | Diseases or Functions Annotation | p-value | Predicted Activation State | Activation z-score | Molecules | # Molecules |
| --- | --- | --- | --- | --- | --- | --- |
| Cell-To-Cell Signaling and Interaction | Binding of blood cells | 6.86E-13 | Decreased | -3.74 | CD37,CD69,CD80,CD84,CTLA4,CXCL13,FERMT3,IRF8,ITGAX,MSR1,P2RY8,PTPN22,RAC2,SELP,SELPLG,SPP1,TYROBP | 17 |
| Cell-To-Cell Signaling and Interaction,Hematological System Development and Function | Binding of leukocytes | 2.04E-11 | Decreased | -3.649 | CD37,CD69,CD80,CTLA4,CXCL13,FERMT3,ITGAX,MSR1,P2RY8,PTPN22,RAC2,SELP,SELPLG,SPP1,TYROBP | 15 |
| Cellular Movement | Migration of cells | 2.41E-09 | Decreased | -3.559 | ADAM12,BCAT1,CD37,CD69,CD80,CD84,CTLA4,CXCL13,FERMT3,FNDC3B,GLIPR2,HP,IL18BP,ITGAX,LOXL2,MMP12,MMP13,MMP19,MSR1,NRP2,PTHLH,PTPRO,RAC2,SELP,SELPLG,SH2B3,SPP1,TYROBP,VCAN,XCL1 | 30 |
| Cellular Movement | Cell movement | 3.35E-10 | Decreased | -3.549 | ADAM12,BCAT1,CD37,CD69,CD80,CD84,CTLA4,CXCL13,FERMT3,FNDC3B,GLIPR2,HP,IL18BP,IRF8,ITGAX,LOXL2,LPXN,MMP12,MMP13,MMP19,MSR1,NRP2,PARVG,PTHLH,PTPRO,RAC2,SELP,SELPLG,SH2B3,SPP1,TYROBP,VCAN,XCL1 | 33 |
| Cell-To-Cell Signaling and Interaction,Hematological System Development and Function,Immune Cell Trafficking | Adhesion of immune cells | 8.34E-11 | Decreased | -3.527 | CD37,CD69,CD80,CXCL13,FERMT3,ITGAX,MSR1,P2RY8,PTPN22,RAC2,SELP,SELPLG,SPP1,TYROBP | 14 |
| Cellular Movement,Hematological System Development and Function,Immune Cell Trafficking,Inflammatory Response | Cell movement of phagocytes | 2.82E-11 | Decreased | -3.374 | CD37,CD69,CD80,CXCL13,FERMT3,HP,ITGAX,MMP12,NRP2,PTPRO,RAC2,SELP,SELPLG,SH2B3,SPP1,TYROBP,VCAN,XCL1 | 18 |
| Cellular Movement,Immune Cell Trafficking | Leukocyte migration | 9.47E-11 | Decreased | -3.121 | CD37,CD69,CD80,CD84,CTLA4,CXCL13,FERMT3,HP,IL18BP,ITGAX,MMP12,MSR1,NRP2,PTPRO,RAC2,SELP,SELPLG,SH2B3,SPP1,TYROBP,VCAN,XCL1 | 22 |
| Cellular Function and Maintenance | Engulfment of cells | 1.18E-06 | Decreased | -3.116 | FERMT3,HP,IL2RG,IRF8,ITGAX,MSR1,RAC2,SELPLG,SIRPB1,SLAMF7,SNAP25,TYROBP | 12 |
| Cellular Movement,Hematological System Development and Function,Immune Cell Trafficking,Inflammatory Response | Chemotaxis of leukocytes | 1.79E-07 | Decreased | -3.019 | CD37,CD69,CXCL13,HP,NRP2,PTPRO,RAC2,SELP,SELPLG,SPP1,XCL1 | 11 |
| Cellular Movement,Hematological System Development and Function,Immune Cell Trafficking | Cell movement of leukocytes | 1.86E-11 | Decreased | -2.985 | CD37,CD69,CD80,CTLA4,CXCL13,FERMT3,HP,IL18BP,ITGAX,MMP12,MSR1,NRP2,PTPRO,RAC2,SELP,SELPLG,SH2B3,SPP1,TYROBP,VCAN,XCL1 | 21 |
| Cell-To-Cell Signaling and Interaction,Cellular Function and Maintenance,Inflammatory Response | Phagocytosis of cells | 1.53E-06 | Decreased | -2.96 | FERMT3,IL2RG,IRF8,ITGAX,MSR1,RAC2,SELPLG,SIRPB1,SLAMF7,TYROBP | 10 |
| Cellular Movement | Cell movement of tumor cell lines | 1.23E-05 | Decreased | -2.919 | ADAM12,CD69,CXCL13,FERMT3,MMP12,MMP19,NRP2,PARVG,PTHLH,RAC2,SELP,SELPLG,SH2B3,SPP1,VCAN,XCL1 | 16 |
| Cellular Movement,Hematological System Development and Function,Immune Cell Trafficking,Inflammatory Response | Cell movement of neutrophils | 4.5E-06 | Decreased | -2.914 | CD37,CD69,FERMT3,MMP12,RAC2,SELP,SELPLG,SPP1,XCL1 | 9 |
| Cellular Movement,Hematological System Development and Function,Immune Cell Trafficking,Inflammatory Response | Chemotaxis of phagocytes | 2.55E-07 | Decreased | -2.906 | CD37,CD69,HP,NRP2,PTPRO,RAC2,SELP,SELPLG,SPP1,XCL1 | 10 |
| Inflammatory Response | Inflammatory response | 7.09E-09 | Decreased | -2.903 | BIRC3,CD37,CD69,CD84,CLEC4A,CXCL13,HP,LOXL2,MMP19,MSR1,NRP2,PTPRO,RAC2,SELP,SELPLG,SPP1,TYROBP,XCL1 | 18 |
| Cellular Movement | Cell movement of myeloid cells | 2.06E-09 | Decreased | -2.855 | CD37,CD69,FERMT3,HP,ITGAX,MMP12,MSR1,PTPRO,RAC2,SELP,SELPLG,SH2B3,SPP1,TYROBP,VCAN,XCL1 | 16 |
| Cellular Function and Maintenance | Endocytosis | 0.000202 | Decreased | -2.789 | FERMT3,HP,IRF8,MSR1,RAC2,SELPLG,SIRPB1,SLAMF7,SNAP25,TYROBP | 10 |
| Cellular Movement | Migration of tumor cell lines | 0.00183 | Decreased | -2.763 | ADAM12,CXCL13,MMP12,MMP19,NRP2,PTHLH,RAC2,SH2B3,SPP1,VCAN,XCL1 | 11 |
| Cell-To-Cell Signaling and Interaction,Hematological System Development and Function | Binding of mononuclear leukocytes | 2.58E-09 | Decreased | -2.742 | CD69,CD80,CTLA4,CXCL13,FERMT3,ITGAX,RAC2,SELP,SELPLG,SPP1 | 10 |
| Cellular Movement,Hematological System Development and Function,Immune Cell Trafficking | Cell movement of mononuclear leukocytes | 1.21E-09 | Decreased | -2.733 | CD69,CD80,CTLA4,CXCL13,FERMT3,HP,IL18BP,ITGAX,MMP12,PTPRO,RAC2,SELP,SELPLG,SPP1,XCL1 | 15 |
| Cellular Movement | Chemotaxis of myeloid cells | 1.88E-06 | Decreased | -2.731 | CD37,CD69,HP,PTPRO,RAC2,SELP,SELPLG,SPP1,XCL1 | 9 |
| Cell-To-Cell Signaling and Interaction | Binding of myeloid cells | 7.67E-08 | Decreased | -2.695 | CD37,CD69,FERMT3,ITGAX,MSR1,PTPN22,RAC2,SELP,SELPLG | 9 |
| Cell-To-Cell Signaling and Interaction,Hematological System Development and Function,Inflammatory Response | Binding of professional phagocytic cells | 4.4E-08 | Decreased | -2.692 | CD37,CD69,FERMT3,ITGAX,MSR1,PTPN22,RAC2,SELP,SELPLG | 9 |
| Cell-To-Cell Signaling and Interaction,Cellular Function and Maintenance,Inflammatory Response | Phagocytosis of blood cells | 2.03E-07 | Decreased | -2.646 | FERMT3,IRF8,ITGAX,MSR1,RAC2,SELPLG,SIRPB1,SLAMF7,TYROBP | 9 |
| Cellular Movement,Hematological System Development and Function,Immune Cell Trafficking | Cell movement of granulocytes | 3.52E-06 | Decreased | -2.632 | CD37,CD69,FERMT3,MMP12,MSR1,RAC2,SELP,SELPLG,SPP1,XCL1 | 10 |
| Cellular Movement,Hematological System Development and Function,Immune Cell Trafficking,Inflammatory Response | Migration of phagocytes | 1.02E-09 | Decreased | -2.62 | CD80,CXCL13,ITGAX,MMP12,NRP2,RAC2,SELP,SELPLG,SH2B3,SPP1,TYROBP,VCAN | 12 |
| Cellular Function and Maintenance | Internalization of cells | 1.98E-05 | Decreased | -2.611 | FERMT3,IRF8,MSR1,RAC2,SELPLG,SIRPB1,SLAMF7,TYROBP | 8 |
| Cellular Function and Maintenance | Endocytosis by eukaryotic cells | 0.000138 | Decreased | -2.607 | FERMT3,HP,IRF8,MSR1,SELPLG,SIRPB1,SLAMF7,TYROBP | 8 |
| Cell-To-Cell Signaling and Interaction,Hematological System Development and Function,Immune Cell Trafficking | Adhesion of mononuclear leukocytes | 4.78E-08 | Decreased | -2.575 | CD80,CXCL13,FERMT3,ITGAX,RAC2,SELP,SELPLG,SPP1 | 8 |
| Cell-To-Cell Signaling and Interaction,Hematological System Development and Function | Binding of lymphocytes | 7.19E-09 | Decreased | -2.567 | CD69,CD80,CTLA4,CXCL13,FERMT3,RAC2,SELP,SELPLG,SPP1 | 9 |
| Cellular Movement,Hematological System Development and Function,Immune Cell Trafficking,Inflammatory Response | Chemotaxis of neutrophils | 1.79E-06 | Decreased | -2.555 | CD37,CD69,RAC2,SELP,SELPLG,SPP1,XCL1 | 7 |
| Cellular Movement,Hematological System Development and Function,Immune Cell Trafficking | Migration of mononuclear leukocytes | 4.71E-08 | Decreased | -2.478 | CD69,CD80,CTLA4,CXCL13,FERMT3,IL18BP,ITGAX,RAC2,SELP,SELPLG,SPP1,XCL1 | 12 |
| Cellular Movement,Hematological System Development and Function,Immune Cell Trafficking | Lymphocyte migration | 2.01E-07 | Decreased | -2.477 | CD69,CD80,CTLA4,CXCL13,FERMT3,IL18BP,RAC2,SELP,SELPLG,SPP1,XCL1 | 11 |
| Cancer,Organismal Injury and Abnormalities | Advanced stage tumor | 5.85E-08 | Decreased | -2.455 | ADAM12,BCAT1,CCNA1,CD80,CTLA4,FERMT3,GABRA4,IL18BP,IL2RG,IRF8,KLHL6,LOXL2,NRP2,P2RY8,PAPSS2,RAC2,SELP,SH2B3,SPP1,VCAN | 20 |
| Cancer,Organismal Injury and Abnormalities | Advanced malignant tumor | 2.95E-07 | Decreased | -2.455 | ADAM12,BCAT1,CCNA1,CD80,CTLA4,FERMT3,GABRA4,IL18BP,IL2RG,IRF8,KLHL6,LOXL2,NRP2,P2RY8,PAPSS2,RAC2,SELP,SPP1,VCAN | 19 |
| Cancer,Organismal Injury and Abnormalities,Tumor Morphology | Progression of tumor | 0.0015 | Decreased | -2.39 | ADAM12,CD80,CTLA4,LOXL2,NRP2,SPP1 | 6 |
| Cell-To-Cell Signaling and Interaction,Hematological System Development and Function,Immune Cell Trafficking | Adhesion of lymphocytes | 2E-07 | Decreased | -2.387 | CD80,CXCL13,FERMT3,RAC2,SELP,SELPLG,SPP1 | 7 |
| Cardiovascular System Development and Function,Cell-To-Cell Signaling and Interaction | Binding of endothelial cells | 8.22E-05 | Decreased | -2.376 | CD37,CTLA4,FERMT3,SELP,SELPLG,SPP1 | 6 |
| Cell-To-Cell Signaling and Interaction | Adhesion of myeloid cells | 1.62E-06 | Decreased | -2.371 | CD37,FERMT3,ITGAX,PTPN22,RAC2,SELP,SELPLG | 7 |
| Cellular Movement,Hematological System Development and Function,Immune Cell Trafficking | Homing of mononuclear leukocytes | 1.45E-05 | Decreased | -2.37 | CXCL13,HP,PTPRO,RAC2,SELPLG,SPP1,XCL1 | 7 |
| Cardiovascular System Development and Function | Development of vasculature | 7.14E-05 | Decreased | -2.365 | ADAM12,APELA,FERMT3,HP,IL18BP,ITGAX,LOXL2,MMP12,MMP13,NRP2,PTHLH,RAC2,SELP,SNAP25,SPP1 | 15 |
| Cardiovascular System Development and Function,Organismal Development | Angiogenesis | 7.28E-05 | Decreased | -2.365 | ADAM12,APELA,FERMT3,HP,IL18BP,ITGAX,LOXL2,MMP12,MMP13,NRP2,PTHLH,RAC2,SELP,SPP1 | 14 |
| Cancer,Organismal Injury and Abnormalities | Secondary tumor | 0.000155 | Decreased | -2.283 | ADAM12,CCNA1,CD80,CTLA4,FERMT3,IL18BP,IL2RG,LOXL2,NRP2,PAPSS2,RAC2,SELP,SPP1,VCAN | 14 |
| Cellular Function and Maintenance,Hematological System Development and Function | Engulfment of myeloid cells | 5.07E-05 | Decreased | -2.236 | IRF8,MSR1,SELPLG,SIRPB1,SLAMF7,TYROBP | 6 |
| Cell-To-Cell Signaling and Interaction,Hematological System Development and Function | Binding of T lymphocytes | 4.41E-07 | Decreased | -2.219 | CD69,CD80,CTLA4,RAC2,SELP,SELPLG,SPP1 | 7 |
| Cellular Development,Cellular Growth and Proliferation,Hematological System Development and Function,Hematopoiesis,Lymphoid Tissue Structure and Development,Tissue Development | Development of mononuclear leukocytes | 1.69E-06 | Decreased | -2.218 | CD69,CD80,CTLA4,IL2RG,IRF8,KLHL6,MMP19,MSR1,PTHLH,PTPN22,RHOH,SH2B3,SPP1,TYROBP | 14 |
| Cellular Movement,Hematological System Development and Function,Immune Cell Trafficking | Migration of antigen presenting cells | 2.02E-08 | Decreased | -2.213 | CD80,CXCL13,MMP12,NRP2,RAC2,SH2B3,SPP1,TYROBP,VCAN | 9 |
| Cell-mediated Immune Response,Cellular Movement,Hematological System Development and Function,Immune Cell Trafficking | Cell movement of T lymphocytes | 3.52E-08 | Decreased | -2.213 | CD69,CD80,CTLA4,CXCL13,IL18BP,RAC2,SELP,SELPLG,SPP1,XCL1 | 10 |
| Cellular Movement,Hematological System Development and Function,Immune Cell Trafficking,Inflammatory Response | Infiltration by neutrophils | 0.000578 | Decreased | -2.184 | MMP12,RAC2,SELP,SPP1,XCL1 | 5 |
| Cell-mediated Immune Response,Cellular Movement,Hematological System Development and Function,Immune Cell Trafficking,Lymphoid Tissue Structure and Development | Homing of T lymphocytes | 5.09E-05 | Decreased | -2.183 | CXCL13,RAC2,SELPLG,SPP1,XCL1 | 5 |
| Cardiovascular System Development and Function,Cell-To-Cell Signaling and Interaction | Adhesion of endothelial cells | 0.000189 | Decreased | -2.175 | CD37,FERMT3,SELP,SELPLG,SPP1 | 5 |
| Cell-To-Cell Signaling and Interaction,Hematological System Development and Function,Immune Cell Trafficking,Inflammatory Response | Adhesion of phagocytes | 8.26E-07 | Decreased | -2.171 | CD37,FERMT3,ITGAX,PTPN22,RAC2,SELP,SELPLG | 7 |
| Hematological System Development and Function,Lymphoid Tissue Structure and Development,Tissue Morphology | Quantity of CD4+ T-lymphocytes | 2.02E-08 | Decreased | -2.155 | CD69,CD84,CLEC4A,CTLA4,IL2RG,MMP19,PTPN22,RHOH,SELPLG | 9 |
| Cellular Movement,Hematological System Development and Function,Immune Cell Trafficking,Inflammatory Response | Chemotaxis of mononuclear leukocytes | 6.27E-05 | Decreased | -2.153 | CXCL13,HP,PTPRO,RAC2,SPP1,XCL1 | 6 |
| Cell-To-Cell Signaling and Interaction | Adhesion of tumor cell lines | 4.36E-08 | Decreased | -2.131 | ADAM12,CXCL13,FERMT3,ITGAX,MMP19,PARVG,SELP,SELPLG,SH2B3,SPP1,VCAN | 11 |
| Inflammatory Response | Immune response of cells | 2.87E-07 | Decreased | -2.104 | BIRC3,CD69,CD80,CTLA4,FERMT3,IL2RG,IRF8,ITGAX,MSR1,RAC2,SELPLG,SIRPB1,SLAMF7,TYROBP | 14 |
| Cell-To-Cell Signaling and Interaction,Cellular Movement | Recruitment of cells | 1.33E-05 | Decreased | -2.102 | CD37,CD69,CXCL13,FERMT3,MMP12,PTHLH,SELP,SELPLG,SPP1 | 9 |
| Cellular Development,Cellular Growth and Proliferation,Hematological System Development and Function,Hematopoiesis,Lymphoid Tissue Structure and Development,Tissue Development | Leukopoiesis | 9.09E-09 | Decreased | -2.011 | ADAM12,CD69,CD80,CTLA4,EVI2B,IL2RG,IRF8,KLHL6,MMP19,MSR1,PTHLH,PTPN22,RAC2,RHOH,SELP,SH2B3,SPP1,TYROBP | 18 |
| Cell-To-Cell Signaling and Interaction,Inflammatory Response | Immune response of phagocytes | 0.000171 | Decreased | -2 | IRF8,ITGAX,SELPLG,SIRPB1,SLAMF7,TYROBP | 6 |
| Cell-To-Cell Signaling and Interaction,Cellular Function and Maintenance,Hematological System Development and Function | Phagocytosis of myeloid cells | 0.000347 | Decreased | -2 | IRF8,SELPLG,SIRPB1,SLAMF7,TYROBP | 5 |
| Cardiovascular System Development and Function,Cell-To-Cell Signaling and Interaction | Adhesion of vascular endothelial cells | 0.000379 | Decreased | -2 | FERMT3,SELP,SELPLG,SPP1 | 4 |
| Cell-To-Cell Signaling and Interaction,Cellular Function and Maintenance,Inflammatory Response | Phagocytosis of leukocytes | 0.00042 | Decreased | -2 | MSR1,SELPLG,SIRPB1,SLAMF7,TYROBP | 5 |
| Cellular Function and Maintenance | Engulfment of phagocytes | 0.000635 | Decreased | -2 | MSR1,SELPLG,SIRPB1,SLAMF7,TYROBP | 5 |
| Cell-mediated Immune Response,Cell-To-Cell Signaling and Interaction,Cellular Movement,Hematological System Development and Function,Immune Cell Trafficking | Adhesion of T lymphocytes | 2.24E-05 |  | -1.982 | CD80,RAC2,SELP,SELPLG,SPP1 | 5 |
| Cellular Movement | Transmigration of cells | 0.000535 |  | -1.982 | FERMT3,ITGAX,RAC2,SELPLG,SPP1 | 5 |
| Cellular Movement | Cell movement of cancer cells | 0.00123 |  | -1.982 | CXCL13,MMP13,SPP1,VCAN | 4 |
| Post-Translational Modification,Protein Degradation,Protein Synthesis | Proteolysis of Gelatin | 8.71E-07 |  | -1.98 | ADAM12,MMP12,MMP13,MMP19 | 4 |
| Cancer,Organismal Injury and Abnormalities | Carcinoma | 8.73E-07 |  | -1.98 | ADAM12,ADAMDEC1,ARHGAP9,BCAT1,BIRC3,CBLN2,CCDC71L,CCNA1,CD37,CD69,CD80,CD84,CLEC4A,CTLA4,CXCL13,CYTH4,DMXL2,EVI2B,FERMT3,FNDC3B,GABRA4,GASK1B,GLIPR1,GLIPR2,GLIS3,GPRIN3,HLA-DPA1,HP,IL18BP,IL2RG,INA,IRF8,ITGAX,KLHL6,LOXL2,LPXN,LY86,MMP12,MMP13,MMP19,MSR1,NCEH1,NRP2,P2RY10,P2RY8,PAPSS2,PARVG,PTHLH,PTPN22,PTPRO,PXMP4,RAC2,RHOH,SELP,SELPLG,SH2B3,SIRPB1,SLAMF7,SLC6A14,SNAP25,SPP1,TYROBP,VCAN,XCL1 | 64 |
| Cellular Development,Cellular Growth and Proliferation,Hematological System Development and Function,Hematopoiesis,Lymphoid Tissue Structure and Development,Tissue Development | Hematopoiesis of mononuclear leukocytes | 9.14E-06 |  | -1.98 | CD69,CD80,CTLA4,IL2RG,IRF8,KLHL6,MMP19,MSR1,PTPN22,RHOH,SH2B3,SPP1,TYROBP | 13 |
| Cell-mediated Immune Response,Cellular Movement,Hematological System Development and Function,Immune Cell Trafficking | NK cell migration | 1.74E-05 |  | -1.98 | FERMT3,SELP,SPP1,XCL1 | 4 |
| Cancer,Organismal Injury and Abnormalities | Advanced malignant solid tumor | 0.00039 |  | -1.98 | ADAM12,CCNA1,CTLA4,IL2RG,IRF8,KLHL6,P2RY8,PAPSS2,SELP,SPP1,VCAN | 11 |
| Cell-To-Cell Signaling and Interaction,Cellular Movement | Recruitment of lymphatic system cells | 0.000485 |  | -1.969 | CXCL13,PTHLH,SELP,SELPLG | 4 |
| Cell-To-Cell Signaling and Interaction,Hematological System Development and Function,Immune Cell Trafficking,Inflammatory Response | Adhesion of neutrophils | 6.61E-07 |  | -1.958 | CD37,FERMT3,ITGAX,PTPN22,SELP,SELPLG | 6 |
| Cell-mediated Immune Response,Cellular Movement,Hematological System Development and Function,Immune Cell Trafficking,Inflammatory Response,Lymphoid Tissue Structure and Development | Chemotaxis of T lymphocytes | 0.000333 |  | -1.95 | CXCL13,RAC2,SPP1,XCL1 | 4 |
| Cellular Development,Cellular Growth and Proliferation,Embryonic Development,Hematological System Development and Function,Hematopoiesis,Lymphoid Tissue Structure and Development,Organ Development,Organismal Development,Tissue Development | Lymphopoiesis | 2.36E-05 |  | -1.947 | CD69,CD80,CTLA4,IL2RG,IRF8,KLHL6,MMP19,MSR1,PTPN22,RHOH,SPP1,TYROBP | 12 |
| Connective Tissue Development and Function,Skeletal and Muscular System Development and Function | Resorption of bone | 0.000475 |  | -1.937 | ADAM12,PTHLH,RAC2,SPP1,TYROBP | 5 |
| Cellular Movement,Hematological System Development and Function,Immune Cell Trafficking | Cell movement of antigen presenting cells | 3.04E-06 |  | -1.924 | CD80,CXCL13,MMP12,NRP2,RAC2,SELP,SH2B3,SPP1,TYROBP,VCAN | 10 |
| Cellular Function and Maintenance | Homeostasis of mononuclear leukocytes | 4.82E-06 |  | -1.913 | CD69,CD80,CTLA4,IL2RG,IRF8,MMP19,MSR1,PTPN22,RHOH,SH2B3,SPP1,TYROBP | 12 |
| Cell-mediated Immune Response,Cellular Development,Cellular Function and Maintenance,Cellular Growth and Proliferation,Embryonic Development,Hematological System Development and Function,Hematopoiesis,Lymphoid Tissue Structure and Development,Organ Development,Organismal Development,Tissue Development | T cell development | 1.33E-05 |  | -1.909 | CD69,CD80,CTLA4,IL2RG,IRF8,MMP19,MSR1,PTPN22,RHOH,SPP1,TYROBP | 11 |
| Cancer,Organismal Injury and Abnormalities | Growth of tumor | 6.03E-05 |  | -1.877 | CCNA1,CD80,CTLA4,FERMT3,IRF8,LOXL2,PTHLH,PTPRO,RAC2,SELP,SELPLG,SH2B3,SPP1,VCAN | 14 |
| Cell-To-Cell Signaling and Interaction,Cellular Movement,Hematological System Development and Function,Immune Cell Trafficking | Recruitment of leukocytes | 4.26E-05 |  | -1.872 | CD37,CD69,CXCL13,FERMT3,MMP12,SELP,SELPLG,SPP1 | 8 |
| Cellular Development,Hematological System Development and Function,Lymphoid Tissue Structure and Development | Maturation of leukocytes | 0.00139 |  | -1.848 | CD69,CD80,IRF8,RHOH,TYROBP | 5 |
| Hematological System Development and Function,Lymphoid Tissue Structure and Development,Tissue Morphology | Quantity of T lymphocytes | 6.33E-09 |  | -1.847 | CD69,CD80,CD84,CLEC4A,CTLA4,IL2RG,IRF8,MMP19,PTPN22,RHOH,SELP,SELPLG,SH2B3,SPP1,XCL1 | 15 |
| Tissue Morphology | Quantity of cells | 7.97E-11 |  | -1.819 | ADAM12,BIRC3,CD69,CD80,CD84,CLEC4A,CTLA4,CXCL13,FERMT3,GLIS3,IL18BP,IL2RG,IRF8,KLHL6,MMP12,MMP13,MMP19,MSR1,NRP2,PTHLH,PTPN22,RAC2,RHOH,SELP,SELPLG,SH2B3,SPP1,TYROBP,VCAN,XCL1 | 30 |
| Cell-To-Cell Signaling and Interaction | Activation of cells | 1.2E-10 |  | -1.761 | ADAM12,CD37,CD69,CD80,CD84,CTLA4,HLA-DPA1,IL2RG,IRF8,ITGAX,LOXL2,MMP13,MMP19,MSR1,PTHLH,PTPN22,RHOH,SELP,SELPLG,SPP1,TYROBP,VCAN | 22 |
| Cellular Movement,Hematological System Development and Function,Immune Cell Trafficking | Cellular infiltration by granulocytes | 0.00025 |  | -1.758 | MMP12,MSR1,RAC2,SELP,SPP1,XCL1 | 6 |
| Cell-To-Cell Signaling and Interaction | Activation of connective tissue cells | 1.79E-05 |  | -1.757 | ADAM12,LOXL2,MMP13,PTHLH,SPP1,TYROBP | 6 |
| Cellular Movement,Hematological System Development and Function,Immune Cell Trafficking,Inflammatory Response | Migration of macrophages | 1.17E-06 |  | -1.755 | MMP12,RAC2,SH2B3,SPP1,TYROBP,VCAN | 6 |
| Immunological Disease,Inflammatory Disease,Inflammatory Response,Neurological Disease,Organismal Injury and Abnormalities | Experimental autoimmune encephalomyelitis | 7.84E-05 |  | -1.701 | CD80,CTLA4,IL18BP,IL2RG,ITGAX,PTPN22,SPP1,TYROBP | 8 |
| Cell-To-Cell Signaling and Interaction | Binding of tumor cell lines | 6.62E-10 |  | -1.693 | ADAM12,CD80,CLEC4A,CXCL13,FERMT3,ITGAX,MMP19,PARVG,RHOH,SELP,SELPLG,SH2B3,SPP1,VCAN | 14 |
| Cell-To-Cell Signaling and Interaction,Cellular Movement,Hematological System Development and Function,Immune Cell Trafficking,Inflammatory Response | Recruitment of neutrophils | 5.43E-06 |  | -1.673 | CD37,CD69,FERMT3,MMP12,SELP,SELPLG,SPP1 | 7 |
| Inflammatory Response,Neurological Disease | Inflammation of central nervous system | 5.46E-05 |  | -1.659 | CD80,CTLA4,GABRA4,IL18BP,IL2RG,ITGAX,PTPN22,SPP1,TYROBP | 9 |
| Cell-mediated Immune Response,Cellular Development,Cellular Function and Maintenance,Cellular Growth and Proliferation,Embryonic Development,Hematological System Development and Function,Hematopoiesis,Lymphoid Tissue Structure and Development,Organ Development,Organismal Development,Tissue Development | Differentiation of T lymphocytes | 0.000182 |  | -1.61 | CD69,CD80,CTLA4,IL2RG,IRF8,MMP19,PTPN22,RHOH | 8 |
| Cell Death and Survival,Cellular Compromise | Cytotoxicity of cells | 0.00013 |  | -1.536 | CD69,CTLA4,FERMT3,SLAMF7,TYROBP,XCL1 | 6 |
| Cellular Movement,Hematological System Development and Function,Immune Cell Trafficking | Cellular infiltration by leukocytes | 9.37E-06 |  | -1.524 | CD80,CTLA4,IL18BP,MMP12,MSR1,RAC2,SELP,SELPLG,SPP1,XCL1 | 10 |
| Connective Tissue Development and Function,Tissue Morphology | Quantity of connective tissue cells | 5.54E-05 |  | -1.486 | ADAM12,CXCL13,IRF8,MSR1,PTHLH,SPP1,TYROBP | 7 |
| Cell-To-Cell Signaling and Interaction | Binding of lymphoma cell lines | 4.45E-07 |  | -1.461 | CLEC4A,CXCL13,ITGAX,PARVG,SELPLG,VCAN | 6 |
| Cellular Movement,Hematological System Development and Function,Immune Cell Trafficking,Inflammatory Response | Cell movement of macrophages | 0.00017 |  | -1.454 | MMP12,RAC2,SELP,SH2B3,SPP1,TYROBP,VCAN | 7 |
| Hematological System Development and Function,Lymphoid Tissue Structure and Development,Tissue Morphology | Quantity of lymphocytes | 1.34E-10 |  | -1.446 | CD69,CD80,CD84,CLEC4A,CTLA4,CXCL13,IL2RG,IRF8,KLHL6,MMP19,PTPN22,RAC2,RHOH,SELP,SELPLG,SH2B3,SPP1,TYROBP,XCL1 | 19 |
| Hematological System Development and Function,Hematopoiesis,Lymphoid Tissue Structure and Development,Organ Morphology,Tissue Morphology | Quantity of double-positive thymocyte | 3.31E-05 |  | -1.446 | CD80,CTLA4,IL2RG,MMP19,RHOH | 5 |
| Cell-To-Cell Signaling and Interaction,Reproductive System Development and Function | Binding of gonadal cell lines | 2.95E-06 |  | -1.432 | CD80,CXCL13,IL2RG,ITGAX,SELP | 5 |
| Cell-To-Cell Signaling and Interaction,Connective Tissue Development and Function | Binding of fibroblast cell lines | 1.49E-06 |  | -1.387 | ADAM12,CD80,IL2RG,MSR1,SELPLG,SPP1 | 6 |
| Cell-To-Cell Signaling and Interaction | Response of myeloid cells | 2.92E-05 |  | -1.387 | CTLA4,IRF8,ITGAX,SELPLG,SIRPB1,SLAMF7,TYROBP | 7 |
| Cancer,Organismal Injury and Abnormalities | Cancer of cells | 1.35E-07 |  | -1.352 | ADAM12,BCAT1,BIRC3,CD69,CD80,CD84,CLEC4A,CTLA4,CXCL13,CYTH4,DMXL2,FNDC3B,GASK1B,GLIS3,IL2RG,INA,IRF8,ITGAX,KLHL6,LOXL2,MMP12,MMP13,MSR1,NRP2,P2RY10,P2RY8,PAPSS2,PTHLH,PTPN22,PTPRO,RAC2,RHOH,SELP,SELPLG,SH2B3,SIRPB1,SLAMF7,SNAP25,SPP1,VCAN | 40 |
| Cell Death and Survival | Cell viability of lymphatic system cells | 0.00117 |  | -1.346 | CD80,CTLA4,IL2RG,RAC2,SELPLG | 5 |
| Hematological System Development and Function,Lymphoid Tissue Structure and Development,Tissue Morphology | Quantity of helper T lymphocytes | 5.82E-05 |  | -1.289 | CD69,CD80,CTLA4,SELP,SELPLG | 5 |
| Cellular Movement | Cellular infiltration | 4.34E-06 |  | -1.273 | CD80,CTLA4,IL18BP,IRF8,MMP12,MSR1,RAC2,SELP,SELPLG,SPP1,XCL1 | 11 |
| Cell Death and Survival | Apoptosis of T lymphocytes | 0.0001 |  | -1.272 | CTLA4,IL2RG,PTPN22,RAC2,RHOH,SPP1,XCL1 | 7 |
| Cell Death and Survival | Cell viability of leukocytes | 6.18E-05 |  | -1.27 | CD80,CTLA4,IL2RG,MSR1,RAC2,SELPLG,TYROBP | 7 |
| Hematological System Development and Function,Lymphoid Tissue Structure and Development,Tissue Morphology | Quantity of regulatory T lymphocytes | 1.83E-08 |  | -1.227 | CD69,CD80,CTLA4,IRF8,PTPN22,SELP,SELPLG,XCL1 | 8 |
| Cell-To-Cell Signaling and Interaction,Hematological System Development and Function | Activation of blood cells | 1.32E-08 |  | -1.22 | CD37,CD69,CD80,CD84,CTLA4,HLA-DPA1,IL2RG,IRF8,MMP19,MSR1,PTPN22,RHOH,SELP,SELPLG,SPP1,TYROBP,VCAN | 17 |
| Cellular Movement,Hematological System Development and Function,Immune Cell Trafficking | Cellular infiltration by mononuclear leukocytes | 8.3E-07 |  | -1.22 | CD80,CTLA4,IL18BP,MMP12,SELP,SELPLG,SPP1,XCL1 | 8 |
| Hematological System Development and Function,Lymphoid Tissue Structure and Development,Tissue Morphology | Quantity of natural killer cells | 0.000798 |  | -1.217 | CD80,IL2RG,RHOH,TYROBP | 4 |
| Cell Death and Survival | Apoptosis of mononuclear leukocytes | 1.23E-05 |  | -1.176 | CD80,CTLA4,IL2RG,IRF8,PTPN22,RAC2,RHOH,SPP1,XCL1 | 9 |
| Cell-To-Cell Signaling and Interaction | Activation of lymphoid cells | 2.66E-07 |  | -1.172 | CD69,CD80,CTLA4,HLA-DPA1,IL2RG,IRF8,ITGAX,MMP19,PTPN22,SELP,SPP1,TYROBP | 12 |
| Cell-To-Cell Signaling and Interaction | Activation of bone cells | 0.00013 |  | -1.154 | ADAM12,PTHLH,SPP1,TYROBP | 4 |
| Cell Death and Survival,Cellular Compromise | Cytotoxicity of lymphocytes | 0.000194 |  | -1.154 | CD69,CTLA4,FERMT3,TYROBP,XCL1 | 5 |
| Hematological System Development and Function,Tissue Morphology | Quantity of leukocytes | 4.81E-15 |  | -1.149 | ADAM12,BIRC3,CD69,CD80,CD84,CLEC4A,CTLA4,CXCL13,FERMT3,IL18BP,IL2RG,IRF8,KLHL6,MMP12,MMP13,MMP19,PTPN22,RAC2,RHOH,SELP,SELPLG,SH2B3,SPP1,TYROBP,VCAN,XCL1 | 26 |
| Cancer,Organismal Injury and Abnormalities | Extracranial solid tumor | 0.00001 |  | -1.117 | ADAM12,ADAMDEC1,ARHGAP9,BCAT1,BIRC3,CBLN2,CCDC71L,CCNA1,CD37,CD69,CD80,CD84,CLEC4A,CTLA4,CXCL13,CYTH4,DMXL2,EVI2B,FERMT3,FNDC3B,GABRA4,GASK1B,GLIPR1,GLIPR2,GLIS3,GPRIN3,HLA-DPA1,HP,IL18BP,IL2RG,INA,IRF8,ITGAX,KLHL6,LOXL2,LPXN,LY86,MMP12,MMP13,MMP19,MSR1,NCEH1,NRP2,P2RY10,P2RY8,PAPSS2,PARVG,PTHLH,PTPN22,PTPRO,PXMP4,RAC2,RHOH,SELP,SELPLG,SH2B3,SIRPB1,SLAMF7,SLC6A14,SNAP25,SPP1,TYROBP,VCAN,XCL1 | 64 |
| Hematological System Development and Function | Hemostasis | 4.19E-05 |  | -1.111 | CD84,FERMT3,HP,PAPSS2,SELP,SELPLG,SH2B3,VCAN | 8 |
| Hematological System Development and Function,Tissue Morphology | Quantity of antigen presenting cells | 1.46E-10 |  | -1.075 | BIRC3,CLEC4A,CTLA4,CXCL13,IL2RG,IRF8,MMP12,SELP,SELPLG,SH2B3,SPP1,TYROBP,XCL1 | 13 |
| Cancer,Organismal Injury and Abnormalities,Reproductive System Disease | Growth of mammary tumor | 6.01E-05 |  | -1.067 | CCNA1,CD80,CTLA4,PTPRO,SPP1 | 5 |
| Hematological System Development and Function,Immune Cell Trafficking,Inflammatory Response,Tissue Development | Accumulation of phagocytes | 0.000199 |  | -1.067 | ITGAX,SELP,SELPLG,SPP1,TYROBP | 5 |
| Cellular Movement,Connective Tissue Development and Function | Cell movement of fibroblast cell lines | 0.000893 |  | -1.067 | LPXN,MSR1,RAC2,SPP1,XCL1 | 5 |
| Cardiovascular Disease,Organismal Injury and Abnormalities | Atherosclerosis | 2.23E-06 |  | -1.04 | BIRC3,GABRA4,HP,ITGAX,MMP12,MMP13,MSR1,NCEH1,SELP,SPP1,VCAN | 11 |
| Cell-To-Cell Signaling and Interaction | Binding of leukemia cell lines | 8.25E-05 |  | -0.992 | FERMT3,RHOH,SELP,SELPLG,SPP1 | 5 |
| Cell Death and Survival,Hematological System Development and Function | Cell viability of T lymphocytes | 0.000628 |  | -0.976 | CD80,CTLA4,IL2RG,SELPLG | 4 |
| Cellular Movement,Hematological System Development and Function,Immune Cell Trafficking | Cellular infiltration by lymphocytes | 3.44E-06 |  | -0.964 | CD80,CTLA4,IL18BP,SELP,SELPLG,SPP1,XCL1 | 7 |
| Cell-To-Cell Signaling and Interaction,Hematological System Development and Function,Immune Cell Trafficking,Inflammatory Response | Activation of lymphocytes | 2.04E-06 |  | -0.951 | CD69,CD80,CTLA4,HLA-DPA1,IL2RG,IRF8,MMP19,PTPN22,SELP,SPP1,TYROBP | 11 |
| Cellular Development,Cellular Growth and Proliferation,Connective Tissue Development and Function,Skeletal and Muscular System Development and Function,Tissue Development | Formation of osteoclasts | 0.00115 |  | -0.942 | ADAM12,IRF8,PTHLH,SPP1 | 4 |
| Cell Death and Survival | Apoptosis of lymphocytes | 0.000066 |  | -0.941 | CD80,CTLA4,IL2RG,PTPN22,RAC2,RHOH,SPP1,XCL1 | 8 |
| Hematological System Development and Function,Inflammatory Response,Tissue Morphology | Quantity of macrophages | 1.86E-06 |  | -0.933 | BIRC3,IL2RG,IRF8,MMP12,SELP,SELPLG,SH2B3,SPP1 | 8 |
| Cancer,Organismal Injury and Abnormalities,Reproductive System Disease | Mammary tumor | 1.78E-05 |  | -0.915 | ADAM12,BCAT1,CCDC71L,CCNA1,CD69,CD80,CTLA4,CYTH4,DMXL2,FERMT3,GASK1B,HP,IL2RG,ITGAX,KLHL6,LOXL2,MMP12,MMP13,MMP19,MSR1,NRP2,PTHLH,PTPRO,SPP1,VCAN | 25 |
| Hematological System Development and Function,Hypersensitivity Response,Inflammatory Response,Tissue Morphology | Quantity of mast cells | 2.01E-05 |  | -0.9 | ADAM12,IL2RG,RAC2,SELP | 4 |
| Tissue Development | Accumulation of cells | 8.31E-07 |  | -0.889 | CD80,CLEC4A,CTLA4,IL2RG,ITGAX,RAC2,SELP,SELPLG,SPP1,TYROBP | 10 |
| Hematological System Development and Function,Lymphoid Tissue Structure and Development,Tissue Morphology | Quantity of CD8+ T lymphocyte | 0.000332 |  | -0.849 | CLEC4A,IL2RG,MMP19,PTPN22,RHOH | 5 |
| Cell-To-Cell Signaling and Interaction | Aggregation of cells | 0.00163 |  | -0.849 | FERMT3,P2RY8,PTHLH,SELP,SELPLG,SH2B3 | 6 |
| Cell-To-Cell Signaling and Interaction,Hematological System Development and Function | Aggregation of blood cells | 0.00168 |  | -0.849 | FERMT3,P2RY8,SELP,SELPLG,SH2B3 | 5 |
| Cancer,Organismal Injury and Abnormalities | Neoplasia of cells | 1.12E-08 |  | -0.827 | ADAM12,ADAMDEC1,ARHGAP9,BCAT1,BIRC3,CCNA1,CD69,CD80,CD84,CLEC4A,CTLA4,CXCL13,CYTH4,DMXL2,FNDC3B,GABRA4,GASK1B,GLIPR2,GLIS3,IL2RG,INA,IRF8,ITGAX,KLHL6,LOXL2,MMP12,MMP13,MSR1,NRP2,P2RY10,P2RY8,PAPSS2,PTHLH,PTPN22,PTPRO,RAC2,RHOH,SELP,SELPLG,SH2B3,SIRPB1,SLAMF7,SNAP25,SPP1,VCAN | 45 |
| Cell Death and Survival | Cell death of immune cells | 7.42E-08 |  | -0.802 | BIRC3,CD69,CD80,CTLA4,IL2RG,IRF8,MSR1,NCEH1,PTPN22,RAC2,RHOH,SELPLG,SPP1,TYROBP,XCL1 | 15 |
| Hematological System Development and Function,Hematopoiesis,Lymphoid Tissue Structure and Development,Organ Morphology,Tissue Morphology | Quantity of thymocytes | 0.000191 |  | -0.797 | CD80,CLEC4A,CTLA4,IL2RG,MMP19,RHOH | 6 |
| Cell-To-Cell Signaling and Interaction,Hematological System Development and Function,Immune Cell Trafficking,Inflammatory Response | Activation of leukocytes | 2.04E-08 |  | -0.69 | CD37,CD69,CD80,CD84,CTLA4,HLA-DPA1,IL2RG,IRF8,MMP19,MSR1,PTPN22,RHOH,SELP,SPP1,TYROBP,VCAN | 16 |
| Hematological Disease,Immunological Disease | Eosinophilia | 5.58E-06 |  | -0.689 | ADAM12,ADAMDEC1,CD69,IL2RG,IRF8,MMP12,SPP1 | 7 |
| Cell-mediated Immune Response,Cellular Movement,Hematological System Development and Function,Immune Cell Trafficking | Infiltration by T lymphocytes | 5.04E-06 |  | -0.685 | CD80,CTLA4,IL18BP,SELP,SELPLG,XCL1 | 6 |
| Connective Tissue Development and Function,Skeletal and Muscular System Development and Function,Tissue Morphology | Quantity of osteoclasts | 0.00008 |  | -0.684 | IRF8,MSR1,PTHLH,SPP1,TYROBP | 5 |
| Cell-To-Cell Signaling and Interaction,Inflammatory Response | Immune response of leukocytes | 5.49E-07 |  | -0.626 | CD69,CD80,CTLA4,IRF8,ITGAX,MSR1,SELPLG,SIRPB1,SLAMF7,TYROBP | 10 |
| Cell-To-Cell Signaling and Interaction,Hematological System Development and Function,Immune Cell Trafficking,Inflammatory Response | Activation of T lymphocytes | 9.34E-06 |  | -0.61 | CD80,CTLA4,HLA-DPA1,IL2RG,IRF8,MMP19,PTPN22,SPP1,TYROBP | 9 |
| Cellular Movement | Cell movement of lymphoma cell lines | 8.21E-06 |  | -0.6 | CD69,CXCL13,PARVG,RAC2,SELPLG | 5 |
| Cell-To-Cell Signaling and Interaction | Response of lymphatic system cells | 0.00126 |  | -0.536 | CD80,CTLA4,IRF8,MSR1,SELPLG | 5 |
| Cellular Development,Cellular Growth and Proliferation,Lymphoid Tissue Structure and Development,Tissue Development | Proliferation of bone marrow cells | 0.000105 |  | -0.508 | IRF8,PTPRO,RAC2,SH2B3,SPP1 | 5 |
| Hematological System Development and Function,Immune Cell Trafficking,Inflammatory Response,Tissue Development | Accumulation of leukocytes | 8.18E-07 |  | -0.503 | CD80,CLEC4A,CTLA4,IL2RG,ITGAX,SELP,SELPLG,SPP1,TYROBP | 9 |
| Lymphoid Tissue Structure and Development,Organ Morphology,Tissue Morphology | Quantity of lymphoid organ | 1.46E-06 |  | -0.494 | CD80,CLEC4A,CTLA4,CXCL13,IL2RG,IRF8,MMP19,RHOH,SELP | 9 |
| Cancer,Organismal Injury and Abnormalities | Lymphoid cancer | 6.99E-07 |  | -0.447 | ADAM12,BCAT1,BIRC3,CD69,CD80,CLEC4A,CTLA4,CXCL13,FNDC3B,GLIS3,IL2RG,IRF8,ITGAX,KLHL6,MMP12,P2RY8,RAC2,RHOH,SELP,SELPLG,SH2B3,SLAMF7,SPP1,VCAN | 24 |
| Lymphoid Tissue Structure and Development,Tissue Morphology | Quantity of lymphoid tissue | 2.17E-10 |  | -0.44 | CD37,CD80,CD84,CLEC4A,CTLA4,CXCL13,IL2RG,IRF8,KLHL6,MMP19,PTPN22,RHOH,SELP,TYROBP | 14 |
| Cellular Development,Cellular Growth and Proliferation,Hematological System Development and Function,Hematopoiesis | Proliferation of hematopoietic cells | 0.000116 |  | -0.425 | IL2RG,IRF8,PTPN22,PTPRO,RAC2,SH2B3,SPP1 | 7 |
| Infectious Diseases | Viral Infection | 3.32E-05 |  | -0.416 | BIRC3,CD69,CD80,CLEC4A,CTLA4,FERMT3,GABRA4,HP,IL2RG,IRF8,MMP12,MSR1,P2RY10,RHOH,SELPLG,SH2B3,SPP1,TYROBP,XCL1 | 19 |
| Free Radical Scavenging | Production of reactive oxygen species | 1.92E-05 |  | -0.37 | CTLA4,ITGAX,MSR1,PTPN22,RAC2,SELP,SELPLG,SPP1,TYROBP | 9 |
| Cell Death and Survival | Apoptosis of leukocytes | 3.21E-08 |  | -0.345 | BIRC3,CD69,CD80,CTLA4,IL2RG,IRF8,MSR1,NCEH1,PTPN22,RAC2,RHOH,SPP1,XCL1 | 13 |
| Cellular Development,Cellular Growth and Proliferation,Hematological System Development and Function,Hematopoiesis,Lymphoid Tissue Structure and Development,Tissue Development | Differentiation of myeloid leukocytes | 0.000102 |  | -0.343 | EVI2B,IRF8,PTHLH,RAC2,SELP,SH2B3,TYROBP | 7 |
| Connective Tissue Development and Function,Tissue Morphology | Quantity of connective tissue | 0.000204 |  | -0.326 | ADAM12,CXCL13,IRF8,MSR1,PTHLH,RAC2,SELP,SH2B3,SPP1,TYROBP | 10 |
| Organismal Injury and Abnormalities | Fibrosis | 1.5E-06 |  | -0.32 | CXCL13,GLIS3,HP,IL2RG,MMP12,MMP13,MMP19,MSR1,PTHLH,SELP,SELPLG,SH2B3,SPP1 | 13 |
| Cancer,Organismal Injury and Abnormalities | Incidence of tumor | 0.000131 |  | -0.284 | ADAM12,ADAMDEC1,BCAT1,CCNA1,CD37,CD69,CD80,CTLA4,CYTH4,DMXL2,EVI2B,FNDC3B,GABRA4,GASK1B,GLIPR1,GLIPR2,GPRIN3,HP,IL2RG,INA,IRF8,ITGAX,KLHL6,LOXL2,LPXN,LY86,MMP12,MMP13,MMP19,MSR1,NCEH1,NRP2,P2RY10,P2RY8,PAPSS2,PARVG,PTHLH,PTPN22,PTPRO,RAC2,SELP,SELPLG,SH2B3,SIRPB1,SLAMF7,SLC6A14,SPP1,VCAN | 48 |
| Cellular Development,Cellular Growth and Proliferation,Hematological System Development and Function,Hematopoiesis | Proliferation of hematopoietic progenitor cells | 0.000615 |  | -0.263 | IL2RG,IRF8,PTPN22,PTPRO,SH2B3,SPP1 | 6 |
| Cell-To-Cell Signaling and Interaction,Hematological System Development and Function,Immune Cell Trafficking,Inflammatory Response | Activation of phagocytes | 0.000709 |  | -0.246 | CD37,CD80,CD84,MSR1,RHOH,SPP1,TYROBP | 7 |
| Cancer,Organismal Injury and Abnormalities | Lymphohematopoietic neoplasia | 5.79E-08 |  | -0.235 | ADAM12,ADAMDEC1,ARHGAP9,BCAT1,BIRC3,CCNA1,CD69,CD80,CLEC4A,CTLA4,CXCL13,FNDC3B,GABRA4,GLIPR2,GLIS3,IL2RG,IRF8,ITGAX,KLHL6,MMP12,MSR1,NRP2,P2RY8,RAC2,RHOH,SELP,SELPLG,SH2B3,SIRPB1,SLAMF7,SPP1,VCAN | 32 |
| Cancer,Organismal Injury and Abnormalities | Frequency of tumor | 0.000361 |  | -0.216 | ADAM12,ADAMDEC1,BCAT1,CCNA1,CD37,CD69,CTLA4,CYTH4,DMXL2,EVI2B,FNDC3B,GABRA4,GASK1B,GLIPR1,GLIPR2,GPRIN3,HP,IL2RG,IRF8,ITGAX,KLHL6,LOXL2,LPXN,LY86,MMP12,MMP13,MMP19,MSR1,NCEH1,NRP2,P2RY10,P2RY8,PAPSS2,PARVG,PTHLH,PTPN22,PTPRO,RAC2,SELP,SELPLG,SH2B3,SIRPB1,SLAMF7,SLC6A14,SPP1,VCAN | 46 |
| Connective Tissue Disorders,Organismal Injury and Abnormalities,Skeletal and Muscular Disorders,Tissue Morphology | Damage of bone | 2.59E-05 |  | -0.152 | CD80,CTLA4,GABRA4,IRF8,MMP13,SPP1 | 6 |
| Hematological System Development and Function,Hypersensitivity Response,Tissue Morphology | Quantity of eosinophils | 5.44E-05 |  | -0.128 | IL2RG,IRF8,MMP12,SELP,SELPLG | 5 |
| Embryonic Development,Organismal Development | Development of body trunk | 0.000272 |  | -0.092 | CD80,GLIS3,HP,IL2RG,LOXL2,MMP12,MMP13,NRP2,PTHLH,PTPRO,SELP,SH2B3,SPP1,TYROBP,VCAN | 15 |
| Free Radical Scavenging | Production of superoxide | 6.84E-05 |  | -0.072 | ITGAX,RAC2,SELP,SPP1,TYROBP | 5 |
| Free Radical Scavenging | Synthesis of reactive oxygen species | 3.33E-05 |  | -0.071 | CTLA4,HP,ITGAX,MSR1,PTPN22,RAC2,SELP,SELPLG,SPP1,TYROBP | 10 |
| Hematological System Development and Function,Lymphoid Tissue Structure and Development,Organ Development,Tissue Development | Growth of lymphoid organ | 0.000332 |  | -0.063 | CD80,CTLA4,IL2RG,SH2B3,SPP1 | 5 |
| Hematological System Development and Function,Inflammatory Response,Tissue Morphology | Quantity of dendritic cells | 1.33E-09 |  | -0.046 | CLEC4A,CTLA4,CXCL13,IL2RG,IRF8,SH2B3,SPP1,TYROBP,XCL1 | 9 |
| Hematological System Development and Function,Lymphoid Tissue Structure and Development,Tissue Morphology | Quantity of TREG cells | 8.21E-05 |  | -0.042 | CD69,CD80,CTLA4,PTPN22 | 4 |
| Hematological System Development and Function,Tissue Morphology | Quantity of myeloid cells | 6.32E-08 |  | -0.028 | ADAM12,BIRC3,CLEC4A,IL18BP,IL2RG,IRF8,MMP12,MMP13,RAC2,SELP,SELPLG,SH2B3,SPP1,TYROBP | 14 |
| Inflammatory Response | Cell-mediated response | 0.00101 |  | -0.027 | CD80,CTLA4,IL18BP,IL2RG,PTHLH | 5 |
| Cell Death and Survival | Necrosis | 2.29E-05 |  | -0.005 | ADAM12,BIRC3,CCNA1,CD69,CD80,CTLA4,GABRA4,GLIPR1,GLIS3,IL2RG,IRF8,MSR1,NCEH1,NRP2,PTHLH,PTPN22,PTPRO,RAC2,RHOH,SELP,SELPLG,SH2B3,SNAP25,SPP1,TYROBP,VCAN,XCL1 | 27 |
| Cancer,Hematological Disease,Immunological Disease,Organismal Injury and Abnormalities | Neoplasia of leukocytes | 6.12E-07 |  | 0 | ADAMDEC1,BCAT1,BIRC3,CD69,CD80,CLEC4A,CTLA4,CXCL13,FNDC3B,GLIS3,IL2RG,IRF8,ITGAX,KLHL6,MMP12,P2RY8,RAC2,RHOH,SELP,SELPLG,SH2B3,SLAMF7,SPP1,VCAN | 24 |
| Cancer,Hematological Disease,Immunological Disease,Organismal Injury and Abnormalities | Tumorigenesis of lymphocytes | 7.76E-07 |  | 0 | BCAT1,BIRC3,CD69,CD80,CLEC4A,CTLA4,CXCL13,FNDC3B,GLIS3,IL2RG,IRF8,ITGAX,KLHL6,MMP12,P2RY8,RAC2,RHOH,SELP,SELPLG,SH2B3,SLAMF7,SPP1,VCAN | 23 |
| Cancer,Hematological Disease,Immunological Disease,Organismal Injury and Abnormalities | Lymphocytic cancer | 2.28E-06 |  | 0 | BCAT1,BIRC3,CD69,CD80,CLEC4A,CTLA4,CXCL13,FNDC3B,GLIS3,IL2RG,IRF8,ITGAX,KLHL6,MMP12,P2RY8,RAC2,RHOH,SELP,SELPLG,SH2B3,SLAMF7,SPP1,VCAN | 23 |
| Cancer,Hematological Disease,Organismal Injury and Abnormalities | Lymphocytic neoplasm | 2.33E-06 |  | 0 | BCAT1,BIRC3,CD69,CD80,CLEC4A,CTLA4,CXCL13,FNDC3B,GLIS3,IL2RG,IRF8,ITGAX,KLHL6,MMP12,P2RY8,RAC2,RHOH,SELP,SELPLG,SH2B3,SLAMF7,SPP1,VCAN | 23 |
| Cancer,Hematological Disease,Immunological Disease,Organismal Injury and Abnormalities | Lymphoma | 3.19E-06 |  | 0 | BCAT1,BIRC3,CD69,CD80,CTLA4,CXCL13,FNDC3B,GLIS3,IL2RG,IRF8,ITGAX,KLHL6,MMP12,P2RY8,RAC2,SELP,SELPLG,SH2B3,SLAMF7 | 19 |
| Cancer,Hematological Disease,Organismal Injury and Abnormalities | Hematologic cancer of cells | 1.12E-05 |  | 0 | BCAT1,BIRC3,CD69,CD80,CTLA4,CXCL13,FNDC3B,GLIS3,IL2RG,IRF8,ITGAX,KLHL6,MMP12,P2RY8,RAC2,RHOH,SELP,SELPLG,SH2B3,SLAMF7,VCAN | 21 |
| Hematological System Development and Function,Hematopoiesis | Development of hematopoietic system | 0.00106 |  | 0 | IL2RG,IRF8,PTHLH,PTPN22,SELP,SH2B3,TYROBP | 7 |
| Cancer,Organismal Injury and Abnormalities | Lymphohematopoietic cancer | 1.57E-07 |  | 0.049 | ADAM12,ARHGAP9,BCAT1,BIRC3,CCNA1,CD69,CD80,CLEC4A,CTLA4,CXCL13,FNDC3B,GABRA4,GLIPR2,GLIS3,IL2RG,IRF8,ITGAX,KLHL6,MMP12,MSR1,NRP2,P2RY8,RAC2,RHOH,SELP,SELPLG,SH2B3,SIRPB1,SLAMF7,SPP1,VCAN | 31 |
| Organismal Survival | Survival of organism | 0.000481 |  | 0.05 | BIRC3,CD80,CTLA4,HP,IL2RG,IRF8,MMP12,PTHLH,RAC2,SNAP25,SPP1 | 11 |
| Protein Synthesis | Quantity of interleukin | 0.00132 |  | 0.068 | CLEC4A,SELP,SLC6A14,SPP1 | 4 |
| Connective Tissue Disorders,Inflammatory Disease,Organismal Injury and Abnormalities,Skeletal and Muscular Disorders | Rheumatic Disease | 3.43E-10 |  | 0.127 | ADAM12,CD69,CD80,CLEC4A,CTLA4,CXCL13,GABRA4,GLIPR2,HLA-DPA1,HP,IL18BP,IRF8,ITGAX,MMP12,MMP13,MMP19,MSR1,PTPN22,SELP,SLAMF7,SNAP25,SPP1,TYROBP,XCL1 | 24 |
| Cellular Development,Cellular Growth and Proliferation,Hematological System Development and Function,Lymphoid Tissue Structure and Development | Cell proliferation of T lymphocytes | 3.62E-10 |  | 0.129 | BIRC3,CD37,CD69,CD80,CD84,CTLA4,HLA-DPA1,IL2RG,IRF8,ITGAX,MMP19,PTPN22,RAC2,RHOH,SPP1,TYROBP,XCL1 | 17 |
| Cellular Compromise,Inflammatory Response | Degranulation of cells | 5.35E-07 |  | 0.152 | ARHGAP9,CD84,CTLA4,FERMT3,GLIPR1,HP,ITGAX,PTPN22,RAC2,SELP,SIRPB1,SNAP25,TYROBP | 13 |
| Cellular Compromise,Inflammatory Response | Degranulation of phagocytes | 1.98E-06 |  | 0.152 | ARHGAP9,CD84,CTLA4,GLIPR1,HP,ITGAX,PTPN22,RAC2,SIRPB1,SNAP25,TYROBP | 11 |
| Humoral Immune Response,Protein Synthesis | Quantity of IgG1 | 0.000951 |  | 0.152 | CD37,CD80,CLEC4A,PTPN22 | 4 |
| Hematological System Development and Function,Inflammatory Response,Tissue Morphology | Quantity of phagocytes | 1.97E-12 |  | 0.191 | ADAM12,BIRC3,CLEC4A,CTLA4,CXCL13,IL18BP,IL2RG,IRF8,MMP12,MMP13,RAC2,SELP,SELPLG,SH2B3,SPP1,TYROBP,XCL1 | 17 |
| Hematological System Development and Function,Humoral Immune Response,Lymphoid Tissue Structure and Development,Tissue Morphology | Quantity of B lymphocytes | 2.07E-10 |  | 0.198 | CD69,CD80,CD84,CLEC4A,CXCL13,IL2RG,IRF8,KLHL6,PTPN22,RAC2,SELP,SH2B3,SPP1,TYROBP | 14 |
| Connective Tissue Disorders,Inflammatory Disease,Inflammatory Response,Organismal Injury and Abnormalities,Skeletal and Muscular Disorders | Experimentally-induced arthritis | 1.03E-07 |  | 0.218 | CD69,CTLA4,CXCL13,MSR1,PTPN22,SELP,SPP1,XCL1 | 8 |
| Inflammatory Disease,Organismal Injury and Abnormalities,Respiratory Disease | Fibrosis of lung | 4.34E-05 |  | 0.225 | IL2RG,MMP12,MMP19,MSR1,SELP,SELPLG,SPP1 | 7 |
| Hematological System Development and Function,Immune Cell Trafficking,Inflammatory Response,Tissue Development | Accumulation of T lymphocytes | 0.000561 |  | 0.225 | CD80,CLEC4A,CTLA4,IL2RG | 4 |
| Inflammatory Response | Antibody response | 1.32E-06 |  | 0.246 | CD37,CD69,CD80,CTLA4,IRF8,KLHL6,TYROBP | 7 |
| Cancer,Hematological Disease,Organismal Injury and Abnormalities | Neoplasia of blood cells | 1.52E-07 |  | 0.266 | ADAMDEC1,ARHGAP9,BCAT1,BIRC3,CCNA1,CD69,CD80,CLEC4A,CTLA4,CXCL13,FNDC3B,GABRA4,GLIPR2,GLIS3,IL2RG,IRF8,ITGAX,KLHL6,MMP12,MSR1,NRP2,P2RY8,RAC2,RHOH,SELP,SELPLG,SH2B3,SIRPB1,SLAMF7,SPP1,VCAN | 31 |
| Cancer,Hematological Disease,Organismal Injury and Abnormalities | Myeloid or lymphoid neoplasm | 5.89E-07 |  | 0.266 | ARHGAP9,BCAT1,BIRC3,CCNA1,CD69,CD80,CLEC4A,CTLA4,CXCL13,FNDC3B,GABRA4,GLIPR2,GLIS3,IL2RG,IRF8,ITGAX,KLHL6,MMP12,MSR1,NRP2,P2RY8,RAC2,RHOH,SELP,SELPLG,SH2B3,SIRPB1,SLAMF7,SPP1,VCAN | 30 |
| Cancer,Organismal Injury and Abnormalities | Lymphoreticular neoplasm | 4.21E-06 |  | 0.266 | ARHGAP9,BCAT1,BIRC3,CCNA1,CD69,CD80,CTLA4,CXCL13,FNDC3B,GABRA4,GLIPR2,GLIS3,IL2RG,IRF8,ITGAX,KLHL6,MMP12,MSR1,NRP2,P2RY8,RAC2,SELP,SELPLG,SH2B3,SIRPB1,SLAMF7 | 26 |
| Connective Tissue Disorders,Inflammatory Disease,Inflammatory Response,Organismal Injury and Abnormalities,Skeletal and Muscular Disorders | Inflammation of joint | 6.75E-10 |  | 0.267 | ADAM12,CD69,CD80,CLEC4A,CTLA4,CXCL13,GABRA4,GLIPR2,HLA-DPA1,HP,IL18BP,MMP12,MMP13,MMP19,MSR1,PTPN22,SELP,SNAP25,SPP1,TYROBP,XCL1 | 21 |
| Humoral Immune Response,Lymphoid Tissue Structure and Development,Tissue Morphology | Quantity of germinal center | 7.44E-05 |  | 0.283 | CD37,CD84,PTPN22,TYROBP | 4 |
| Inflammatory Response | Inflammation of absolute anatomical region | 1.35E-05 |  | 0.313 | BIRC3,CD69,CD80,CLEC4A,CTLA4,GABRA4,HP,IL18BP,IL2RG,ITGAX,MMP12,MMP13,PTPN22,SELP,SPP1,TYROBP,XCL1 | 17 |
| Cancer,Hematological Disease,Organismal Injury and Abnormalities | Hematologic cancer | 5.21E-07 |  | 0.359 | ARHGAP9,BCAT1,BIRC3,CCNA1,CD69,CD80,CLEC4A,CTLA4,CXCL13,FNDC3B,GABRA4,GLIPR2,GLIS3,IL2RG,IRF8,ITGAX,KLHL6,MMP12,MSR1,NRP2,P2RY8,RAC2,RHOH,SELP,SELPLG,SH2B3,SIRPB1,SLAMF7,SPP1,VCAN | 30 |
| Lymphoid Tissue Structure and Development,Tissue Morphology | Quantity of lymph follicle | 8.84E-08 |  | 0.42 | CD37,CD80,CD84,IL2RG,IRF8,KLHL6,PTPN22,TYROBP | 8 |
| Cancer,Organismal Injury and Abnormalities | Liquid tumor | 0.00033 |  | 0.422 | ARHGAP9,BCAT1,BIRC3,CCNA1,CD80,CXCL13,FNDC3B,GABRA4,GLIPR2,IL2RG,IRF8,ITGAX,MSR1,NRP2,RAC2,RHOH,SH2B3,SIRPB1,VCAN | 19 |
| Cancer,Hematological Disease,Immunological Disease,Organismal Injury and Abnormalities | Leukemia | 0.000405 |  | 0.422 | ARHGAP9,BCAT1,BIRC3,CCNA1,CD80,CXCL13,GABRA4,GLIPR2,IL2RG,IRF8,ITGAX,MSR1,NRP2,RAC2,RHOH,SH2B3,SIRPB1,VCAN | 18 |
| Cell Death and Survival | Apoptosis of myeloid cells | 0.000989 |  | 0.43 | BIRC3,CD69,IRF8,MSR1,NCEH1 | 5 |
| Cellular Growth and Proliferation,Lymphoid Tissue Structure and Development | Proliferation of lymphatic system cells | 1.47E-12 |  | 0.44 | BIRC3,CD37,CD69,CD80,CD84,CLEC4A,CTLA4,HLA-DPA1,IL2RG,IRF8,ITGAX,LY86,MMP19,PTPN22,PTPRO,RAC2,RHOH,SH2B3,SLAMF7,SPP1,TYROBP,XCL1 | 22 |
| Cell-To-Cell Signaling and Interaction | Response of mononuclear leukocytes | 0.00186 |  | 0.478 | CD69,CD80,CTLA4,MSR1,SELPLG | 5 |
| Humoral Immune Response,Protein Synthesis | Quantity of IgG | 6.81E-09 |  | 0.539 | CD37,CD69,CD80,CD84,CLEC4A,IL2RG,LY86,PTPN22,SELP,TYROBP | 10 |
| Humoral Immune Response,Protein Synthesis | Quantity of IgG3 | 5.98E-06 |  | 0.555 | CD80,CLEC4A,IL2RG,LY86,TYROBP | 5 |
| Cellular Development,Cellular Growth and Proliferation,Hematological System Development and Function,Lymphoid Tissue Structure and Development | Proliferation of lymphocytes | 2.23E-12 |  | 0.576 | BIRC3,CD37,CD69,CD80,CD84,CLEC4A,CTLA4,HLA-DPA1,IL2RG,IRF8,ITGAX,LY86,MMP19,PTPN22,RAC2,RHOH,SH2B3,SLAMF7,SPP1,TYROBP,XCL1 | 21 |
| Hematological System Development and Function,Tissue Morphology | Quantity of granulocytes | 6.55E-08 |  | 0.59 | CLEC4A,IL18BP,IL2RG,IRF8,MMP12,MMP13,RAC2,SELP,SELPLG,SH2B3,SPP1 | 11 |
| Cellular Development,Cellular Growth and Proliferation | Proliferation of blood cells | 8.32E-12 |  | 0.621 | BIRC3,CD37,CD69,CD80,CD84,CLEC4A,CTLA4,HLA-DPA1,IL2RG,IRF8,ITGAX,LY86,MMP19,PTPN22,PTPRO,RAC2,RHOH,SH2B3,SLAMF7,SPP1,TYROBP,XCL1 | 22 |
| Humoral Immune Response,Protein Synthesis | Quantity of IgM | 6.07E-06 |  | 0.64 | CD69,CD80,IL2RG,IRF8,LY86,MSR1 | 6 |
| Hematological System Development and Function,Humoral Immune Response,Lymphoid Tissue Structure and Development,Tissue Morphology | Quantity of follicular B lymphocytes | 7.26E-06 |  | 0.651 | CD80,CD84,IRF8,KLHL6,PTPN22,TYROBP | 6 |
| Hematological System Development and Function,Hematopoiesis,Humoral Immune Response,Lymphoid Tissue Structure and Development,Tissue Morphology | Quantity of pre-B lymphocytes | 0.00102 |  | 0.689 | CD69,IL2RG,IRF8,SH2B3 | 4 |
| Embryonic Development,Hematological System Development and Function,Lymphoid Tissue Structure and Development,Organ Development,Organismal Development,Tissue Development | Formation of lymphoid tissue | 1.27E-08 |  | 0.728 | CD37,CD80,CD84,CTLA4,CXCL13,IL2RG,KLHL6,NRP2,PTPN22,SELP,SH2B3,TYROBP | 12 |
| Hematological Disease,Immunological Disease,Organismal Injury and Abnormalities | Leukocytosis | 0.00102 |  | 0.762 | BIRC3,RAC2,SELP,SPP1 | 4 |
| Inflammatory Response,Organismal Injury and Abnormalities | Inflammation of organ | 3.29E-07 |  | 0.846 | ADAMDEC1,BIRC3,CD69,CD80,CLEC4A,CTLA4,CXCL13,GABRA4,HP,IL18BP,IL2RG,ITGAX,MMP12,MMP13,MMP19,PTPN22,SELP,SELPLG,SPP1,TYROBP,XCL1 | 21 |
| Hematological System Development and Function,Inflammatory Response,Tissue Morphology | Quantity of neutrophils | 2.8E-08 |  | 0.871 | CLEC4A,IL18BP,IL2RG,MMP12,MMP13,RAC2,SELP,SELPLG,SH2B3,SPP1 | 10 |
| Connective Tissue Disorders,Inflammatory Disease,Inflammatory Response,Organismal Injury and Abnormalities,Skeletal and Muscular Disorders | Collagen-induced arthritis | 0.000404 |  | 0.958 | CD69,CTLA4,SELP,SPP1 | 4 |
| Connective Tissue Disorders,Inflammatory Disease,Inflammatory Response,Organismal Injury and Abnormalities,Skeletal and Muscular Disorders | Polyarthritis | 0.000447 |  | 0.958 | CD69,CD80,CTLA4,SELP,SPP1 | 5 |
| Cellular Development,Cellular Growth and Proliferation,Hematological System Development and Function,Lymphoid Tissue Structure and Development | Proliferation of naive lymphocytes | 9.94E-06 |  | 1 | CD69,CD80,CTLA4,SLAMF7 | 4 |
| Digestive System Development and Function,Gastrointestinal Disease,Hepatic System Development and Function,Hepatic System Disease,Inflammatory Disease,Inflammatory Response,Organ Development,Organismal Injury and Abnormalities | Inflammation of liver | 0.000066 |  | 1 | BIRC3,CTLA4,GABRA4,HP,IL2RG,SPP1,TYROBP,XCL1 | 8 |
| Gastrointestinal Disease,Inflammatory Disease | Gastroenteritis | 0.000104 |  | 1 | CD69,CD80,CTLA4,GABRA4,IL2RG,PTPN22,SELP,SPP1,XCL1 | 9 |
| Gastrointestinal Disease,Inflammatory Disease,Inflammatory Response,Organismal Injury and Abnormalities | Inflammation of the large intestine | 0.000231 |  | 1 | CD69,CD80,CTLA4,GABRA4,IL2RG,PTPN22,SELP,SPP1 | 8 |
| Humoral Immune Response,Protein Synthesis | Quantity of immunoglobulin | 8.53E-10 |  | 1.031 | CD37,CD69,CD80,CD84,CLEC4A,IL2RG,IRF8,LY86,MSR1,PTPN22,SELP,TYROBP | 12 |
| Cellular Development,Cellular Growth and Proliferation,Hematological System Development and Function,Lymphoid Tissue Structure and Development | Expansion of T lymphocytes | 0.000277 |  | 1.043 | CD69,CD80,CTLA4,IRF8,PTPN22 | 5 |
| Embryonic Development,Hematological System Development and Function,Lymphoid Tissue Structure and Development,Organ Development,Organismal Development,Tissue Development | Formation of lymphoid organ | 1.07E-07 |  | 1.067 | CD37,CD80,CD84,CXCL13,IL2RG,KLHL6,PTPN22,SELP,SH2B3,TYROBP | 10 |
| Hematological System Development and Function,Hematopoiesis,Tissue Morphology | Quantity of hematopoietic progenitor cells | 2.84E-07 |  | 1.185 | CD69,CD80,CLEC4A,CTLA4,IL2RG,IRF8,MMP19,RAC2,RHOH,SELP,SH2B3,SPP1 | 12 |
| Humoral Immune Response,Protein Synthesis | Production of antibody | 1.26E-10 |  | 1.206 | CD37,CD69,CD80,CD84,CLEC4A,CTLA4,IL2RG,IRF8,LY86,MSR1,PTPN22,SELP,TYROBP | 13 |
| Hematological Disease | Polycythemia | 0.00117 |  | 1.253 | BIRC3,RAC2,SELP,SH2B3,SPP1 | 5 |
| Cellular Development,Cellular Growth and Proliferation,Hematological System Development and Function,Humoral Immune Response,Lymphoid Tissue Structure and Development | Proliferation of B lymphocytes | 2.12E-06 |  | 1.281 | CD80,CLEC4A,CTLA4,IL2RG,IRF8,LY86,SH2B3,SLAMF7,TYROBP | 9 |
| Cellular Development,Cellular Growth and Proliferation,Lymphoid Tissue Structure and Development | Expansion of lymphoid cells | 6.72E-05 |  | 1.306 | CD69,CD80,CTLA4,IRF8,PTPN22,SH2B3 | 6 |
| Cellular Development,Cellular Growth and Proliferation | Expansion of blood cells | 3.66E-06 |  | 1.325 | CD69,CD80,CTLA4,IRF8,PTPN22,RAC2,SH2B3,SPP1 | 8 |
| Cellular Development,Cellular Growth and Proliferation,Hematological System Development and Function,Lymphoid Tissue Structure and Development | Expansion of leukocytes | 0.000106 |  | 1.359 | CD69,CD80,CTLA4,IRF8,PTPN22,SPP1 | 6 |
| Humoral Immune Response,Protein Synthesis | Quantity of IgG2a | 1.98E-05 |  | 1.387 | CD69,CD80,CLEC4A,PTPN22,TYROBP | 5 |
| Hematological System Development and Function,Inflammatory Response,Tissue Morphology | Quantity of monocytes | 0.00008 |  | 1.408 | CLEC4A,IL2RG,IRF8,SELP,SH2B3 | 5 |
| Cell Death and Survival | Apoptosis of hematopoietic cell lines | 0.00079 |  | 1.432 | BIRC3,CD69,CTLA4,IRF8,SPP1 | 5 |
| Inflammatory Response | Inflammation of body cavity | 2.25E-05 |  | 1.439 | BIRC3,CD69,CD80,CTLA4,GABRA4,HP,IL2RG,ITGAX,MMP12,MMP13,PTPN22,SELP,SPP1,TYROBP,XCL1 | 15 |
| Gastrointestinal Disease,Inflammatory Response | Inflammation of gastrointestinal tract | 8.74E-07 |  | 1.465 | ADAMDEC1,CD69,CD80,CLEC4A,CTLA4,GABRA4,IL2RG,MMP12,PTPN22,SELP,SPP1,XCL1 | 12 |
| Infectious Diseases | Infection of mammalia | 3.25E-05 |  | 1.601 | IL2RG,IRF8,ITGAX,MMP12,MSR1,RAC2,SELP,SPP1 | 8 |
| Inflammatory Response | Inflammation of secretory structure | 0.0006 |  | 1.702 | CD80,CLEC4A,CTLA4,GABRA4,XCL1 | 5 |
| Endocrine System Disorders,Gastrointestinal Disease,Metabolic Disease,Organismal Injury and Abnormalities | Diabetes mellitus | 4.64E-13 |  | 1.813 | CD80,CD84,CLEC4A,CTLA4,CXCL13,GABRA4,GLIPR2,GLIS3,HLA-DPA1,HP,IL18BP,ITGAX,LY86,MSR1,NRP2,P2RY10,PTHLH,PTPN22,PTPRO,RAC2,SELP,SH2B3,SIRPB1,SNAP25,SPP1,TYROBP,XCL1 | 27 |
| Developmental Disorder,Immunological Disease,Organismal Injury and Abnormalities | Hypoplasia of lymphoid organ | 0.00177 |  | 1.937 | CTLA4,IL2RG,KLHL6,RHOH | 4 |
| Cancer,Immunological Disease,Organismal Injury and Abnormalities | Hyperplasia of spleen | 6.05E-05 | Increased | 2 | IL2RG,IRF8,SELP,SH2B3 | 4 |
| Lymphoid Tissue Structure and Development,Tissue Morphology | Quantity of bone marrow cells | 0.00037 | Increased | 2.169 | IL2RG,IRF8,SELP,SH2B3,SPP1 | 5 |
| Endocrine System Disorders,Gastrointestinal Disease,Immunological Disease,Metabolic Disease,Organismal Injury and Abnormalities | Insulin-dependent diabetes mellitus | 1.44E-11 |  |  | CD80,CD84,CLEC4A,CTLA4,CXCL13,HLA-DPA1,IL18BP,ITGAX,LY86,P2RY10,PTPN22,PTPRO,SH2B3,SIRPB1,TYROBP,XCL1 | 16 |
| Immunological Disease | Systemic autoimmune syndrome | 1.88E-11 |  |  | ADAM12,CD69,CD80,CD84,CLEC4A,CTLA4,CXCL13,GABRA4,GLIPR2,HLA-DPA1,HP,IL18BP,IRF8,ITGAX,LY86,MMP13,P2RY10,PTPN22,PTPRO,SELP,SH2B3,SIRPB1,SLAMF7,SPP1,TYROBP,XCL1 | 26 |
| Cellular Function and Maintenance | Function of leukocytes | 3.65E-11 |  |  | BCAT1,BIRC3,CD69,CD80,CD84,CTLA4,IL2RG,IRF8,MMP12,MSR1,NCEH1,PTPN22,RAC2,SELP,SPP1,TYROBP | 16 |
| Cancer,Dermatological Diseases and Conditions,Organismal Injury and Abnormalities | Skin tumor | 1.63E-09 |  |  | ADAM12,ADAMDEC1,ARHGAP9,BCAT1,BIRC3,CBLN2,CCNA1,CD80,CD84,CTLA4,CXCL13,CYTH4,DMXL2,GABRA4,GASK1B,GLIPR1,GLIS3,GPRIN3,HP,IL2RG,INA,IRF8,ITGAX,KLHL6,LOXL2,LPXN,LY86,MMP13,MMP19,MSR1,NCEH1,NRP2,P2RY10,P2RY8,PAPSS2,PARVG,PTHLH,PTPN22,PTPRO,RAC2,RHOH,SELP,SELPLG,SH2B3,SIRPB1,SLAMF7,SLC6A14,SNAP25,SPP1,TYROBP,VCAN,XCL1 | 52 |
| Embryonic Development,Hematological System Development and Function,Lymphoid Tissue Structure and Development,Organ Development,Organismal Development,Tissue Development | Formation of lymph node | 3.35E-09 |  |  | CD37,CD84,CXCL13,IL2RG,KLHL6,PTPN22,SELP,TYROBP | 8 |
| Hematological System Development and Function,Lymphoid Tissue Structure and Development,Tissue Morphology | Morphology of lymphoid tissue | 3.74E-09 |  |  | CD37,CD80,CD84,CLEC4A,CTLA4,HP,IL2RG,IRF8,KLHL6,MSR1,PTPN22,RHOH,SELP,SH2B3,TYROBP | 15 |
| Embryonic Development,Hematological System Development and Function,Humoral Immune Response,Lymphoid Tissue Structure and Development,Organ Development,Organismal Development,Tissue Development,Tissue Morphology | Morphology of germinal center | 7E-09 |  |  | CD37,CD84,IL2RG,KLHL6,PTPN22,SELP,TYROBP | 7 |
| Hematological System Development and Function,Tissue Morphology | Abnormal quantity of leukocytes | 1.18E-08 |  |  | BIRC3,CD80,IL2RG,IRF8,RHOH,SELP,SH2B3 | 7 |
| Connective Tissue Disorders,Organismal Injury and Abnormalities,Skeletal and Muscular Disorders | Non-traumatic arthropathy | 1.28E-08 |  |  | ADAM12,CD69,CD80,CLEC4A,CTLA4,CXCL13,GABRA4,GLIPR2,HLA-DPA1,HP,IL18BP,MMP12,MMP13,MMP19,PTPN22,SNAP25,SPP1,XCL1 | 18 |
| Cellular Function and Maintenance,Hematological System Development and Function | Function of lymphocytes | 1.44E-08 |  |  | BCAT1,BIRC3,CD69,CD80,CD84,CTLA4,IL2RG,IRF8,PTPN22,SELP,TYROBP | 11 |
| Cellular Function and Maintenance,Hematological System Development and Function | Function of T lymphocytes | 2.64E-08 |  |  | BCAT1,BIRC3,CD69,CD80,CD84,CTLA4,IRF8,PTPN22,SELP,TYROBP | 10 |
| Hematological System Development and Function,Immunological Disease,Lymphoid Tissue Structure and Development,Organ Morphology,Organismal Development,Organismal Injury and Abnormalities,Tissue Morphology | Abnormal morphology of spleen | 3.53E-08 |  |  | CD80,CTLA4,HP,IL2RG,IRF8,KLHL6,MSR1,PTPN22,RHOH,SELP,SH2B3 | 11 |
| Hematological System Development and Function,Immunological Disease,Lymphoid Tissue Structure and Development,Organ Morphology,Organismal Injury and Abnormalities,Tissue Morphology | Abnormal morphology of lymph node | 4.2E-08 |  |  | CD80,CLEC4A,CTLA4,IL2RG,IRF8,PTPN22,SELP,SH2B3 | 8 |
| Cancer,Organismal Injury and Abnormalities | Malignant solid organ tumor | 5.87E-08 |  |  | ADAM12,ADAMDEC1,ARHGAP9,BCAT1,BIRC3,CBLN2,CCNA1,CD84,CTLA4,CXCL13,CYTH4,DMXL2,GABRA4,GASK1B,GLIPR1,GLIS3,GPRIN3,HP,IL2RG,INA,IRF8,ITGAX,KLHL6,LOXL2,LPXN,LY86,MMP13,MSR1,NCEH1,NRP2,P2RY10,P2RY8,PAPSS2,PARVG,PTHLH,PTPN22,PTPRO,RAC2,RHOH,SELP,SELPLG,SH2B3,SIRPB1,SLAMF7,SLC6A14,SNAP25,SPP1,TYROBP,VCAN,XCL1 | 50 |
| Hematological System Development and Function,Immunological Disease,Lymphoid Tissue Structure and Development,Organ Morphology,Organismal Injury and Abnormalities,Tissue Morphology | Abnormal morphology of lymphoid organ | 6.19E-08 |  |  | CD80,CLEC4A,CTLA4,HP,IL2RG,IRF8,KLHL6,MSR1,PTPN22,RHOH,SELP,SH2B3 | 12 |
| Cancer,Dermatological Diseases and Conditions,Organismal Injury and Abnormalities | Skin cancer | 1.26E-07 |  |  | ADAM12,ADAMDEC1,ARHGAP9,BCAT1,BIRC3,CBLN2,CCNA1,CD84,CTLA4,CXCL13,CYTH4,DMXL2,GABRA4,GASK1B,GLIPR1,GLIS3,GPRIN3,HP,IL2RG,INA,IRF8,ITGAX,KLHL6,LOXL2,LPXN,LY86,MMP13,MSR1,NCEH1,NRP2,P2RY10,P2RY8,PAPSS2,PARVG,PTHLH,PTPN22,PTPRO,RAC2,RHOH,SELP,SELPLG,SH2B3,SIRPB1,SLAMF7,SLC6A14,SNAP25,TYROBP,VCAN,XCL1 | 49 |
| Connective Tissue Disorders,Inflammatory Disease,Inflammatory Response,Organismal Injury and Abnormalities,Skeletal and Muscular Disorders | Osteoarthritis | 2.15E-07 |  |  | ADAM12,CD80,CTLA4,GABRA4,MMP12,MMP13,MMP19,SNAP25,SPP1 | 9 |
| Immunological Disease | Abnormal morphology of immune system | 2.23E-07 |  |  | CD80,CD84,CLEC4A,CTLA4,IL2RG,IRF8,PTPN22,RHOH,SPP1,XCL1 | 10 |
| Inflammatory Disease | Chronic inflammatory disorder | 2.49E-07 |  |  | ADAM12,BIRC3,CD69,CD80,CLEC4A,CTLA4,CXCL13,GABRA4,GLIPR2,HLA-DPA1,HP,IL18BP,IL2RG,MMP13,PTPN22,SELP,SNAP25,SPP1,XCL1 | 19 |
| Connective Tissue Disorders,Immunological Disease,Inflammatory Disease,Inflammatory Response,Organismal Injury and Abnormalities,Skeletal and Muscular Disorders | Rheumatoid arthritis | 3.88E-07 |  |  | ADAM12,CD69,CD80,CLEC4A,CTLA4,CXCL13,GABRA4,GLIPR2,HLA-DPA1,HP,IL18BP,MMP13,PTPN22,SPP1,XCL1 | 15 |
| Cell Morphology,Lymphoid Tissue Structure and Development | Morphology of lymphatic system cells | 3.95E-07 |  |  | CD80,CD84,CTLA4,IL2RG,IRF8,PTPN22,RHOH,SELP,SH2B3 | 9 |
| Hematological System Development and Function,Immunological Disease,Lymphoid Tissue Structure and Development,Organ Morphology,Organismal Injury and Abnormalities,Tissue Morphology | Abnormal morphology of enlarged lymph node | 3.95E-07 |  |  | CLEC4A,CTLA4,IRF8,PTPN22,SELP,SH2B3 | 6 |
| Cellular Function and Maintenance,Hematological System Development and Function | Function of myeloid cells | 4.86E-07 |  |  | BIRC3,IL2RG,MMP12,MSR1,NCEH1,RAC2,SPP1,TYROBP | 8 |
| Cancer,Dermatological Diseases and Conditions,Organismal Injury and Abnormalities | Cutaneous melanoma | 1.05E-06 |  |  | ADAMDEC1,ARHGAP9,BCAT1,BIRC3,CBLN2,CCNA1,CD84,CTLA4,CYTH4,DMXL2,GABRA4,GASK1B,GLIPR1,GLIS3,GPRIN3,HP,IL2RG,INA,IRF8,ITGAX,KLHL6,LOXL2,LPXN,LY86,MMP13,MSR1,NCEH1,NRP2,P2RY10,P2RY8,PAPSS2,PARVG,PTHLH,PTPN22,PTPRO,RAC2,SELP,SELPLG,SH2B3,SIRPB1,SLAMF7,SLC6A14,SNAP25,TYROBP,VCAN,XCL1 | 46 |
| Embryonic Development,Hematological System Development and Function,Humoral Immune Response,Immunological Disease,Lymphoid Tissue Structure and Development,Organ Development,Organismal Development,Organismal Injury and Abnormalities,Tissue Development,Tissue Morphology | Abnormal morphology of germinal center | 1.19E-06 |  |  | CD37,IL2RG,KLHL6,PTPN22,SELP | 5 |
| Cell Morphology | Morphology of blood cells | 1.37E-06 |  |  | CD80,CD84,CTLA4,FERMT3,IL2RG,IRF8,PTPN22,RHOH,SELP,SH2B3,SPP1 | 11 |
| Cancer,Hematological Disease,Organismal Injury and Abnormalities | Mature lymphocytic neoplasm | 3.49E-06 |  |  | BCAT1,BIRC3,CD69,CD80,CLEC4A,CXCL13,FNDC3B,GLIS3,IL2RG,IRF8,ITGAX,KLHL6,MMP12,P2RY8,RHOH,SELP,SELPLG,SLAMF7,SPP1,VCAN | 20 |
| Cancer,Organismal Injury and Abnormalities | Adenocarcinoma | 4.25E-06 |  |  | ADAM12,ADAMDEC1,BCAT1,BIRC3,CBLN2,CCDC71L,CCNA1,CD37,CD69,CD80,CD84,CLEC4A,CTLA4,CYTH4,DMXL2,EVI2B,FERMT3,FNDC3B,GABRA4,GASK1B,GLIPR1,GLIPR2,GPRIN3,IL18BP,IL2RG,INA,IRF8,ITGAX,KLHL6,LOXL2,LPXN,LY86,MMP12,MMP13,MMP19,MSR1,NCEH1,NRP2,P2RY10,P2RY8,PAPSS2,PARVG,PTHLH,PTPN22,PTPRO,PXMP4,RAC2,RHOH,SELP,SELPLG,SH2B3,SIRPB1,SLAMF7,SLC6A14,SNAP25,SPP1,TYROBP,VCAN,XCL1 | 59 |
| Cell Morphology,Immunological Disease,Lymphoid Tissue Structure and Development | Abnormal morphology of lymphocytes | 4.46E-06 |  |  | CD80,CD84,CTLA4,IL2RG,IRF8,PTPN22,RHOH | 7 |
| Organismal Development | Abnormal morphology of body cavity | 4.53E-06 |  |  | ADAM12,BIRC3,CD69,CD80,CTLA4,GLIS3,HP,IL2RG,IRF8,KLHL6,LOXL2,MMP12,MSR1,PTHLH,PTPN22,PTPRO,RHOH,SELP,SH2B3,SPP1 | 20 |
| Cellular Function and Maintenance | Function of phagocytes | 5.35E-06 |  |  | BIRC3,IL2RG,MMP12,MSR1,NCEH1,RAC2,SPP1,TYROBP | 8 |
| Cancer,Organismal Injury and Abnormalities | Abdominal carcinoma | 6.89E-06 |  |  | ADAM12,ADAMDEC1,ARHGAP9,BCAT1,BIRC3,CBLN2,CCNA1,CD37,CD69,CD80,CD84,CLEC4A,CTLA4,CYTH4,DMXL2,EVI2B,FERMT3,FNDC3B,GABRA4,GASK1B,GLIPR1,GLIPR2,GPRIN3,HLA-DPA1,HP,IL18BP,IL2RG,INA,IRF8,ITGAX,KLHL6,LOXL2,LPXN,LY86,MMP12,MMP13,MMP19,MSR1,NCEH1,NRP2,P2RY10,P2RY8,PAPSS2,PARVG,PTHLH,PTPN22,PTPRO,PXMP4,RAC2,RHOH,SELP,SELPLG,SH2B3,SIRPB1,SLAMF7,SLC6A14,SNAP25,SPP1,VCAN,XCL1 | 60 |
| Cancer,Organismal Injury and Abnormalities | Melanoma | 7.53E-06 |  |  | ADAMDEC1,ARHGAP9,BCAT1,BIRC3,CBLN2,CCNA1,CD69,CD84,CTLA4,CYTH4,DMXL2,FNDC3B,GABRA4,GASK1B,GLIPR1,GLIS3,GPRIN3,HP,IL2RG,INA,IRF8,ITGAX,KLHL6,LOXL2,LPXN,LY86,MMP13,MSR1,NCEH1,NRP2,P2RY10,P2RY8,PAPSS2,PARVG,PTHLH,PTPN22,PTPRO,RAC2,SELP,SELPLG,SH2B3,SIRPB1,SLAMF7,SLC6A14,SNAP25,SPP1,TYROBP,VCAN,XCL1 | 49 |
| Cardiovascular Disease,Organismal Injury and Abnormalities | Advanced stage peripheral arterial disease | 7.78E-06 |  |  | CLEC4A,EVI2B,IRF8,LPXN,MSR1,SPP1 | 6 |
| Connective Tissue Disorders,Hereditary Disorder,Immunological Disease,Inflammatory Disease,Organismal Injury and Abnormalities,Skeletal and Muscular Disorders | Susceptibility to systemic lupus erythematosus | 7.97E-06 |  |  | CTLA4,IRF8,PTPN22 | 3 |
| Endocrine System Disorders,Immunological Disease,Inflammatory Disease,Organismal Injury and Abnormalities | Susceptibility to Hashimoto thyroiditis | 8.07E-06 |  |  | CTLA4,PTPN22 | 2 |
| Cellular Assembly and Organization | Binding of microparticles | 8.07E-06 |  |  | SELP,SELPLG | 2 |
| Cell Morphology,Hematopoiesis | Abnormal morphology of hematopoietic progenitor cells | 1.02E-05 |  |  | IL2RG,IRF8,PTPN22,RHOH,SELP,SH2B3 | 6 |
| Dermatological Diseases and Conditions,Infectious Diseases,Organismal Injury and Abnormalities | Lepromatous leprosy | 1.08E-05 |  |  | CD80,IL2RG,LY86,MSR1 | 4 |
| Connective Tissue Development and Function,Skeletal and Muscular System Development and Function,Tissue Development | Volume of bone | 1.24E-05 |  |  | CLEC4A,MMP13,MSR1,PTHLH,SPP1,TYROBP | 6 |
| Immunological Disease,Neurological Disease,Skeletal and Muscular Disorders | Myasthenia gravis | 1.39E-05 |  |  | CD80,CTLA4,CXCL13,IRF8,SNAP25 | 5 |
| Connective Tissue Disorders,Immunological Disease,Inflammatory Disease,Organismal Injury and Abnormalities,Skeletal and Muscular Disorders | Systemic lupus erythematosus | 1.48E-05 |  |  | CD69,CD80,CTLA4,IRF8,ITGAX,PTPN22,SELP,SLAMF7,SPP1 | 9 |
| Endocrine System Disorders,Immunological Disease,Organismal Injury and Abnormalities | Autoimmune thyroid disease | 1.49E-05 |  |  | CD80,CTLA4,PTPN22,SNAP25 | 4 |
| Tissue Development | Organization of extracellular matrix | 1.78E-05 |  |  | ADAM12,ITGAX,MMP12,MMP13,MMP19,SPP1,VCAN | 7 |
| Cell-To-Cell Signaling and Interaction | Binding of melanoma cell lines | 2.01E-05 |  |  | CD80,SELP,SPP1,VCAN | 4 |
| Cancer,Inflammatory Response,Organismal Injury and Abnormalities,Tumor Morphology | Rejection of tumor | 0.000021 |  |  | CD80,CTLA4,IL2RG | 3 |
| Cellular Function and Maintenance | Function of antigen presenting cells | 2.37E-05 |  |  | BIRC3,IL2RG,MMP12,MSR1,NCEH1,SPP1,TYROBP | 7 |
| Cancer,Hematological Disease,Immunological Disease,Organismal Injury and Abnormalities | Advanced-stage typical follicular lymphoma | 2.46E-05 |  |  | IRF8,KLHL6,P2RY8 | 3 |
| Inflammatory Disease,Organismal Injury and Abnormalities | Granuloma | 2.67E-05 |  |  | CD69,CD80,CTLA4,IRF8,MSR1,SPP1 | 6 |
| Cellular Function and Maintenance,Hematological System Development and Function | Function of macrophages | 2.89E-05 |  |  | BIRC3,MMP12,MSR1,NCEH1,SPP1,TYROBP | 6 |
| Cellular Function and Maintenance | Regulation of cells | 2.96E-05 |  |  | CD80,CTLA4,FNDC3B,SPP1,TYROBP | 5 |
| Immunological Disease,Organismal Injury and Abnormalities | Abnormality of thymus gland | 3.05E-05 |  |  | CTLA4,IL2RG,PTPN22,RAC2,RHOH,SPP1 | 6 |
| Inflammatory Response | Rejection | 3.19E-05 |  |  | CD80,CTLA4,GABRA4,IL2RG,MMP12 | 5 |
| Gastrointestinal Disease,Immunological Disease,Ophthalmic Disease,Organismal Injury and Abnormalities | Sjögren syndrome | 3.19E-05 |  |  | CD69,CXCL13,HP,SPP1,XCL1 | 5 |
| Endocrine System Disorders,Immunological Disease,Inflammatory Disease,Organismal Injury and Abnormalities | Autoimmune thyroiditis | 3.32E-05 |  |  | CD80,CTLA4,PTPN22 | 3 |
| Cancer,Organismal Injury and Abnormalities,Reproductive System Disease | Breast or ovarian cancer | 3.51E-05 |  |  | ADAM12,ADAMDEC1,BCAT1,CCDC71L,CCNA1,CD69,CD80,CTLA4,CYTH4,DMXL2,FNDC3B,GASK1B,HP,IL2RG,ITGAX,KLHL6,LOXL2,LY86,MMP12,MMP13,MMP19,MSR1,NRP2,PAPSS2,PTHLH,SELP,SH2B3,SIRPB1,SPP1,VCAN | 30 |
| Cancer,Hematological Disease,Immunological Disease,Organismal Injury and Abnormalities | Limited-stage typical follicular lymphoma | 0.000038 |  |  | IRF8,KLHL6,P2RY8 | 3 |
| Cell Death and Survival | Killing of macrophages | 0.000038 |  |  | CD84,MSR1,SLAMF7 | 3 |
| Cancer,Gastrointestinal Disease,Organismal Injury and Abnormalities | Digestive system cancer | 3.83E-05 |  |  | ADAM12,ADAMDEC1,ARHGAP9,BCAT1,BIRC3,CBLN2,CCNA1,CD37,CD69,CD84,CLEC4A,CTLA4,CXCL13,CYTH4,DMXL2,EVI2B,FERMT3,FNDC3B,GABRA4,GASK1B,GLIPR1,GLIPR2,GPRIN3,HLA-DPA1,HP,IL18BP,IL2RG,INA,IRF8,ITGAX,KLHL6,LOXL2,LPXN,MMP12,MMP13,MMP19,MSR1,NCEH1,NRP2,P2RY10,P2RY8,PAPSS2,PARVG,PTHLH,PTPN22,PTPRO,RHOH,SELP,SELPLG,SH2B3,SIRPB1,SLAMF7,SLC6A14,SNAP25,SPP1,TYROBP,VCAN,XCL1 | 58 |
| Cell-To-Cell Signaling and Interaction | Adhesion of lymphoma cell lines | 4.34E-05 |  |  | CXCL13,ITGAX,PARVG,VCAN | 4 |
| Cell Morphology | Morphology of leukocytes | 0.000044 |  |  | CD80,CD84,CTLA4,IL2RG,IRF8,PTPN22,RHOH,SPP1 | 8 |
| Embryonic Development,Hematological System Development and Function,Humoral Immune Response,Lymphoid Tissue Structure and Development,Organ Development,Organismal Development,Tissue Development | Formation of germinal center | 4.59E-05 |  |  | CD80,CTLA4,CXCL13,KLHL6 | 4 |
| Organismal Development,Organismal Injury and Abnormalities | Abnormal morphology of abdomen | 4.61E-05 |  |  | CD80,CTLA4,GLIS3,HP,IL2RG,IRF8,KLHL6,MSR1,PTPN22,PTPRO,RHOH,SELP,SH2B3,SPP1 | 14 |
| Endocrine System Disorders,Gastrointestinal Disease,Immunological Disease,Metabolic Disease,Organismal Injury and Abnormalities | Susceptibility to insulin-dependent diabetes mellitus | 4.82E-05 |  |  | CTLA4,PTPN22 | 2 |
| Cell-To-Cell Signaling and Interaction,Hematological System Development and Function | Interaction of monocytes | 4.82E-05 |  |  | CD80,CTLA4 | 2 |
| Cellular Movement,Hematological System Development and Function,Immune Cell Trafficking,Inflammatory Response | Cell movement of monocytes | 4.83E-05 |  |  | HP,ITGAX,MMP12,PTPRO,RAC2,SPP1 | 6 |
| Cellular Function and Maintenance,Hematological System Development and Function | Function of CD4+ T-lymphocytes | 4.86E-05 |  |  | CD69,CD80,CD84,PTPN22 | 4 |
| Connective Tissue Development and Function,Skeletal and Muscular System Development and Function,Tissue Development | Morphology of trabecular bone | 0.000052 |  |  | CLEC4A,MMP13,PTHLH,RAC2,SPP1,TYROBP | 6 |
| Cell Cycle,Hematological System Development and Function | Cell division of T lymphocytes | 5.55E-05 |  |  | CD37,CD80,CTLA4 | 3 |
| Hematological System Development and Function,Humoral Immune Response,Lymphoid Tissue Structure and Development,Tissue Morphology | Quantity of plasma cells | 6.05E-05 |  |  | IL2RG,IRF8,RAC2,SELP | 4 |
| Hematological System Development and Function,Lymphoid Tissue Structure and Development,Tissue Morphology | Abnormal quantity of lymphocytes | 6.38E-05 |  |  | CD80,IL2RG,IRF8,RHOH | 4 |
| Cancer,Hematological Disease,Immunological Disease,Organismal Injury and Abnormalities | B-cell neoplasm | 6.69E-05 |  |  | BCAT1,BIRC3,CD69,CD80,CLEC4A,CXCL13,GLIS3,IL2RG,IRF8,ITGAX,KLHL6,P2RY8,RHOH,SH2B3,SLAMF7,SPP1,VCAN | 17 |
| Cancer,Hematological Disease,Immunological Disease,Organismal Injury and Abnormalities | Chronic leukemia | 6.77E-05 |  |  | BCAT1,BIRC3,CD80,GABRA4,IL2RG,IRF8,ITGAX,MSR1,RHOH,VCAN | 10 |
| Immunological Disease,Inflammatory Disease,Organismal Injury and Abnormalities | Formation of granuloma | 6.97E-05 |  |  | CTLA4,IRF8,SPP1 | 3 |
| Cancer,Hematological Disease,Immunological Disease,Organismal Injury and Abnormalities | Mature B-cell neoplasm | 7.55E-05 |  |  | BCAT1,BIRC3,CD69,CD80,CLEC4A,CXCL13,GLIS3,IL2RG,IRF8,ITGAX,KLHL6,P2RY8,RHOH,SLAMF7,SPP1,VCAN | 16 |
| Cancer,Dermatological Diseases and Conditions,Organismal Injury and Abnormalities | Skin squamous cell carcinoma | 7.72E-05 |  |  | ADAM12,CD84,CTLA4,CYTH4,LOXL2,MSR1,P2RY10,PTPN22,PTPRO,RHOH,SNAP25,VCAN | 12 |
| Cellular Function and Maintenance,Cellular Movement,Hematological System Development and Function,Immune Cell Trafficking,Inflammatory Response,Lymphoid Tissue Structure and Development | Relocalization of lymphocytes | 8.02E-05 |  |  | CD69,CXCL13 | 2 |
| Cell Morphology,Humoral Immune Response,Immunological Disease,Lymphoid Tissue Structure and Development | Abnormal morphology of long-lived plasma cell | 8.02E-05 |  |  | CD80,CD84 | 2 |
| Cancer,Organismal Injury and Abnormalities | Delay in growth of malignant tumor | 8.02E-05 |  |  | CD80,CTLA4 | 2 |
| Hematological Disease,Immunological Disease,Inflammatory Disease | Eosinophilic inflammation | 8.25E-05 |  |  | ADAM12,ADAMDEC1,CD69,MMP12,SPP1 | 5 |
| Cellular Movement,Hematological System Development and Function,Immune Cell Trafficking,Inflammatory Response | Cell rolling of neutrophils | 0.000086 |  |  | RAC2,SELP,SELPLG | 3 |
| Cell-To-Cell Signaling and Interaction,Hematopoiesis | Response of hematopoietic progenitor cells | 0.000086 |  |  | IRF8,MSR1,SH2B3 | 3 |
| Cellular Compromise,Inflammatory Response | Degranulation of neutrophils | 8.77E-05 |  |  | ARHGAP9,GLIPR1,HP,ITGAX,PTPN22,SIRPB1,SNAP25,TYROBP | 8 |
| Gastrointestinal Disease,Inflammatory Response,Organismal Injury and Abnormalities | Inflammation of salivary gland | 0.000095 |  |  | CLEC4A,CTLA4,XCL1 | 3 |
| Cancer,Organismal Injury and Abnormalities | Abdominal adenocarcinoma | 9.77E-05 |  |  | ADAM12,ADAMDEC1,BCAT1,BIRC3,CBLN2,CCNA1,CD37,CD69,CD80,CD84,CLEC4A,CTLA4,CYTH4,DMXL2,EVI2B,FERMT3,FNDC3B,GABRA4,GASK1B,GLIPR1,GLIPR2,GPRIN3,IL18BP,IL2RG,INA,IRF8,ITGAX,KLHL6,LOXL2,LPXN,LY86,MMP13,MMP19,MSR1,NCEH1,NRP2,P2RY10,P2RY8,PAPSS2,PARVG,PTHLH,PTPN22,PTPRO,PXMP4,RAC2,RHOH,SELP,SELPLG,SH2B3,SIRPB1,SLAMF7,SLC6A14,SNAP25,SPP1,VCAN,XCL1 | 56 |
| Connective Tissue Disorders,Developmental Disorder,Hereditary Disorder,Organismal Injury and Abnormalities,Skeletal and Muscular Disorders | Chondrodysplasia | 9.93E-05 |  |  | MMP13,PAPSS2,PTHLH,TYROBP | 4 |
| Antimicrobial Response,Inflammatory Response | Antimicrobial response | 0.000106 |  |  | BIRC3,CXCL13,IRF8,ITGAX,MMP12,MSR1,PTPN22,XCL1 | 8 |
| Connective Tissue Development and Function,Connective Tissue Disorders,Organ Morphology,Organismal Development,Organismal Injury and Abnormalities,Skeletal and Muscular Disorders,Skeletal and Muscular System Development and Function,Tissue Development | Abnormal morphology of femur | 0.000109 |  |  | CD80,MMP13,PTHLH,TYROBP | 4 |
| Cell Death and Survival | Cytolysis | 0.000116 |  |  | CD69,CTLA4,IL2RG,SLAMF7,SPP1,TYROBP,XCL1 | 7 |
| Cardiovascular Disease,Organismal Injury and Abnormalities | Aortic aneurysm | 0.000119 |  |  | MMP12,MMP13,SPP1,VCAN | 4 |
| Cancer,Organismal Injury and Abnormalities,Respiratory Disease | TNM stage T2 laryngeal squamous cell carcinoma | 0.00012 |  |  | CTLA4,SPP1 | 2 |
| Organ Morphology,Tissue Morphology | Enlargement of alveolar lumen | 0.00012 |  |  | MMP12,SPP1 | 2 |
| Cancer,Inflammatory Response,Organismal Injury and Abnormalities,Tumor Morphology | Rejection of malignant tumor | 0.00012 |  |  | CTLA4,IL2RG | 2 |
| Cardiovascular Disease | Vascular lesion | 0.000123 |  |  | CTLA4,MMP12,MMP13,MSR1,SELP,SPP1,VCAN | 7 |
| Cell Morphology,Humoral Immune Response,Immunological Disease,Lymphoid Tissue Structure and Development | Abnormal morphology of B lymphocytes | 0.00013 |  |  | CD80,CD84,IL2RG,IRF8 | 4 |
| Organismal Injury and Abnormalities,Tissue Morphology | Size of lesion | 0.000131 |  |  | ADAM12,CTLA4,IL18BP,MMP12,MSR1,PTHLH,SELP,SPP1 | 8 |
| Cardiovascular Disease,Organismal Injury and Abnormalities | Intermediate disease stage peripheral arterial disease | 0.000142 |  |  | CLEC4A,EVI2B,IRF8,LPXN,SPP1 | 5 |
| Cellular Function and Maintenance,Cellular Movement | Localization of blood cells | 0.000149 |  |  | CD69,CXCL13,SELP | 3 |
| Cellular Development,Cellular Growth and Proliferation,Hematological System Development and Function,Hematopoiesis,Lymphoid Tissue Structure and Development,Tissue Development | Granulopoiesis | 0.000158 |  |  | EVI2B,IRF8,RAC2,SELP,SH2B3 | 5 |
| Cell Death and Survival | Cytolysis of lymphocytes | 0.00016 |  |  | IL2RG,SLAMF7,TYROBP,XCL1 | 4 |
| Cell-To-Cell Signaling and Interaction,Hematological System Development and Function,Inflammatory Response | Binding of blood platelets | 0.00016 |  |  | CD84,FERMT3,SELP,SELPLG | 4 |
| Cell Death and Survival | Killing of bone marrow-derived macrophages | 0.000168 |  |  | CD84,SLAMF7 | 2 |
| Cancer,Organismal Injury and Abnormalities,Respiratory Disease | TNM stage T1 laryngeal squamous cell carcinoma | 0.000168 |  |  | CTLA4,SPP1 | 2 |
| Endocrine System Disorders,Gastrointestinal Disease,Metabolic Disease,Organismal Injury and Abnormalities | Severe diabetes mellitus | 0.000168 |  |  | CD80,GLIS3 | 2 |
| Cancer,Organismal Injury and Abnormalities,Respiratory Disease | TNM stage T3 laryngeal squamous cell carcinoma | 0.000168 |  |  | CTLA4,SPP1 | 2 |
| Skeletal and Muscular Disorders,Tissue Morphology | Degradation of cartilage matrix | 0.000168 |  |  | MMP13,PTHLH | 2 |
| Cell-mediated Immune Response,Cellular Movement,Hematological System Development and Function,Immune Cell Trafficking | Cell rolling of Th1 cells | 0.000168 |  |  | SELP,SELPLG | 2 |
| Hematological System Development and Function,Immunological Disease,Lymphoid Tissue Structure and Development,Organ Morphology,Organismal Development,Organismal Injury and Abnormalities,Tissue Morphology | Abnormal splenic cell ratio | 0.000176 |  |  | CTLA4,IL2RG,RHOH | 3 |
| Embryonic Development,Hematological System Development and Function,Humoral Immune Response,Lymphoid Tissue Structure and Development,Organ Development,Organismal Development,Tissue Development,Tissue Morphology | Size of germinal center | 0.000176 |  |  | CD37,CD84,TYROBP | 3 |
| Connective Tissue Development and Function,Connective Tissue Disorders,Organismal Injury and Abnormalities,Skeletal and Muscular Disorders,Skeletal and Muscular System Development and Function,Tissue Development | Abnormal morphology of trabecular bone | 0.000195 |  |  | MMP13,PTHLH,SPP1,TYROBP | 4 |
| Connective Tissue Disorders,Organismal Injury and Abnormalities,Skeletal and Muscular Disorders | Abnormal bone density | 0.000202 |  |  | CTLA4,FERMT3,GABRA4,IRF8,PTHLH,TYROBP | 6 |
| Connective Tissue Development and Function,Skeletal and Muscular System Development and Function,Tissue Development | Volume of trabecular bone | 0.000203 |  |  | CLEC4A,MMP13,PTHLH,TYROBP | 4 |
| Hematological System Development and Function,Immunological Disease,Inflammatory Disease,Lymphoid Tissue Structure and Development,Organ Morphology,Organismal Development,Organismal Injury and Abnormalities,Tissue Morphology | Enlargement of spleen | 0.000213 |  |  | CTLA4,HP,IL2RG,IRF8,PTPN22,SH2B3 | 6 |
| Antimicrobial Response,Inflammatory Response | Antiviral response | 0.000213 |  |  | BIRC3,ITGAX,MMP12,MSR1,PTPN22,XCL1 | 6 |
| Endocrine System Disorders,Organismal Injury and Abnormalities | Benign thyroid disease | 0.000214 |  |  | CD80,CTLA4,GLIS3,PTPN22,SNAP25 | 5 |
| Cancer,Hematological Disease,Organismal Injury and Abnormalities | Chronic myeloproliferative neoplasm | 0.000217 |  |  | BCAT1,GABRA4,IL2RG,IRF8,MSR1,SH2B3 | 6 |
| Cell-To-Cell Signaling and Interaction,Hematological System Development and Function,Immune Cell Trafficking,Inflammatory Response | Adhesion of bone marrow-derived neutrophils | 0.000223 |  |  | FERMT3,PTPN22 | 2 |
| Cell-mediated Immune Response,Cell-To-Cell Signaling and Interaction,Cellular Movement,Hematological System Development and Function,Immune Cell Trafficking | Adhesion of Th1 cells | 0.000223 |  |  | SELP,SELPLG | 2 |
| Embryonic Development,Organismal Development,Tissue Morphology | Length of embryo | 0.000223 |  |  | NRP2,PTHLH | 2 |
| Cell-To-Cell Signaling and Interaction,Renal and Urological System Development and Function | Binding of kidney cell lines | 0.000244 |  |  | CD80,IL2RG,MSR1,SELPLG | 4 |
| Cellular Development,Cellular Growth and Proliferation,Hematological System Development and Function,Lymphoid Tissue Structure and Development | Proliferation of naive T lymphocytes | 0.000255 |  |  | CD69,CD80,CTLA4 | 3 |
| Cancer,Organismal Injury and Abnormalities | Cancer of secretory structure | 0.000269 |  |  | ADAM12,ADAMDEC1,ARHGAP9,BCAT1,BIRC3,CBLN2,CCNA1,CD37,CD69,CD80,CLEC4A,CTLA4,DMXL2,EVI2B,FERMT3,FNDC3B,GABRA4,GASK1B,GLIPR1,GLIPR2,GLIS3,GPRIN3,HLA-DPA1,HP,IL18BP,IL2RG,INA,IRF8,ITGAX,KLHL6,LOXL2,LPXN,MMP13,MMP19,MSR1,NCEH1,NRP2,P2RY8,PAPSS2,PARVG,PTHLH,PTPN22,PTPRO,PXMP4,RAC2,SELP,SELPLG,SH2B3,SIRPB1,SLC6A14,SNAP25,SPP1,TYROBP,VCAN,XCL1 | 55 |
| Cancer,Hematological Disease,Immunological Disease,Organismal Injury and Abnormalities | T-cell malignant neoplasm | 0.000271 |  |  | CD69,CXCL13,FNDC3B,IL2RG,ITGAX,MMP12,RHOH,SELP,SELPLG,SH2B3 | 10 |
| Inflammatory Disease,Ophthalmic Disease,Organismal Injury and Abnormalities | Uveitis | 0.000271 |  |  | CTLA4,GABRA4,IRF8,SPP1 | 4 |
| Cancer,Hematological Disease,Immunological Disease,Organismal Injury and Abnormalities | Follicular lymphoma | 0.000271 |  |  | IL2RG,IRF8,KLHL6,P2RY8 | 4 |
| Cell-To-Cell Signaling and Interaction,Cellular Assembly and Organization,Hematological System Development and Function,Immune Cell Trafficking | Cell-cell contact of leukocytes | 0.000273 |  |  | CD80,FERMT3,SELP | 3 |
| Inflammatory Response | Acute inflammation of tissue | 0.000273 |  |  | CTLA4,SELP,SPP1 | 3 |
| Cellular Movement | Invasion of bone cancer cell lines | 0.000273 |  |  | MMP12,MMP13,VCAN | 3 |
| Cardiovascular System Development and Function,Connective Tissue Development and Function,Organismal Development,Tissue Development | Vascularization of bone | 0.000287 |  |  | MMP13,PTHLH | 2 |
| Immunological Disease | Immunodeficiency | 0.000302 |  |  | CTLA4,FERMT3,IL2RG,IRF8,RAC2,RHOH | 6 |
| Cell Morphology,Cellular Assembly and Organization,Cellular Function and Maintenance | Formation of lamellipodia | 0.000304 |  |  | CTLA4,FERMT3,PTPRO,RAC2,SIRPB1 | 5 |
| Cancer,Organismal Injury and Abnormalities | Squamous-cell carcinoma | 0.000308 |  |  | ADAM12,CD84,CTLA4,CXCL13,CYTH4,DMXL2,GASK1B,INA,IRF8,ITGAX,LOXL2,MMP12,MMP13,MSR1,NRP2,P2RY10,P2RY8,PAPSS2,PTHLH,PTPN22,PTPRO,RHOH,SELPLG,SIRPB1,SNAP25,SPP1,VCAN | 27 |
| Hematological System Development and Function,Tissue Morphology | Morphology of bone marrow | 0.000322 |  |  | IRF8,PTHLH,SELP,SH2B3 | 4 |
| Cell-To-Cell Signaling and Interaction | Adhesion of leukemia cell lines | 0.000333 |  |  | FERMT3,SELP,SELPLG,SPP1 | 4 |
| Cell-To-Cell Signaling and Interaction | Response of granulocytes | 0.000333 |  |  | CTLA4,ITGAX,SELPLG,TYROBP | 4 |
| Cellular Movement,Hematological System Development and Function,Humoral Immune Response,Immune Cell Trafficking | Cell movement of pre-B lymphocytes | 0.000358 |  |  | CXCL13,SELPLG | 2 |
| Connective Tissue Disorders,Organismal Injury and Abnormalities | Damage of connective tissue | 0.000367 |  |  | CTLA4,MMP13,SPP1,VCAN | 4 |
| Endocrine System Disorders,Metabolic Disease,Organismal Injury and Abnormalities | Hyperthyroidism | 0.000375 |  |  | GLIS3,PTPN22,SNAP25 | 3 |
| Cardiovascular Disease,Organismal Injury and Abnormalities | Atherosclerotic lesion | 0.000386 |  |  | MMP12,MSR1,SELP,SPP1,VCAN | 5 |
| Cancer,Hematological Disease,Immunological Disease,Organismal Injury and Abnormalities | Chronic myeloid leukemia | 0.000394 |  |  | BCAT1,GABRA4,IL2RG,IRF8,MSR1 | 5 |
| Amino Acid Metabolism,Small Molecule Biochemistry | Synthesis of L-glutamic acid | 0.000436 |  |  | BCAT1,VCAN | 2 |
| Cellular Movement | Cell rolling of leukemia cell lines | 0.000436 |  |  | SELP,SELPLG | 2 |
| Gastrointestinal Disease,Organismal Injury and Abnormalities | Benign oral disorder | 0.000477 |  |  | CD69,CXCL13,GABRA4,HP,MMP12,SNAP25,SPP1,XCL1 | 8 |
| Cancer,Organismal Injury and Abnormalities,Tissue Morphology,Tumor Morphology | Volume of malignant tumor | 0.000523 |  |  | CTLA4,IL18BP | 2 |
| Cellular Development,Renal and Urological System Development and Function | Differentiation of podocytes | 0.000523 |  |  | PTPRO,SPP1 | 2 |
| Cellular Function and Maintenance,Hematological System Development and Function | Regulation of leukocytes | 0.000528 |  |  | CD80,CTLA4,SPP1 | 3 |
| Cardiovascular Disease,Organismal Injury and Abnormalities | Peripheral vascular disease | 0.000544 |  |  | CLEC4A,EVI2B,GABRA4,IRF8,LPXN,MSR1,SNAP25,SPP1 | 8 |
| Cancer,Organismal Injury and Abnormalities | Extrapulmonary squamous cell carcinoma | 0.000554 |  |  | ADAM12,CD84,CTLA4,CXCL13,CYTH4,DMXL2,GASK1B,INA,IRF8,ITGAX,LOXL2,MMP12,MMP13,MSR1,NRP2,P2RY10,P2RY8,PAPSS2,PTPN22,PTPRO,RHOH,SIRPB1,SNAP25,SPP1,VCAN | 25 |
| Cell-To-Cell Signaling and Interaction,Connective Tissue Development and Function | Activation of fibroblasts | 0.000557 |  |  | LOXL2,PTHLH,SPP1 | 3 |
| Cancer,Organismal Injury and Abnormalities,Reproductive System Disease | Breast cancer | 0.000563 |  |  | ADAM12,BCAT1,CCDC71L,CD69,CD80,CTLA4,CYTH4,DMXL2,GASK1B,HP,IL2RG,ITGAX,KLHL6,LOXL2,MMP12,MMP13,MMP19,NRP2,PTHLH,SPP1,VCAN | 21 |
| Cancer,Hematological Disease,Immunological Disease,Organismal Injury and Abnormalities | B cell cancer | 0.000595 |  |  | BCAT1,BIRC3,CD69,CD80,CLEC4A,CXCL13,GLIS3,IL2RG,IRF8,KLHL6,P2RY8,SH2B3,SLAMF7,SPP1 | 14 |
| Gastrointestinal Disease,Organismal Injury and Abnormalities | Colorectal disorder | 0.000611 |  |  | ADAM12,CCNA1,CD37,CD69,CD80,CTLA4,CYTH4,DMXL2,FNDC3B,GABRA4,GASK1B,GLIPR2,IL2RG,IRF8,ITGAX,KLHL6,LOXL2,LPXN,MMP12,MMP13,MMP19,NCEH1,NRP2,P2RY10,P2RY8,PAPSS2,PARVG,PTHLH,PTPN22,SELP,SH2B3,SLAMF7,SLC6A14,SPP1,VCAN,XCL1 | 36 |
| Cancer,Organismal Injury and Abnormalities,Reproductive System Disease,Tumor Morphology | Progression of mammary tumor | 0.000617 |  |  | ADAM12,CTLA4 | 2 |
| Cell-To-Cell Signaling and Interaction,Hematological System Development and Function,Immune Cell Trafficking,Inflammatory Response | Priming of T lymphocytes | 0.000617 |  |  | CD80,CTLA4,IRF8 | 3 |
| Connective Tissue Development and Function,Connective Tissue Disorders,Organ Morphology,Organismal Development,Organismal Injury and Abnormalities,Skeletal and Muscular Disorders,Skeletal and Muscular System Development and Function,Tissue Development | Abnormal morphology of short femur | 0.000617 |  |  | MMP13,PTHLH,TYROBP | 3 |
| Cell Death and Survival | Cytolysis of natural killer cells | 0.000649 |  |  | IL2RG,SLAMF7,TYROBP | 3 |
| Hematological System Development and Function,Hematopoiesis,Lymphoid Tissue Structure and Development,Tissue Morphology | Quantity of megakaryocytes | 0.000649 |  |  | IL2RG,SELP,SH2B3 | 3 |
| Connective Tissue Disorders,Developmental Disorder,Hereditary Disorder,Organismal Injury and Abnormalities,Skeletal and Muscular Disorders | Osteochondrodysplasia | 0.000649 |  |  | MMP13,PAPSS2,TYROBP | 3 |
| Cellular Movement,Hematological System Development and Function,Immune Cell Trafficking,Inflammatory Response | Migration of dendritic cells | 0.000681 |  |  | CD80,CXCL13,NRP2,SPP1 | 4 |
| Cell-To-Cell Signaling and Interaction,Hematological System Development and Function,Immune Cell Trafficking,Inflammatory Response | Aggregation of leukocytes | 0.000682 |  |  | P2RY8,SELP,SELPLG | 3 |
| Gastrointestinal Disease,Inflammatory Disease | Crohn disease | 0.000698 |  |  | ADAM12,BIRC3,CD80,GABRA4,SELP,SPP1 | 6 |
| Cancer,Organismal Injury and Abnormalities | Breast or pancreatic cancer | 0.0007 |  |  | ADAM12,ADAMDEC1,BCAT1,CCDC71L,CCNA1,CD69,CD80,CTLA4,CYTH4,DMXL2,EVI2B,FNDC3B,GABRA4,GASK1B,HP,IL18BP,IL2RG,ITGAX,KLHL6,LOXL2,LY86,MMP12,MMP13,MMP19,MSR1,NRP2,PAPSS2,PTHLH,SELP,SH2B3,SIRPB1,SPP1,VCAN | 33 |
| Cancer,Dermatological Diseases and Conditions,Organismal Injury and Abnormalities | Skin carcinoma | 0.000706 |  |  | ADAM12,CCNA1,CD84,CTLA4,CYTH4,GABRA4,LOXL2,MSR1,P2RY10,PTPN22,PTPRO,RHOH,SNAP25,VCAN | 14 |
| Cellular Function and Maintenance | Regulation of bone cells | 0.000718 |  |  | FNDC3B,TYROBP | 2 |
| Organismal Injury and Abnormalities,Renal and Urological Disease | Phosphaturia | 0.000718 |  |  | PTHLH,SPP1 | 2 |
| Cell Morphology,Hematological System Development and Function | Morphology of myeloid cells | 0.000736 |  |  | IL2RG,IRF8,SELP,SH2B3,SPP1 | 5 |
| Skeletal and Muscular Disorders | Abnormality of lower limb | 0.000749 |  |  | CD80,MMP13,PTHLH,SPP1,TYROBP | 5 |
| Cell Morphology,Hematological System Development and Function,Immunological Disease,Lymphoid Tissue Structure and Development | Abnormal morphology of T lymphocytes | 0.000758 |  |  | CTLA4,IL2RG,PTPN22,RHOH | 4 |
| Cancer,Hematological Disease,Organismal Injury and Abnormalities | Mature T-cell or NK-cell neoplasm | 0.000824 |  |  | CD69,CXCL13,FNDC3B,IL2RG,ITGAX,MMP12,SELP,SELPLG | 8 |
| Cell Morphology,Hematological Disease,Hematological System Development and Function,Hematopoiesis,Lymphoid Tissue Structure and Development,Tissue Morphology | Abnormal morphology of megakaryocytes | 0.000827 |  |  | IRF8,SH2B3 | 2 |
| Cell-To-Cell Signaling and Interaction,Hematological System Development and Function,Immune Cell Trafficking,Inflammatory Response | Aggregation of granulocytes | 0.000827 |  |  | SELP,SELPLG | 2 |
| Cancer,Hematological Disease,Immunological Disease,Organismal Injury and Abnormalities | Mature B-cell lymphoma | 0.000847 |  |  | BCAT1,BIRC3,CD69,CXCL13,IL2RG,IRF8,KLHL6,P2RY8 | 8 |
| Hematological System Development and Function,Tissue Morphology | Abnormal number of leukocytes | 0.000861 |  |  | CD80,IL2RG,IRF8 | 3 |
| Gastrointestinal Disease,Inflammatory Disease,Inflammatory Response,Organismal Injury and Abnormalities | Colitis | 0.000893 |  |  | CD69,CD80,CTLA4,GABRA4,PTPN22,SELP,SPP1 | 7 |
| Hematological System Development and Function | Coagulation | 0.000903 |  |  | HP,PAPSS2,SELP,SELPLG,SH2B3,VCAN | 6 |
| Cellular Development,Hematological System Development and Function,Lymphoid Tissue Structure and Development | Maturation of antigen presenting cells | 0.000906 |  |  | CD69,CD80,IRF8,TYROBP | 4 |
| Cancer,Hematological Disease,Immunological Disease,Organismal Injury and Abnormalities | Non-Hodgkin lymphoma | 0.000926 |  |  | BCAT1,BIRC3,CD69,CXCL13,GLIS3,IL2RG,IRF8,ITGAX,KLHL6,MMP12,P2RY8,SELP,SELPLG | 13 |
| Cardiovascular Disease,Organismal Injury and Abnormalities | Mitral valvular disease | 0.000941 |  |  | ADAM12,GABRA4,SPP1 | 3 |
| Gastrointestinal Disease,Organismal Injury and Abnormalities | Benign salivary gland disease | 0.000943 |  |  | CXCL13,SNAP25 | 2 |
| Cellular Development,Cellular Growth and Proliferation,Embryonic Development,Hematological System Development and Function,Hematopoiesis,Humoral Immune Response,Lymphoid Tissue Structure and Development,Organ Development,Organismal Development,Tissue Development | Development of plasma cells | 0.000943 |  |  | CD69,CD80 | 2 |
| Cancer,Hematological Disease,Immunological Disease,Organismal Injury and Abnormalities | Mature B cell malignant tumor | 0.000976 |  |  | BCAT1,BIRC3,CD69,CLEC4A,CXCL13,GLIS3,IL2RG,IRF8,KLHL6,P2RY8,SLAMF7,SPP1 | 12 |
| Cancer,Gastrointestinal Disease,Organismal Injury and Abnormalities | Gastrointestinal tract cancer | 0.00102 |  |  | ADAM12,ADAMDEC1,BIRC3,CBLN2,CCNA1,CD37,CD69,CD84,CLEC4A,CTLA4,CXCL13,CYTH4,DMXL2,FERMT3,FNDC3B,GABRA4,GASK1B,GLIPR1,GLIPR2,IL18BP,IL2RG,INA,IRF8,ITGAX,KLHL6,LOXL2,LPXN,MMP12,MMP13,MMP19,MSR1,NCEH1,NRP2,P2RY10,P2RY8,PAPSS2,PARVG,PTHLH,PTPN22,PTPRO,RHOH,SELP,SELPLG,SH2B3,SIRPB1,SLAMF7,SLC6A14,SNAP25,SPP1,TYROBP,VCAN,XCL1 | 52 |
| Cell-To-Cell Signaling and Interaction | Binding of cell surface | 0.00102 |  |  | CXCL13,MSR1,XCL1 | 3 |
| Cell-To-Cell Signaling and Interaction,Connective Tissue Development and Function,Skeletal and Muscular System Development and Function,Tissue Development | Activation of osteoclasts | 0.00102 |  |  | PTHLH,SPP1,TYROBP | 3 |
| Cancer,Organismal Injury and Abnormalities | Cancer of head | 0.00103 |  |  | ADAM12,CTLA4,CXCL13,CYTH4,DMXL2,ITGAX,LOXL2,MMP12,MSR1,PTPRO,SH2B3,SNAP25,SPP1,TYROBP | 14 |
| Cell Cycle,Hematological System Development and Function | Entry into cell division of T lymphocytes | 0.00107 |  |  | CD37,CTLA4 | 2 |
| Infectious Diseases | Multidrug-resistant tuberculosis | 0.00107 |  |  | GABRA4,IL2RG | 2 |
| Cell Morphology,Hematological Disease,Hematological System Development and Function,Hematopoiesis,Lymphoid Tissue Structure and Development,Tissue Morphology | Abnormal morphology of bone marrow cells | 0.00111 |  |  | IRF8,SELP,SH2B3 | 3 |
| Cancer,Dermatological Diseases and Conditions,Hematological Disease,Immunological Disease,Inflammatory Disease,Inflammatory Response,Organismal Injury and Abnormalities | Mycosis fungoides | 0.00111 |  |  | CXCL13,IL2RG,SELPLG | 3 |
| Gastrointestinal Disease,Hematological Disease,Immunological Disease,Inflammatory Disease,Inflammatory Response,Organismal Injury and Abnormalities | Eosinophilia of esophagus | 0.00111 |  |  | ADAMDEC1,MMP12,SPP1 | 3 |
| Connective Tissue Disorders,Organismal Injury and Abnormalities,Skeletal and Muscular Disorders | Osteoporosis | 0.00112 |  |  | CTLA4,GABRA4,IRF8,PTHLH | 4 |
| Cellular Development,Cellular Growth and Proliferation,Embryonic Development,Hematological System Development and Function,Hematopoiesis,Lymphoid Tissue Structure and Development,Organ Development,Organismal Development,Tissue Development | Arrest in differentiation of lymphocytes | 0.00116 |  |  | IL2RG,IRF8,RHOH | 3 |
| Cardiovascular Disease,Organismal Injury and Abnormalities | Abnormality of atrium | 0.00118 |  |  | ADAM12,BIRC3,GABRA4,SPP1 | 4 |
| Cell-To-Cell Signaling and Interaction,Hematological System Development and Function,Immune Cell Trafficking,Inflammatory Response | Activation of naive T lymphocytes | 0.0012 |  |  | CD80,CTLA4 | 2 |
| Cell Death and Survival | Recovery of blood cells | 0.0012 |  |  | MMP12,SH2B3 | 2 |
| Cell Cycle,Skeletal and Muscular System Development and Function | Cell cycle progression of muscle cell lines | 0.0012 |  |  | ADAM12,PTHLH | 2 |
| Cellular Growth and Proliferation,Hematological System Development and Function,Lymphoid Tissue Structure and Development,Organ Development,Tissue Development | Proliferation of lymph node cells | 0.0012 |  |  | CD80,CTLA4 | 2 |
| Cell-To-Cell Signaling and Interaction,Cellular Movement,Immune Cell Trafficking | Attraction of leukocytes | 0.00121 |  |  | CXCL13,VCAN,XCL1 | 3 |
| Cancer,Gastrointestinal Disease,Organismal Injury and Abnormalities | Colorectal cancer | 0.00122 |  |  | ADAM12,CCNA1,CD37,CD69,CTLA4,CYTH4,DMXL2,FNDC3B,GASK1B,GLIPR2,IL2RG,IRF8,ITGAX,KLHL6,LOXL2,LPXN,MMP12,MMP13,MMP19,NCEH1,NRP2,P2RY10,P2RY8,PAPSS2,PARVG,PTHLH,PTPN22,SELP,SH2B3,SLAMF7,SLC6A14,SPP1,VCAN,XCL1 | 34 |
| Hematological System Development and Function,Immunological Disease,Lymphoid Tissue Structure and Development,Organ Morphology,Organismal Development,Organismal Injury and Abnormalities,Tissue Morphology | Abnormal morphology of thymus gland | 0.00123 |  |  | CTLA4,IL2RG,PTPN22,RHOH | 4 |
| Developmental Disorder | Disorder of stature | 0.00124 |  |  | MMP13,PAPSS2,PTHLH,SNAP25,TYROBP | 5 |
| Tissue Development | Disassembly of extracellular matrix | 0.00126 |  |  | MMP12,MMP13,MMP19 | 3 |
| Hematological System Development and Function,Hematopoiesis,Lymphoid Tissue Structure and Development,Organ Morphology,Tissue Morphology | Quantity of Single positive thymocytes | 0.0013 |  |  | CLEC4A,IL2RG,RHOH | 3 |
| Cancer,Organismal Injury and Abnormalities | Sun-shielded melanoma | 0.00132 |  |  | CTLA4,IL2RG,PARVG,SPP1 | 4 |
| Cancer,Organismal Injury and Abnormalities | Pelvic tumor | 0.00132 |  |  | ADAM12,ADAMDEC1,BCAT1,BIRC3,CCNA1,CD37,CD69,CD80,CTLA4,CYTH4,DMXL2,FERMT3,FNDC3B,GABRA4,GASK1B,GLIPR1,GPRIN3,HP,IL2RG,INA,ITGAX,LOXL2,LY86,MMP12,MMP13,MMP19,MSR1,NRP2,PAPSS2,PARVG,PTPN22,PTPRO,PXMP4,RAC2,SELP,SELPLG,SH2B3,SIRPB1,SPP1,VCAN,XCL1 | 41 |
| Cell-To-Cell Signaling and Interaction,Hematological System Development and Function,Immune Cell Trafficking,Inflammatory Response | Activation of natural killer T lymphocytes | 0.00134 |  |  | CD80,SPP1 | 2 |
| Cellular Assembly and Organization,Inflammatory Response | Formation of neutrophil extracellular trap | 0.00134 |  |  | SELP,SELPLG | 2 |
| Cellular Development,Cellular Growth and Proliferation,Hematological System Development and Function,Lymphoid Tissue Structure and Development | Expansion of Th17 cells | 0.00134 |  |  | CD69,IRF8 | 2 |
| Cell-mediated Immune Response,Cellular Movement,Hematological System Development and Function,Immune Cell Trafficking | Movement of CD4+ T-lymphocytes | 0.00141 |  |  | CTLA4,RAC2,SPP1 | 3 |
| Digestive System Development and Function,Gastrointestinal Disease,Hepatic System Development and Function,Hepatic System Disease,Inflammatory Disease,Inflammatory Response,Organ Development,Organismal Injury and Abnormalities | Chronic hepatitis | 0.00141 |  |  | CTLA4,GABRA4,IL2RG,SPP1 | 4 |
| Cancer,Gastrointestinal Disease,Organismal Injury and Abnormalities | Malignant neoplasm of large intestine | 0.00143 |  |  | ADAM12,ADAMDEC1,BIRC3,CBLN2,CCNA1,CD37,CD69,CD84,CLEC4A,CTLA4,CYTH4,DMXL2,FERMT3,FNDC3B,GABRA4,GASK1B,GLIPR1,GLIPR2,IL18BP,IL2RG,INA,IRF8,ITGAX,KLHL6,LOXL2,LPXN,MMP12,MMP13,MMP19,MSR1,NCEH1,NRP2,P2RY10,P2RY8,PAPSS2,PARVG,PTHLH,PTPN22,PTPRO,RHOH,SELP,SELPLG,SH2B3,SIRPB1,SLAMF7,SLC6A14,SNAP25,SPP1,VCAN,XCL1 | 50 |
| Hematological System Development and Function,Hematopoiesis,Lymphoid Tissue Structure and Development,Organ Morphology,Tissue Morphology | Quantity of double-negative T lymphocyte | 0.00146 |  |  | CTLA4,IL2RG,RHOH | 3 |
| Cancer,Developmental Disorder,Hematological Disease,Immunological Disease,Organismal Injury and Abnormalities | Marginal zone cell lymphoma | 0.00146 |  |  | BIRC3,CD69,CXCL13 | 3 |
| Hematological System Development and Function,Lymphoid Tissue Structure and Development,Tissue Morphology | Quantity of effector memory T lymphocytes | 0.00148 |  |  | CTLA4,SELPLG | 2 |
| Cancer,Gastrointestinal Disease,Organismal Injury and Abnormalities | Gastrointestinal carcinoma | 0.00154 |  |  | ADAM12,ADAMDEC1,BIRC3,CBLN2,CCNA1,CD37,CD69,CD84,CLEC4A,CTLA4,CXCL13,CYTH4,DMXL2,FERMT3,FNDC3B,GABRA4,GLIPR1,GLIPR2,IL18BP,IL2RG,INA,IRF8,ITGAX,KLHL6,LOXL2,LPXN,MMP12,MMP13,MMP19,MSR1,NCEH1,NRP2,P2RY10,P2RY8,PAPSS2,PARVG,PTHLH,PTPN22,PTPRO,RHOH,SELP,SELPLG,SH2B3,SIRPB1,SLAMF7,SLC6A14,SNAP25,SPP1,TYROBP,VCAN | 50 |
| Cellular Movement,Hematological System Development and Function,Immune Cell Trafficking | Transmigration of leukocytes | 0.0016 |  |  | FERMT3,ITGAX,RAC2,SELPLG | 4 |
| Connective Tissue Disorders,Hereditary Disorder,Organismal Injury and Abnormalities | Hereditary connective tissue disorder | 0.00162 |  |  | CTLA4,FERMT3,IRF8,MMP13,PAPSS2,PTHLH,PTPN22,TYROBP,VCAN | 9 |
| Cell-To-Cell Signaling and Interaction,Connective Tissue Development and Function | Adhesion of fibroblast cell lines | 0.00163 |  |  | ADAM12,CD80,SPP1 | 3 |
| Cell-To-Cell Signaling and Interaction,Cellular Function and Maintenance,Hematopoiesis,Inflammatory Response | Phagocytosis of hematopoietic progenitor cells | 0.00164 |  |  | IRF8,MSR1 | 2 |
| Cell-To-Cell Signaling and Interaction,Cellular Function and Maintenance,Inflammatory Response | Phagocytosis of lymphatic system cells | 0.00164 |  |  | IRF8,MSR1 | 2 |
| Cellular Development,Cellular Growth and Proliferation,Hematological System Development and Function,Lymphoid Tissue Structure and Development | Expansion of TREG cells | 0.00164 |  |  | CTLA4,PTPN22 | 2 |
| Skeletal and Muscular Disorders | Torticollis | 0.00164 |  |  | SELP,SNAP25 | 2 |
| Connective Tissue Development and Function,Connective Tissue Disorders,Organismal Injury and Abnormalities,Skeletal and Muscular Disorders,Skeletal and Muscular System Development and Function,Tissue Development | Abnormal morphology of marrow space | 0.00164 |  |  | MMP13,TYROBP | 2 |
| Cellular Movement,Connective Tissue Development and Function | Migration of fibroblast cell lines | 0.00167 |  |  | MSR1,RAC2,SPP1,XCL1 | 4 |
| Hematological System Development and Function,Lymphoid Tissue Structure and Development,Tissue Morphology | Quantity of memory T lymphocytes | 0.00169 |  |  | CTLA4,PTPN22,SELPLG | 3 |
| Organismal Injury and Abnormalities | Non-cancer pain | 0.0017 |  |  | ARHGAP9,GABRA4,SNAP25,TYROBP | 4 |
| Developmental Disorder,Hereditary Disorder,Immunological Disease,Organismal Injury and Abnormalities | Hypoplasia of spleen | 0.00175 |  |  | IL2RG,KLHL6,RHOH | 3 |
| Organismal Injury and Abnormalities | Abnormality of peritoneum | 0.00175 |  |  | BIRC3,IL2RG,SELP | 3 |
| Cancer,Organismal Injury and Abnormalities | Breast or colorectal cancer | 0.00175 |  |  | ADAM12,BCAT1,CCDC71L,CCNA1,CD37,CD69,CD80,CTLA4,CYTH4,DMXL2,FNDC3B,GASK1B,GLIPR2,HP,IL2RG,IRF8,ITGAX,KLHL6,LOXL2,LPXN,MMP12,MMP13,MMP19,NCEH1,NRP2,P2RY10,P2RY8,PAPSS2,PARVG,PTHLH,PTPN22,SELP,SH2B3,SLAMF7,SLC6A14,SPP1,VCAN,XCL1 | 38 |
| Dermatological Diseases and Conditions,Organismal Injury and Abnormalities | Psoriasis | 0.00178 |  |  | ADAM12,ADAMDEC1,CTLA4,MMP12,MMP19,NRP2,SLAMF7,SLC6A14 | 8 |
| Protein Synthesis | Quantity of IL-4 in blood | 0.0018 |  |  | CLEC4A,SLC6A14 | 2 |
| Cell-To-Cell Signaling and Interaction | Adhesion of muscle cells | 0.0018 |  |  | ADAM12,SPP1 | 2 |
| Cancer,Organismal Injury and Abnormalities | Malignant genitourinary solid tumor | 0.00181 |  |  | ADAM12,ADAMDEC1,BCAT1,BIRC3,CCDC71L,CCNA1,CD37,CD69,CD80,CTLA4,CYTH4,DMXL2,EVI2B,FERMT3,FNDC3B,GABRA4,GASK1B,GLIPR1,GPRIN3,HP,IL2RG,INA,ITGAX,KLHL6,LOXL2,LY86,MMP12,MMP13,MMP19,MSR1,NRP2,PAPSS2,PTHLH,PTPN22,PTPRO,PXMP4,RAC2,SELP,SELPLG,SH2B3,SIRPB1,SPP1,VCAN,XCL1 | 44 |
| Cellular Development,Hematological System Development and Function,Lymphoid Tissue Structure and Development | Maturation of T lymphocytes | 0.00181 |  |  | CD80,RHOH,TYROBP | 3 |
| Organismal Development,Organismal Injury and Abnormalities | Abnormal morphology of thoracic cavity | 0.00185 |  |  | ADAM12,BIRC3,CD69,CTLA4,IL2RG,LOXL2,MMP12,PTHLH,PTPN22,RHOH,SELP | 11 |
| Cell-To-Cell Signaling and Interaction,Hematological System Development and Function | Signaling of T lymphocytes | 0.00196 |  |  | CTLA4,IL2RG | 2 |
| Cell Death and Survival | Apoptosis of peritoneal macrophages | 0.00196 |  |  | MSR1,NCEH1 | 2 |
| Cell-To-Cell Signaling and Interaction,Hematological System Development and Function,Hematopoiesis,Immune Cell Trafficking,Inflammatory Response | Activation of thymocytes | 0.00196 |  |  | IL2RG,PTPN22 | 2 |
| Cell-To-Cell Signaling and Interaction | Association of cells | 0.00196 |  |  | MSR1,TYROBP | 2 |
| Cancer,Dermatological Diseases and Conditions,Hematological Disease,Immunological Disease,Organismal Injury and Abnormalities | Sézary syndrome | 0.002 |  |  | CXCL13,IL2RG,SELPLG | 3 |
| Cellular Movement,Hematological System Development and Function | Cell movement of hematopoietic progenitor cells | 0.002 |  |  | CD69,CXCL13,SELPLG | 3 |
| Cancer,Organismal Injury and Abnormalities | Pelvic cancer | 0.00201 |  |  | ADAMDEC1,BCAT1,BIRC3,CCNA1,CD37,CD69,CD80,CTLA4,CYTH4,DMXL2,FERMT3,FNDC3B,GABRA4,GASK1B,GLIPR1,GPRIN3,HP,IL2RG,INA,ITGAX,LOXL2,LY86,MMP12,MMP13,MMP19,MSR1,NRP2,PAPSS2,PARVG,PTPN22,PTPRO,PXMP4,RAC2,SELP,SELPLG,SH2B3,SIRPB1,SPP1,VCAN,XCL1 | 40 |
| Cancer,Hematological Disease,Immunological Disease,Organismal Injury and Abnormalities | Angioimmunoblastic T-cell lymphoma | 0.00204 |  |  | CXCL13,ITGAX,MMP12,SELP | 4 |
| Connective Tissue Development and Function,Skeletal and Muscular System Development and Function,Tissue Development | Ossification of bone | 0.00208 |  |  | CLEC4A,FNDC3B,MMP13,PTHLH | 4 |
| Cell-To-Cell Signaling and Interaction,Connective Tissue Development and Function,Skeletal and Muscular System Development and Function | Activation of osteoblasts | 0.00214 |  |  | ADAM12,SPP1 | 2 |
| Connective Tissue Disorders,Developmental Disorder,Hereditary Disorder,Organismal Injury and Abnormalities,Skeletal and Muscular Disorders | Spondyloepimetaphyseal dysplasia | 0.00214 |  |  | MMP13,PAPSS2 | 2 |
| Cell-To-Cell Signaling and Interaction,Humoral Immune Response,Inflammatory Response | Immune response of B lymphocytes | 0.00214 |  |  | CD69,CD80 | 2 |
| Gastrointestinal Disease,Hepatic System Disease,Organismal Injury and Abnormalities | Fibrosis of liver | 0.00216 |  |  | HP,IL2RG,MMP13,SPP1 | 4 |
| Immunological Disease | Primary immunodeficiency disorder | 0.00216 |  |  | CTLA4,FERMT3,IL2RG,IRF8 | 4 |
| Cancer,Organismal Injury and Abnormalities | Head and neck squamous cell carcinoma | 0.00216 |  |  | ADAM12,CTLA4,CXCL13,CYTH4,DMXL2,GASK1B,INA,IRF8,ITGAX,MMP12,MMP13,MSR1,NRP2,P2RY8,PAPSS2,PTPRO,SIRPB1,SNAP25,SPP1,VCAN | 20 |
